# Manganese‐Catalyzed Enantioselective Dearomative Epoxidation of Naphthalenes with Aqueous Hydrogen Peroxide

**DOI:** 10.1002/anie.202504356

**Published:** 2025-05-02

**Authors:** Najoua Choukairi Afailal, Siu‐Chung Chan, Miquel Costas

**Affiliations:** ^1^ Institut de Química Computacional i Catàlisi (IQCC) and Departament de Química Universitat de Girona Campus Montilivi Girona Catalonia E‐17071 Spain

**Keywords:** Arenes, Bioinspired catalysis, Enantioselectivity, Epoxidation, Hydrogen peroxide

## Abstract

Arenes are abundantly occurring molecules of significant interest as versatile starting materials in organic reactions. Typically, oxidation of arenes yields planar molecules such as phenols and quinones. However, several iron dependent oxygenases can disrupt the aromaticity of arenes through oxidation and introduce C(sp^3^)─O stereogenic centers, resulting in precious enantioenriched epoxide or diol products. Emulating this enzymatic behavior with synthetic catalysts has met little success until now. Herein we describe a catalytic chemo‐ and enantioselective dearomative epoxidation of naphthalenes. The singular chemo‐ and enantioselectivity features of the reaction critically rely on a manganese catalyst that combines electron donating groups and steric demand on the ligand and activates hydrogen peroxide under mild conditions and short reaction times. Assisted with an *N*‐protected amino acid, this catalyst epoxidizes a range of naphthalenes providing chemically versatile diepoxides in moderate to good yields and high levels of enantioselectivity. Straightforward elaboration gives diverse access to densely functionalized 3D structurally rich oxygenated molecules. The reaction constitutes a paradigmatical example of expedient access to stereochemically rich, valuable oxygenated molecules from readily available feedstocks, enabled by highly reactive yet selective biologically inspired oxidation catalysts.

## Graphical abstract

The enantioselective epoxidation of naphthalenes into their corresponding arene oxides with aqueous hydrogen peroxide is described. The reaction combines dearomatization with installation of chemically versatile functionality in an enantioselective manner. Chemo and enantioselectivity relies in the synergy of a novel e‐rich and sterically encumbered manganese catalyst and amino acids featuring *tert*‐butyl leucine moiety as coligands.

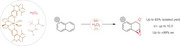

## Introduction

One of the main goals of modern synthetic organic chemistry is the development of reactions that convert readily available feedstocks into precisely functionalized molecules amenable for diverse elaboration. Single‐step reactions with high atom economy that do not require prefunctionalized substrates and produce stereochemically rich chiral products are particularly appealing. These reactions are valuable not only for producing value‐added products but also for achieving them through pathways with minimum steps and chemical waste.^[^ ^1^, ^2^, ^3^, ^4^ ^]^

**Figure 1:**
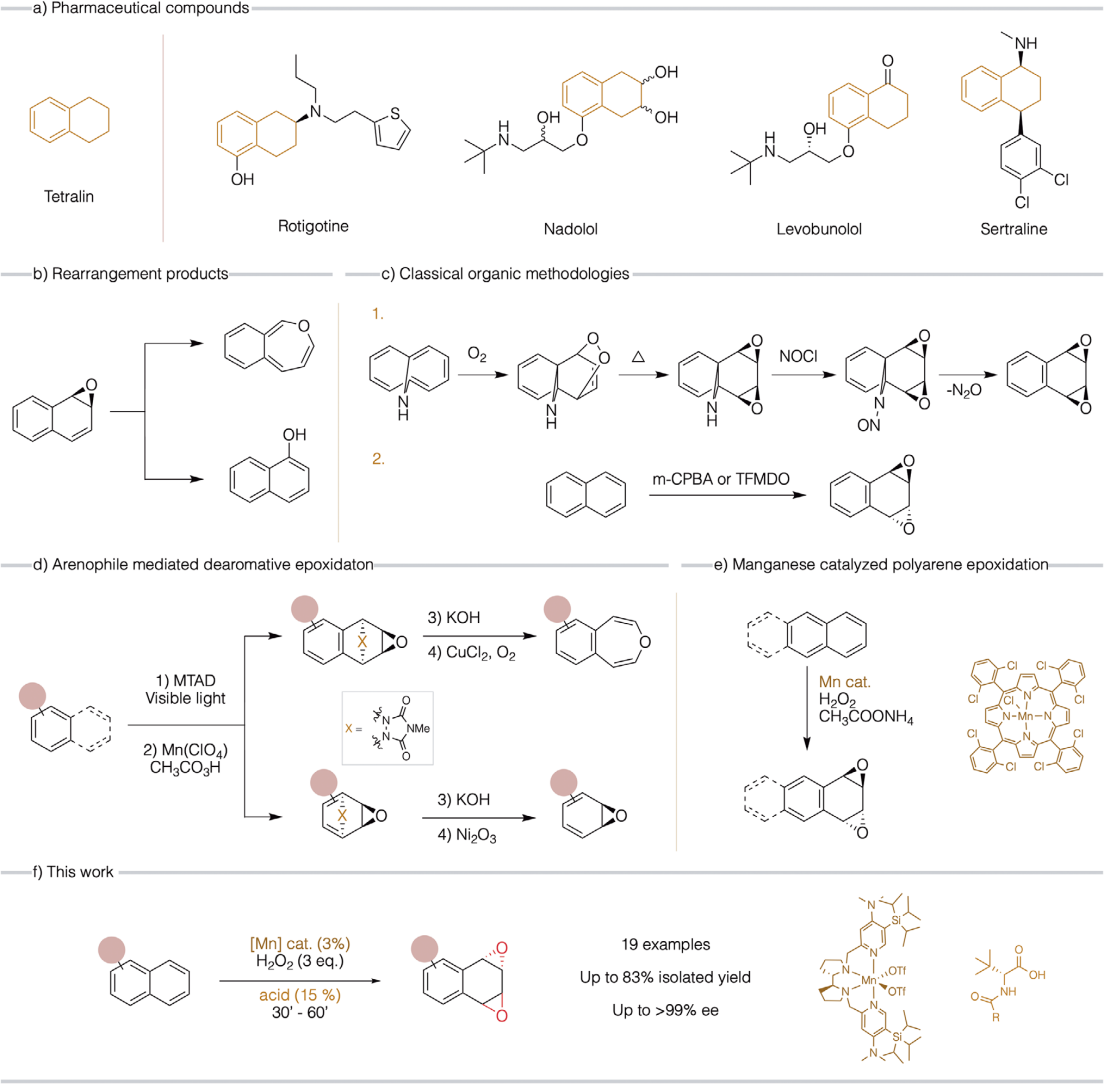
a) Pharmaceutical compounds based on a tetralin core. b) Rearrangement products of arene oxides. c) Classical organic methodologies to synthesize naphthalene oxides. d) Arenophile mediated dearomative epoxidation of arenes. e) Epoxidation of polyarenes using a porphyrin manganese catalyst. f) This work: enantioselective dearomative epoxidation of naphthalenes.

Arenes are prominent among the widely available molecules as starting materials. Their reactivity is often confined to reactions that preserve the aromaticity. However, dearomatization reactions receive increasing interest, among other reasons because of the improved pharmacokinetics of 3D dearomatized structures compared to their planar aromatic counterparts.^[^ ^5^, ^6^, ^7^, ^8^, ^9^, ^10^, ^11^, ^12^, ^13^, ^14^, ^15^ ^]^ For example, the tetralin core is present in several pharmaceutically relevant compounds such as rotigotine (Parkinson),^[^ ^16^ ^]^ nadolol (hypertension),^[^ ^17^ ^]^ levobunolol (glaucoma)^[^ ^18^ ^]^ and sertraline (antidepressive)^[^ ^19^ ^]^ (**a**, Figure 1).

Oxidative dearomatization reactions are rare but particularly valuable because they combine dearomatization with installation of oxygen atom derived functionality. The resulting products are stereochemically rich and can be manipulated through a wide range of reactions.^[^ ^20^, ^21^, ^22^ ^]^

Epoxide‐containing molecules derived from oxidative dearomatization constitute a paradigmatic case. There are very few precedents of arene epoxidation reactions, probably due to the combined challenges of the dearomatization process and the instability of the resulting arene oxides (**b**, Figure 1). Consequently, the pursuit of obtaining arene oxides through organic methodologies has grown over the years. One of the first reported examples involved a four‐step reaction mediated by light, starting with 11‐azabicyclo[4.4.1]undeca‐1,3,5,7,9‐pentaene to end up with the desired oxides (**c**, **1**, Figure 1).^[^ ^23^ ^]^ This intricate process was later streamlined into a one‐step reaction, employing naphthalene as substrate and generating the bis‐oxide in low yields through oxidation with *m*‐chloroperoxybenzoic acid (m‐CPBA) (**c**, **2**, Figure 1).^[^ ^24^ ^]^ High yield epoxidation of naphthalene requires the use of the highly electrophilic methyl(trifluoromethyl)dioxirane (TFMDO).^[^ ^25^ ^]^

A notable breakthrough was reported in recent years where a broad range of arenes were converted to epoxides via a three‐step arenophile‐mediated synthesis (**d**, Figure 1).^[^ ^26^ ^]^ In this innovative approach, the aromaticity of benzenes and naphthalenes is disrupted by a light mediated [4 + 2] cycloaddition reaction with an arenophile (azodicarbonyl 4‐methyl‐1,2,4‐triazoline‐3,5‐dione, MTAD). Subsequently, the cycloaddition olefinic product obtained can be epoxidized with a manganese catalyst. Removal of the MTAD arenophile results in the final arene epoxides in the case of phenyl substrates, while for naphthalenes only the oxepine was obtained.

Catalytic epoxidation with selected Mn‐porphyrins, biomimetic models of P450's, has been seldom described,^[^ ^27^ ^]^ (**e**, Figure 1) while most of the olefin epoxidation metal catalysts are either unreactive or deliver phenols and quinone products.^[^ ^28^, ^29^, ^30^, ^31^, ^32^, ^33^, ^34^, ^35^, ^36^, ^37^ ^]^

Except for biocatalysis using metal‐dependant oxygenases, no nonenzymatic oxidative dearomatization methods have been reported to be enantioselective so far.^[^ ^38^, ^39^, ^40^, ^41^ ^]^ But even in enzymatic reactions, the instability of these products often leads to the formation of stereochemically depleted rearranged derivatives such as oxepines,^[^ ^42^, ^43^, ^44^ ^]^ naphthols, or quinones.^[^ ^45^, ^46^ ^]^ In this context, an enantioselective arene epoxidation methodology based on small molecule catalysts will represent an extraordinarily valuable tool to convert readily available feedstocks into precious chiral oxygenated building blocks of interest in biological and pharmaceutical chemistry.

Manganese complexes with aminopyridine ligands have emerged as extraordinarily powerful oxidation catalysts, exhibiting exceptional epoxidation capabilities.^[^ ^47^, ^48^, ^49^ ^]^ Chiral versions of this class of catalysts deliver high enantioselectivities in the epoxidation of a broad scope of olefins and utilize hydrogen peroxide as oxidant, particularly interesting from the perspective of atom economy and environmental impact.^[^ ^50^, ^51^, ^52^, ^53^ ^]^ We reasoned that the high electrophilicity of the high‐valent manganese‐oxo species operating in these catalysts may enable them to overcome the chemical inertia of arenes, providing enantioenriched epoxides. Furthermore, the mild experimental conditions and short reaction times in which these catalysts operate may limit the decomposition of the highly reactive epoxide products. Building on these considerations, we describe an enantioselective one‐step method for oxidizing naphthalenes into the corresponding arene oxides (**f**, Figure 1). To achieve this goal, a chiral manganese catalyst that combines electronic and steric elements was developed. In combination with an *N*‐protected amino acid, the catalyst activates aqueous hydrogen peroxide and performs the enantioselective epoxidation of a broad range of naphthalenes. Finally, the resulting oxides were subjected to derivatization to showcase their versatility in organic synthesis, illustrating their potential utility.

## Results and Discussion

### Reaction Development

**Figure 2:**
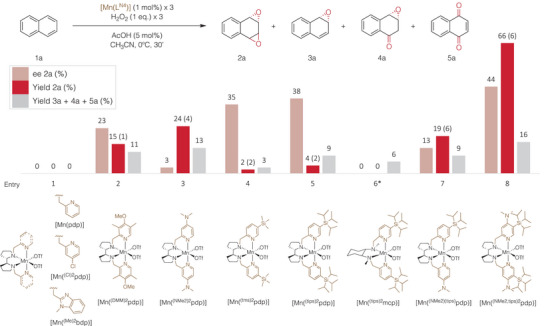
Catalyst screening. Yields determined by ^1^H‐NMR. Enantioselectivities determined by SCF‐HPLC. [2*^anti^*/2*^syn^*] ratio (in parentheses) determined by ^1^H‐NMR from the crude mixture. Average of two runs, replicates are included in Table S7. *1% of 1‐naphthol formation.

Catalytic epoxidation of naphthalene with hydrogen peroxide was first screened using different previously reported manganese catalysts [Mn(CF_3_SO_3_)_2_(L^N4^)], [Mn(L^N4^)], where L^N4^ stands as a aminopyridine tetradentate ligand (Figure 2). Following an initial optimization process (see Supporting Information for details) the following iterative protocol was adopted. In a typical experiment, an acetonitrile solution of hydrogen peroxide (1 equiv.) was delivered by syringe pump during 10 min to a stirred solution of the catalysts (1 mol%), acetic acid (5 mol%), and substrate (0.12 M) in acetonitrile placed in an open‐to‐air vessel at 0 °C. A second addition of catalyst (1 mol%) was followed by a second addition of peroxide (1 equiv.), and the process was repeated with a third addition of catalyst and peroxide. Following the three cycles of peroxide addition, the mixture was filtered through a short plug of basic alumina, the solvent was removed under vacuum and finally an internal standard was added to further analyze the mixture by ^1^H‐NMR.

Under these reaction conditions, the simplest catalysts [Mn(^(Me)2^bdp)], and [Mn(pdp)], containing benzimidazole and pyridine heterocycle donors, respectively didn't provide detectable amounts of epoxide products, and the same occurred with [Mn(^(Cl)2^pdp)] where the pyridine donors contain electron‐withdrawing chloride substituents at 4‐position (Entry 1, Figure 2). On the opposite, catalysts where ligands contain pyridine donors bearing electron‐donating substituents at 4‐position provided measurable amounts of epoxides, with yields that improve as the electron‐donating character of the substituent increases. Although [Mn(^(DMM)2^pdp)] yielded moderate epoxide formation (15%, Entry 2) and *anti/syn* selectivity on diepoxide product (d.r. (*anti/syn*): 1, Entry 2), the performance of [Mn(^(NMe2)2^pdp)] was notable (24% yield, d.r. (*anti/syn*): 4, Entry 3). In all cases, monoepoxide **3a** was obtained as a minor product while diepoxide **2a** is the major. In addition, epoxide‐ketone **4a** and naphthoquinone **5a** were obtained as minor side products. Preferential accumulation of the diepoxide **2a** over monoepoxide **3a** at low substrate conversion levels reflects the expected more facile epoxidation of the olefinic site in the first formed dearomatized epoxide alkene versus the aromatic sites in the starting arene substrate. In addition, the second epoxidation event takes place preferentially at the opposite face of the ring, yielding the chiral *anti*diepoxide **2a*^anti^***. Unfortunately, **2a*^anti^*** forms with very low enantioselectivity (3% *ee*, Entry 3). With the use of catalysts containing sterically demanding substituents on the pyridine ([Mn(^(tms)2^pdp)] and [Mn(^(tips)2^pdp)]), the enantioselectivity of the epoxidation significantly increased (35% and 38% *ee*, Entries 4 and 5), notwithstanding product yields were significantly reduced (2% and 4%) as well as the selectivity (d.r. (*anti/syn*): 2 in both cases). On the other hand, the replacement of the chiral bis‐pyrrolidine backbone of the ligand by the *trans*‐1,2‐cylohexanediamine [Mn(^(tips)2^mcp)], related to the powerful [Mn(OTf)_2_(mcp)] epoxidation catalyst described by Stack,^[^ ^47^, ^48^, ^49^ ^]^ yielded negligible amounts of diepoxide product and minimal formation of other oxidation products (6%, Entry 6).

Armed with these findings, we reasoned that a promising avenue involved synergistically combining the steric effects of [Mn(^(tips)2^pdp)] in product yields with the impact of electronic effects of [Mn(^(NMe2)2^pdp)] in enantioselectivity. Thus, [Mn(^(NMe2)(tips)^pdp)] (Entry 7) was synthesized. This complex features a 4‐substituted pyridine with a dimethylamine group, while the second pyridine ring embodies a triisopropylsilyl moiety that was supposed to enhance enantioselectivity. Unfortunately, this complex failed to achieve yields comparable to [Mn(^(NMe2)2^pdp)] or enantioselectivity akin to [Mn(^(tips)2^pdp)]. Instead, it yielded an intermediate result, achieving 13% yield with 19% ee (Entry 7). Despite disappointing, the result reinforced the generality of the trends initially observed and interestingly the selectivity toward *anti*‐isomer was the highest obtained till then (d.r. (*anti/syn*) = 6, Entry 7, Figure 2).

Faced with these less‐than‐ideal results, our final avenue was to further elaborate the complex structure, harmonizing both steric and electronic effects within the same pyridine ring. This endeavor led to the design of catalyst [Mn(^(NMe2,tips)2^pdp)]. The activity of this complex stood out among the other catalysts, delivering commendable diepoxide **2a** yields (66%, Entry 8, Figure 2) retaining the selectivity toward *anti*‐isomer as with complex [Mn(^(NMe2,tips)^pdp)] (d.r. (*anti/syn*) = 6, Entry 8, Figure 2) along with promising enantioselectivity (44% *ee*). These results represent the most favorable outcomes obtained from the initial catalyst screening process and therefore [Mn(^(NMe2,tips)2^pdp)] was chosen for future reaction development. Of notice, the dominant epoxidation chemoselectivity exhibited by the catalyst is quite notable, and sharply contrasts with most of the known naphthalene oxidation methods, which provide naphthol and naphthoquinone as products.^[^ ^28^, ^29^, ^30^, ^31^, ^32^, ^33^, ^34^, ^35^, ^36^, ^37^ ^]^

After identifying the optimal catalyst, the reaction was then explored in different solvents (see Supporting Information for details). Unfortunately, yields and enantioselectivities could not be improved.

**Figure 3:**
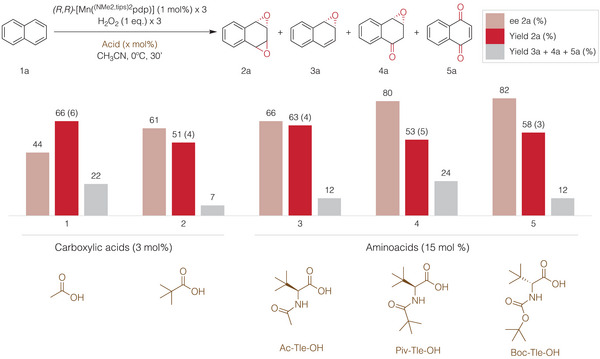
Carboxylic acid and amino acid screening. Yields determined by ^1^H‐NMR. Enantioselectivities determined by SCF‐HPLC. [2*^anti^*/2*^syn^*] ratio (in parentheses) determined by ^1^H‐NMR from the crude mixture.

Subsequently, exploration of carboxylic acids was undertaken to assess potential improvements in chemo‐ and enantioselectivities (see Tables S6–S8 in Supporting Information and Figure 3). Effectively, replacing acetic acid by pivalic acid led to a substantial improvement in enantioselectivity up to a promising 61% *ee*, however a loss of diatereoselectivity and yield was produced (d.r: 4, 51% yield, Entry 2). After testing several alkyl carboxylic acids, structurally richer *N*‐protected amino acids were then examined.^[^ ^54^, ^55^, ^56^ ^]^ Pleasantly, the use of these amino acids generally resulted in higher enantioselectivities compared to alkyl carboxylic acids. Additionally, when chiral amino acids were used, match‐mismatch effects between the chirality of the amino acid and of the manganese catalyst became apparent, as shown in Tables S10, S13, S14 in Supporting Information. Figure 3 presents the most favorable results. Of notice, in all cases, the major enantiomer is determined by the chirality of the manganese catalyst.

Using (*S*,*S*)‐[Mn(^(NMe2,tips)2^pdp)] as catalyst, N‐protected *tert*‐Leucine amino acids proved to be the best performing cocatalysts combination in terms of enantioselectivity and were therefore explored with varying protecting groups (entries 3–5 in Figure 3 show the most relevant cases). The *tert*‐butyloxycarbonyl protected D‐Tleu (Boc‐Tle‐OH) outcome surpassed the previous results, achieving **2a** in 58% yield and 82% *ee*, while overoxidized product formation was reduced to 12% (Entry 5).

Remarkably, when Boc‐Tle‐OH amino acid was combined with lutidine (15 mol%), the enantioselectivity improved up to an excellent 94% *ee*, while epoxide yields and chemoselectivities remained consistent (Entry 3, Table S14 in Supporting Information). However, the use of Boc‐Tle‐OH amino acid significantly diminished *anti*‐isomer selectivity of **2a** (d.r. 3) when compared to the use of acetic acid (d.r. 6). Of notice, this decrease in d.r. is generally observed when lutidine was introduced to reactions involving various carboxylic acids (Table S14 in Supporting Information). Nevertheless, because of the high enantioselectivity obtained with a combination of Boc‐Tle‐OH and lutidine (15 mol%), these conditions were used to explore the substrate scope (Method A, Figure 5).

In addition, we further explored the use of Piv‐Tle‐OH with the aim to identify a reaction that provides a satisfactory combination of good yield of **2a**, with optimum levels of enantio and diastereoselectivity. As shown in Table S10 and Figure 3, the use of Piv‐Tle‐OH gave the highest d.r. among all the Tle derived amino acids tested (Entry 4, Figure 3) with yield and enantioselectivity that are comparable to those achieved with Boc‐Tle‐OH (Entry 5, Figure 3). When lutidine was combined with Piv‐Tle‐OH, it only led to a marginal increase of *ee* from 80% to 84% but significantly decreased the diastereoselectivity from 5 to 2 (Entries 12 and 13, Table S14).

It is well known that naphthalene can engage in various noncovalent π interactions, and π‐π interactions with aromatic compounds being the most studied one.^[^ ^57^, ^58^, ^59^ ^]^ In this context, we studied the effect of aromatic solvents on the reaction (Table S16–S19 in Supporting Information), given their reported enhancement on diastereoselectivity and enantioselectivity in organocatalysis.^[^ ^60^ ^]^ Pleasantly, the use of a 1:1 mixture of CH_3_CN and deuterated benzene (C_6_D_6_) increased enantioselectivity from 80% to 86% ee, while maintaining a comparable yield (55%) and anti‐isomer selectivity (d.r. 5) of 2a. Of notice, this cosolvent system (1:1 CH_3_CN/C_6_D_6_) also had a similar positive impact on enantioselectivity when used with acetic acid and Boc‐Tle‐OH (Tables S5, S15). Notably, extending H_2_O_2_ addition time increased the yield of **2a** from 66% to 87% under standard condition using acetic acid in CH_3_CN at 0 °C (Entry 3, Table S19). Ultimately, after extensive optimization the optimal conditions were established for exploring the substrate scope (Method B, see below) using Piv‐Tle‐OH in 3:1 CH_3_CN/C_6_D_6_ at −20 °C with 60 min total addition time of H_2_O_2_.

### Mechanistic Considerations

**Figure 4:**
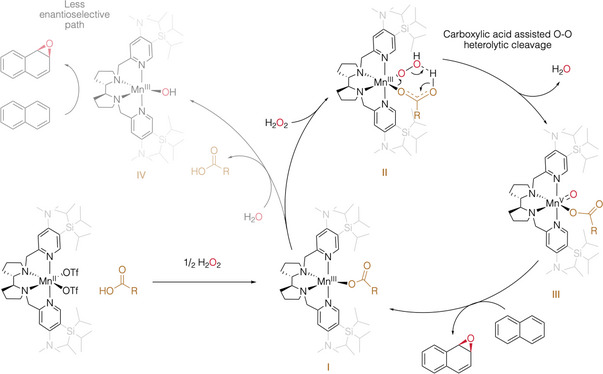
Proposed mechanism for arene epoxidation where second epoxidation step is not shown. The proposal is based in the DFT‐computed mechanism for epoxidation with related manganese catalyst‐carboxylic acid cocatalyst.^[^ ^63^, ^67^, ^68^ ^]^

The mechanism of peroxide activation and of the catalytic epoxidation of olefins and oxidation of aliphatic C─H bonds with related manganese catalysts has been amply explored in recent years.^[^ ^61^, ^62^ ^]^ The excellent activity of (*S*,*S*)‐[Mn(^(NMe2,tips)2^pdp)] in combination with *N*‐protected amino acids can be rationalized by considering the mechanism of peroxide activation previously proposed for aminopyridine manganese catalysts assisted by carboxylic acids.^[^ ^63^ ^]^ The current understanding is that carboxylic acids bind to the metal catalyst and orchestrate the activation of hydrogen peroxide by facilitating the heterolytic cleavage of the O─O bond (**II** in Figure 4). The carboxylate moiety remains then as a ligand of the metal oxo oxidant (**III**), and therefore contributes to defining of the structure, reactivity, and selectivity of these species. Furthermore, literature precedents provide a basis to understand the combined positive effects exerted by the electron donating and steric demand of the ligand in the catalytic behavior of (*S*,*S*)‐[Mn(^(NMe2,tips)2^pdp)]. Activation of H_2_O_2_ is accelerated by using electron rich catalysts (such as [Mn(^(NMe2)2^pdp)]), which can then operate in combination with small catalytic amounts of alkyl carboxylic acids.^[^ ^63^, ^64^, ^65^ ^]^ Amino acids can be used instead of alkyl carboxylic acids, and their chemical diversity offers a structurally versatile tool to shape the structure of the manganese‐oxo‐carboxylato ligand.^[^ ^54^, ^55^, ^56^ ^]^ The high electrophility of the manganese‐oxo carboxylate species then leads to epoxidation of naphthalene, overcoming its aromaticity in the enantio‐differentiating step. First formed epoxide product can then undergo a second epoxidation by a second manganese‐oxo species. Preferential formation of *anti*diepoxide diastereoisomers (vide infra) presumably reflects steric protection of the *syn*‐face exerted by the first formed epoxide moiety. On the other hand, the positive role of the tips groups in securing high enantioselectivity in oxidation reactions with this class of catalysts finds precedents.^[^ ^56^, ^66^ ^]^ Combination of both electronic and steric demand of the manganese catalyst is required for its singular activity.

Adding to this scenario, the remarkable improvement on enantioselectivity by gained using lutidine can be attributed to the ability of this base to deprotonate the amino acid, facilitating the binding of the carboxylate moiety to the metal center, thus enhancing enantioselectivity control by reducing the epoxidation contribution from a carboxylate free form of the catalyst. For this reason, other organic and inorganic bases were tested in the reactions (Table S14) but none of them surpass lutidine performance. Consistent with this scenario, when the reaction was conducted in the absence of carboxylic acid, the epoxide was obtained in 25% yield and a modest 38% *ee* (Entry 1, Table S9). Although detrimental in terms of enantioselectivity, an aspect that deserves special attention is the unexpected unique ability of [Mn(^(NMe2,tips)2^pdp)] to activate H_2_O_2_ even without the aid of a carboxylic acid.^[^ ^69^ ^]^

### Substrate Scope

**Figure 5:**
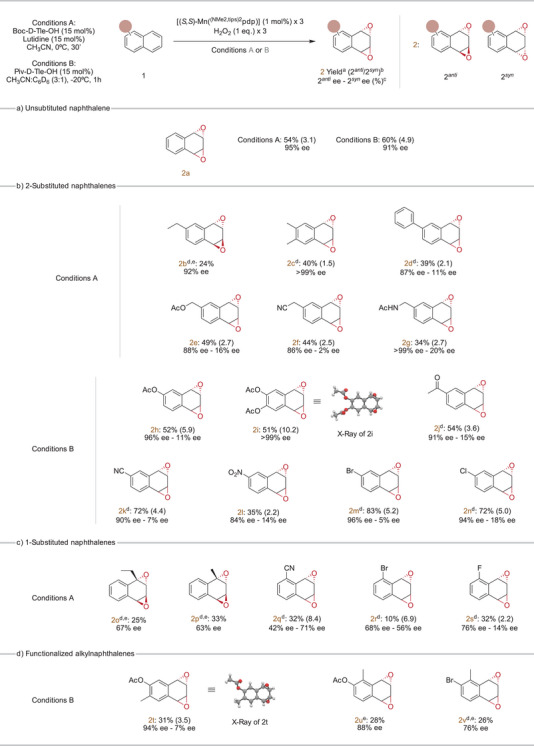
Substrate scope of different naphthalene derivatives for **2** formation. a) Isolated yields. Absolute configuration was determined by comparing the optical rotation with literature.^[^ ^70^ ^]^ Reaction conditions: 1 equiv substrate, 3 mol% catalyst (upon three additions during the 30′ or 1 h of the reaction time), 3 equiv H_2_O_2_ (upon three additions during the 30′ or 1 h of the reaction time), Conditions A: 15% Boc‐D‐Tle‐OH, 15% Lutidine, CH_3_CN, 30′, and 0 °C, Conditions B: 15% Piv‐D‐Tle‐OH, CH_3_CN:C_6_D_6_ (3:1), 1 h, and −20 °C. b) [2*^anti^*/2*^syn^*] ratio (in parentheses) determined by ^1^H‐NMR from the crude mixture. c) Enantiomeric excess *anti*‐isomer – Enantiomeric excess *syn*‐isomer (%) determined by SFC‐HPLC. d) Isolated yield through azide derivatization. e) *syn* isomer could not be isolated due to decomposition. a) Unsubstituted naphthalenes. b) 2‐Substituted naphthalenes. c) 1‐Substituted naphthalenes. d) Functionalized alkylnaphthalenes.

As earlier discussed, two different conditions (A and B, Figure 5) were established from optimizing the enantioselective epoxidation of naphthalene (**1a**). Conditions A delivered isolated yield of diepoxide (**2a**) in 54% (d.r. = 3.1) with excellent enantioselectivity (95% *ee*) (reaction performed in a 0.2 g scale), while conditions B offered higher isolated yield of **2a** in 60% and diastereoselectivity (d.r. = 4.9) with slightly lower enantioselectivity (91% *ee*). These two conditions were further advanced for testing a range of naphthalene derivatives. Each substrate was evaluated by both conditions (Tables S21, S22 for condition A and B respectively in Supporting Information), and the best results are presented in Figure 5 on the basis of yield, enantioselectivity, and diastereoselectivity.

**Figure 6:**
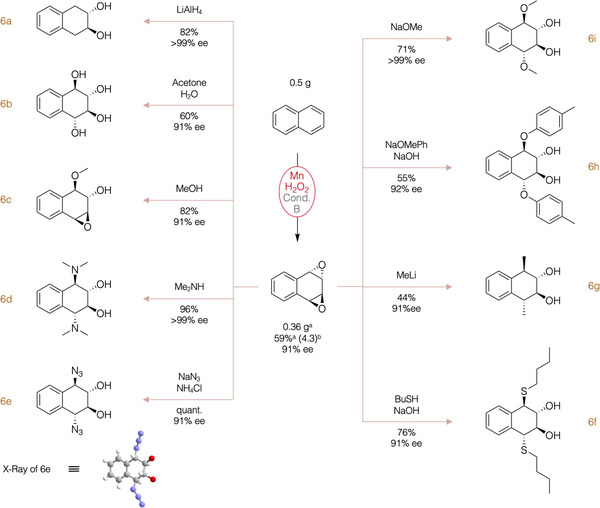
Derivatization of 2a to produce different molecules and prove the versatility of the products for further elaboration. Enantiomeric excesses determined by SFC‐HPLC. a) Isolated yield corresponding to the mixture of the isomers *syn* and *anti*. b) [2*^anti^*/2*^syn^*] ratio determined by ^1^H‐NMR from the crude mixture.

Given the reported instability of these arene oxides, yields were determined through epoxide derivatization to yield the corresponding bis‐hydroxide bis‐azide derivative (**6e**, Figure 6). This derivatization enabled quantitative conversion of the epoxide. For naphthalene, under conditions A, the isolated yield of **6e** was quantitative, and enantioselectivity remained consistently high (91% *ee*).

Naphthalenes bearing alkyl and aryl substituents in 2‐position of the arene were only examined in detail under conditions A, as oxidation of these substrates by conditions B, resulted in very low yields of the desired diepoxide products (<10%, see Table S22 in Supporting Information). Epoxidation of naphthalenes with alkyl groups (**2b** and **2c**) proceeded with excellent enantioselectivities (92% and >99% *ee*, respectively) at the nonsubstituted ring, though yields were modest, particularly for 2‐ethylnaphthalene (24% **2b**). The *syn*‐isomer of 2‐ethylnaphthalene, detected in small amounts by ^1^H‐NMR of the crude mixture, could not be isolated due to its degradability, leading to only the *anti*‐isomer being obtained. Preferential epoxidation at the nonsubstituted, and electronically poorer ring suggests steric control of the arene site selectivity. Additionally, products resulting from oxidation of the alkyl chain were not spectroscopically detected in crude mixtures, suggesting that the oxidation targets selectively the aromatic π system over C_sp3_─H bonds. However, the poor mass balance of the reactions requires caution interpretation of this conclusion. In order to further address this question, we performed the competitive oxidation of naphthalene in the presence of equimolar amounts of either cumene (BDE of the tertiary C_sp3_─H bond is 84.8 kcal mol^−1^) and ethylbenzene (BDE of the secondary C_sp3_─H bond is 85.4 kcal mol^−1^).^[^ ^71^ ^]^ In both cases, only diepoxide (**2a**) was obtained in 49% and 63% yield, respectively (see Table S20 in Supporting Information).

As expected, 2‐phenyl substituted naphthalene (**2d**, Figure 5) was epoxidized exclusively at the nonsubstituted ring that yielded the corresponding diepoxide with a modest yield of 39% and diastereoselective of 2.1. Notably, the *anti*‐isomer was obtained with high enantioselectivity (87% *ee*). Substituents at position 2 render the *syn*‐isomers chiral, though in low enantioselectivity of 11% *ee*.

Unactivated naphthalenes bearing substituted alkyl groups (**1e**, **1f**, and **1g**) reacted efficiently, and unlike the reactions with 2‐ethylnaphthalene (**1b**) in this case the *syn* isomer was more stable and could be isolated. Therefore, the total yields for each product were higher than the ones obtained for 2‐ethylnaphthalene, yielding a 49% for **2e**, 44% for **2f**, and 34% of **2g**. As in the previous substrate (**2d**), for those alkyl‐substituted substrates the enantiomeric excess for *anti* isomers was excellent (88% *ee* for **2e*^anti^***, 86% *ee* for **2f*^anti^***, and >99% *ee* for **2g*^anti^***), while for *syn*‐isomers the enantioselectivities were very low (16% *ee* for **2e*^syn^***, 2% *ee* for **2f*^syn^***, and 20% *ee* for **2g*^syn^***).

Naphthalenes bearing electron‐withdrawing substituents in 2‐position were epoxidized in preparative yields and excellent enantioselectivity, specially under conditions B. Naphthyl acetates are particularly interesting examples because they result in densely functionalized oxygen rich products with stereochemically well‐defined structures. Both 2‐naphthyl acetate (**1h**) and 2,3‐naphthyl diacetate (**1i**) produced very high enantioselective (96% and >99% *ee*, respectively) oxides resulting from epoxidation at the nonsubstituted ring in moderate yields (52% **2h** and 51% **2i**, Figure 5). The pronounced diastereoselectivity of 2,3‐naphthyl diacetate (**1i**), favoring the *anti*‐isomer over the *syn* with a ratio of 10.2, was also noteworthy. For the other electron‐withdrawing group‐containing naphthalenes (**1j**, **1k**, **1l**, and Figure 5), the diastereoselectivity was less pronounced (*anti/syn*; 3.6, 4.4, and 2.2, respectively). Nevertheless, enantioselectivities for the *anti*‐isomer remained high (91% *ee* **2j**, 90% *ee* **2k**, and 84% **2l**) while the *syn*‐isomer was largely racemic (15% *ee* for **2j**, 7% *ee* **2k**, and 14% **2l**). The functional groups covered feature arene‐carbon, arene‐oxygen, and arene‐nitrogen bonds, representing a versatile handle for further elaboration. An even more interesting situation in terms of enantioselectivity was observed when halogenated substrates were employed. Epoxidation again takes place at the nonsubstituted ring but enantioselectivities for the *anti* diastereomer was excellent (96% *ee* **2m*^anti^***, 5% *ee* **2m*^syn^***, 94% *ee* **2n*^anti^***, and 18% *ee* **2n*^syn^***). For 2‐bromonaphthalene (**1m**), the reaction yielded 83% of **2o**, while 2‐chloronaphthalene (**1n**) provided 72% of the product, constituting the highest yields achieved among the series of substrates studied. These enantioenriched products offer far reaching possibilities, as the C(sp^2^)‐halide motifs are versatile for subsequent elaboration via organometallic cross‐coupling transformations.

Substrates featuring substituents in 1‐position were also examined (**1o**–**1s**, Figure 5). In general, these substrates yielded modest yields ranging from 10% to 33%, and enantioselectivities for *anti*‐isomers were not as pronounced as in the previously described cases (42%–76% *ee*). For *syn*‐diepoxide isomers, enantioselectivities range from the poor value of 1‐fluorosubstituted **2s** (14%) to the good enantioselectivity obtained for the 1‐cyano substituted **2q** (71% ee). However, they did not match the enantioselectivities achieved with 2‐halogenated naphthalenes (**2o** and **2n**). Of notice, contrary to the epoxidation of 2‐substituted alkyl naphthalenes, the epoxidation of 1‐ethylnaphthalene (**1o**) and 1‐methylnaphthalene (**1p**) led to *ipso* products where epoxidation occurred at the alkyl substituted arene. Similarly to **2b**, the *syn*‐isomer diepoxide of 1‐ethylnaphthalene (**1o**) and 1‐methylnaphthalene (**1p**) were unstable and could not be isolated.

Finally, as aforementioned, conditions B exhibited poor yields for substrates containing alkyl groups. To investigate further, we tested substrates featuring methyl substituent along with electron‐withdrawing substituents at 2‐position on the same aromatic ring (**1t** to **1v**) under condition B. Our aim was to determine whether these substrates would exhibit poor performance like alkyl/aryl‐containing substrates (**1b**–**1d**, **1l**, and **1p**), or provide satisfactory yields and enantioselectivities similar to 2‐substituted substrates with electron‐withdrawing groups (**1h** to **1n**). Unlike alkyl/aryl‐containing substrates that only yielded <10% of the diepoxide products under condition B, these ambiphilic‐disubstituted substrates offered 26% to 31% isolated yields for *anti*‐isomers of the diepoxides (**2t*^anti^***: 31%; **2u*^anti^***: 28%; and **2v*^anti^***: 26%). The results thus show that the presence of electron‐withdrawing substituents is beneficial for this diepoxidation process under condition B. For **1t** and **1u** that contain both acetate and methyl substituents, enantioselectivity of their *anti*‐isomers, **2t*^anti^***, and **2u*^anti^*** remained high with 94% and 88% *ee* respectively, which are comparable to the high *ee* observed in 2‐naphthyl acetate (**2h*^anti^***: 96% *ee*). In contrast, a significant decrease in enantioselectivity was observed for 2‐bromo‐1‐methylnaphthalene (**2s*^anti^***: 76% *ee*) compared to 2‐bromonaphthalene (**2o*^anti^***: 96% *ee*). Additionally, another 16% of *ipso*‐diepoxide derived product was isolated from this substrate (Sections 7 and 9 in Supporting Information). Akin to the instability of *syn*‐isomers (**2*^syn^***) observed in alkyl containing substrates, the isolation of **2t*^syn^***, **2u*^syn^***, and **2v*^syn^*** were unsuccessful.

### Product Elaboration

Finally, we aimed to illustrate the exceptional chemical versatility of these chiral arene oxides. Epoxides possess the capacity for straightforward modifications, delivering a wide variety of organic molecules using well‐established methodologies,^[^ ^72^, ^73^, ^74^ ^]^ which makes their transformation particularly attractive due to its ease. With this perspective, naphthalene was subjected to epoxidation in large scale (0.5g) under conditions **B**, delivering diepoxide **2a** in 59% yield (0.36g), 91% *ee*, *syn/anti* 4.3. The *anti*diepoxide **2a** was then subjected to various ring‐opening transformations either retaining the original high enantioselectivity, or alternatively in some cases the enantioselectivities were enhanced due to the purification through crystallization.^[^ ^75^ ^]^ Reduction using lithium aluminium hydride yielded the corresponding diol (**6a**, 82% yield, Figure 6). Alternatively, stirring the epoxide in a water‐acetone mixture resulted in the corresponding tetraol (**6b**, 60% yield, Figure 6). Desymmetrizative elaboration of the two epoxide units in the molecule was simply accomplished by reaction with methanol, leading to **6c** with a commendable yield of 82%. This compound's distinct functional groups, amenable to independent modification, make it a particularly valuable candidate for further elaboration.

Introduction of C─N bonds was achieved through diverse methodologies to yield either an amine (**6d**) or an azide (**6e**). The latter facilitated functionalization of various naphthalene derivatives, demonstrated in Figure 5, with remarkable conversions exemplified by **6e** (quantitative yield, Figure 6).

Reaction of the diepoxide **2a** with thiol nucleophiles yielded C─S bonds, exemplified by **6f** (76%, Figure 6), while reacting **2a** with methyllithium generated C─C bonds, as evident in **6g** (44%, Figure 6). Finally, diols with ether moieties at position 1‐ and 4 were synthesized by reacting the epoxide with sodium alkoxides. Employing sodium *p*‐methylphenolate yielded **6h** with a 55% yield, whereas sodium methoxide resulted in **6i** with a 71% yield (Figure 6).

These standard manipulations demonstrate the versatility of the generated chiral arene oxides, Furthermore, the presence of a wide diversity of additional functional groups in the nonoxidized rings offer additional handles for chemical elaboration.

## Conclusions

A methodology for single‐step enantioselective epoxidation of naphthalenes into their corresponding arene oxides has been developed. Product yields, chemo‐ and enantioselectivity of the reaction critically rely on the synergy between: a) a novel manganese catalyst that combines sterically encumbered and highly electron‐donating properties on the ligand set; b) amino acids featuring *tert*‐butyl leucine moiety as coligands (Boc‐Tle‐OH for condition A and Piv‐Tle‐OH for condition B). Of interest and potentially far‐reaching is the impact of the amino acid coligands in defining the selectivity outcome. The relatively complex chemical nature of these amino acids likely leads to weak interactions with the substrates and a structural preorganization of the space surrounding the reactive oxo moiety, which collectively contribute in defining the chemo‐, enantio‐, and diastereoselectivity outcome. The understanding of these subtle aspects is envisioned to guide future improvements in the performance and scope of these catalysts. The resulting catalyst system activates hydrogen peroxide under mild conditions and short reaction times, generating a highly oxidizing, presumably a high valent manganese(V)‐oxo‐carboxylato species. This highly electrophilic species undergoes dearomative epoxidation of the substrates, rendering an olefinic site ready for a second epoxidation reaction. This reaction represents unprecedent enantioselective dearomatization epoxidation of naphthalenes, combining high chemoselectivity with enantioselectivity. Notably, it enables single‐step conversion of readily accessible naphthalenes into densely functionalized chiral building blocks for further diversification. In addition, the use of excess substrate is not necessary, further increasing its synthetic utility. Furthermore, the use of an earth‐abundant metal catalyst and hydrogen peroxide as traceless oxidant underscores sustainability perspective of the reactions. The straightforward conversion of the readily available flat aromatic structure of arenes into 3D structurally and functional group rich molecules described in this work holds significance for pharmaceuticals development and molecular design, promising impactful applications in synthetic organic chemistry. Although challenges remain, this work may represent a pioneering achievement based on synthetic small molecule catalysts, providing access to reactions thought to be the exclusive domain of enzymatic processes.^[^ ^76^ ^]^

## Supporting Information

Materials and methods describing preparation of complexes and substrates, characterization, and experimental procedures for the catalytic reactions. Crystallographic data CCDC 2288020 ([Mn(^(NMe2,tips)2^pdp)]), 2298139 (2i*^anti^*), 2288019 (6e*^anti^*), and 2359644 (2t*^anti^*) contain the supplementary crystallographic data for this paper. These data can be obtained free of charge from the Cambridge crystallographic data centre and Fachinformationszentrum Karlsruhe via www.ccdc.cam.ac.uk/structures. NMR Spectra. HPLC traces.

## Conflict of Interests

The authors declare no conflict of interest.

## Supporting information



Supporting Information

## Data Availability

The data that support the findings of this study are available in the Supporting Information of this article.
