# Supplementary material for: Manganese‐Catalyzed Enantioselective Dearomative Epoxidation of Naphthalenes with Aqueous Hydrogen Peroxide
Source: Angew Chem Int Ed Engl. 2025 May 2;64(26):e202504356. doi: 10.1002/anie.202504356 (PMC12184291; doi:10.1002/anie.202504356)
Supplement: Supplementary file 1 — Supporting Information [file ANIE-64-e202504356-s001.docx]

Supporting information for

**Manganese-Catalyzed Enantioselective Dearomative Epoxidation of Naphthalenes with Aqueous Hydrogen Peroxide**

**Najoua Choukairi Afailal, Siu-Chung Chan and Miquel Costas***

*Institut de Química Computacional i Catàlisi (IQCC) and Departament de Química, Universitat de Girona, Campus Montilivi, Girona E-17071, Catalonia, Spain.*

*e-mail: [miquel.costas@udg.edu](mailto:miquel.costas@udg.edu)*

[1. Experimental section 3](#_Toc169677618)

[1.1. Materials 3](#_Toc169677619)

[1.2. Instrumentation 3](#_Toc169677620)

[1.3. Warnings 4](#_Toc169677621)

[2. Ligand synthesis 5](#_Toc169677622)

[3. Synthesis of the complexes 6](#_Toc169677623)

[4. Synthesis of substrates 8](#_Toc169677624)

[4.1. Substrates characterization data 8](#_Toc169677625)

[5. Catalytic studies 12](#_Toc169677626)

[5.1. Reaction conditions for epoxidation of naphthalenes 12](#_Toc169677627)

[5.2. Workup of the catalysis 12](#_Toc169677628)

[5.3. Epoxide opening 13](#_Toc169677629)

[5.4. Optimization 13](#_Toc169677630)

[6. General procedure for isolation of epoxidation products 36](#_Toc169677631)

[6.1. Epoxide formation 36](#_Toc169677632)

[6.2. Workup of the catalysis 36](#_Toc169677633)

[6.3. Epoxide opening 36](#_Toc169677634)

[7. Characterization of isolated products 43](#_Toc169677635)

[8. Product derivatization 66](#_Toc169677636)

[9. NMR 70](#_Toc169677637)

[10. SCF and UV-Vis 239](#_Toc169677638)

[11. Bibliography 337](#_Toc169677639)

# Experimental section

## Materials

Reagents, substrates, and solvents used were of commercially available reagent quality unless stated otherwise. Solvents were purchased from SDS, Aldrich, Fluorochem and Scharlab. Solvents were purified and dried by passing through an activated alumina purification system (M-Braun SPS - 800) or by conventional distillation techniques. All liquid substrates were passed through a silica, a basic alumina and a celite plug before being used.

## Instrumentation

Oxidation products were identified by ^1^H and ^13^C{^1^H}-NMR analyses.

NMR experiments were recorded on a Bruker 400 MHz Avance III HD spectrometer equipped with a 5mm BBOF probe. NMR spectra were performed with standard experiments provided in the Bruker release. Pulse sequences used were zg30 and zgpr in ^1^H spectra, cosygpqf and cosygpprqf in COSY spectra, hsqcedetgpsisp2.3_bbhd in ^1^H-^13^C HSQC spectra implemented with carbon multiplicity editing and proton broadband homodecoupling during acquisition, zgpg30 ^13^C{^1^H} and deptsp135 in ^13^C{^1^H} DEPT135 spectra. Spectra were referenced to the residual proto solvents peaks or TMS (tetramethylsilane) for ^1^H. GS-MS spectral analyses were performed on an Agilent 7890A gas chromatograph interfaced with an Agilent 5975c mass spectrometer with a triple-axis detector. GC analyses were carried out on an AgilentGC-7820-A gas chromatograph (HP5 column, 30m) with a flame ionization detector. High resolution mass spectrometry (HRMS) were performed on Bruker MicrOTOF-Q II (Q-TOF) instrument with a quadrupole analyzer with positive and negative ionization mode using methanol as mobile phase and at Serveis Tècnics of the University of Girona. IR spectra were taken in a Mattson- Galaxy Satellite FT-IR spectrophotometer using a MKII Golden Gate single reflection ATR system. Elemental analyses were performed using a CHNS-O EA-1108 elemental analyzer from Fisons. X-Ray diffraction analysis were carried out on a BRUKER SMART APEX CCD diffractometer using graphite-monochromated MoKα radiation (λ = 0.71073 Å) from an X-ray Tube. Optical rotations were measured at room temperature (25º C) using a Jasco P-2000 iRM-800 polarimeter. Concentration is expressed in g/100 mL. The cell was 10 cm long with a 1 mL of capacity. Enantiomer resolutions were performed using HP-Chiral- 20B, J&W CYCLOSIL-B columns and supercritical fluid chromatography (SFC) were performed on an Agilent 1260 Infinity II SFC System using CHIRALPAK IA-3, CHIRALPAK IB-3, CHIRALPAK IC-3, CHIRALPAK IG-3 and CHIRALPAK IJ-3 columns.

## Warnings

Arene oxides are suspected carcinogens, caution needs to be taken while handling those substances.

Further elaboration of arene oxides was performed with sodium azide to increase the stability of the final compound.

Sodium azide is known to be toxic and forms explosive compounds with heavy metals and dichloromethane. It also liberates toxic hydrazoic acid gas in acidic conditions.

# Ligand synthesis

2-(chloromethyl)-*N*,*N*-dimethyl-5-(triisopropylsilyl)pyridin-4-amine was synthesized as described in the reported procedure.^1^

**^(tips,NMe2)2^pdp ligand**

**^(tips,NMe2)2^pdp** was synthetized following the reported procedure^2^. In a 15 mL vial, 1.00 g of 2-(chloromethyl)-*N*,*N*-dimethyl-5-(triisopropylsilyl)pyridin-4-amine (3.1 mmols, 2.2 eq.) and 0.40 g of (*S,S*) or (*R,R*)-2,2’-bispyrrolidine tartrate (1.4 mmols, 1eq.) were mixed with 0.78 g of NaOH pellets (9.0 mmols, 6.4 eq.) in 6 mL of H_2_O:CH_2_Cl_2_ (1:1 v:v). The mixture was left vigorously stirring at room temperature for 2 days. The organic phase was separated and the aqueous phase was extracted 3 times with dichloromethane. The solution was dried with MgSO_4_ and the solvent was removed under vacuum. The crude was purified via column chromatography on neutral alumina (hexane:AcOEt 10:1 to 1:1) to yield 0.87 g of the ligand (86% yield, 1.2 mmols) as a yellow oil.

^1^H NMR (400 MHz, Chloroform-*d*) δ 8.49 (s, 2H), 7.19 (s, 2H), 4.12 (d, *J* = 14.2 Hz, 2H), 3.50 (d, *J* = 14.2 Hz, 2H), 3.08 – 2.96 (m, 2H), 2.75 (t, *J* = 6.2 Hz, 2H), 2.67 – 2.61 (m, 2H), 2.60 (s, 12H), 2.26 (q, *J* = 8.4 Hz, 2H), 1.78 (d, *J* = 6.5 Hz, 2H), 1.72 (dt, *J* = 14.2, 7.4 Hz, 4H), 1.54 – 1.41 (m, 6H), 1.09 (dd, *J* = 7.5, 1.7 Hz, 36H).

^13^C NMR (101 MHz, Chloroform-*d*) δ 169.65, 157.38, 124.26, 114.85, 66.04, 61.49, 55.75, 46.13, 26.34, 23.77, 19.42, 12.54.

HRMS(ESI+) m/z calculated [M+H]+ 721.5743, found 721.5750.

# Synthesis of the complexes

Catalysts [(*S,S*)-Mn(pdp)],^3^ [(*S,S*)-Mn(^(Cl)2^pdp)],^4^ [(*S,S*)-Mn(^(Bz)2^pdp)],^5^ [(*S,S*)-Mn(^(DMM)2^pdp)],^4^ [(*S,S*)-Mn(^(NMe2)2^pdp)],^4^ [(*S,S*)-Mn(^(tms)2^pdp)],^6^ [(*S,S*)-Mn(^(tips)2^pdp)],^7^ [(*S,S*)-Mn(^(NMe2)(tips)^pdp)],^8^ were prepared as in the reported procedures, characterization data agreed with the reported ones.

**[Mn(^(NMe2,tips)2^pdp)]**

To a vigorously stirred solution of the ligand (0.87 g, 1.21 mmol) in THF (3 mL), Mn(OTf)_2_ (0.43 g, 1.21 mmol) was added and left stirring overnight under a nitrogen atmosphere. The next day the mixture was filtered through a celite plug and the solvent was removed under reduced pressure. The residue obtained was solved again with the [minimum volume of anhydrous CH_2_Cl_2_ under N_2_ and let to crystalize by double layer of ethyl acetate. The next day 500 mg (38% yield) of white crystals were obtained.

HRMS (ESI-TOF): m/z calculated for C_44_H_76_F_6_MnN_6_O_6_S_2_Si_2_, [M-OTf]+ 924.4565, Found 924.4588

Elemental analysis (%) for C_44_H_76_F_6_MnN_6_O_6_S_2_Si_2_ (MW: 1073.41/mol). Calculated N: 7.82, C: 49.19, H: 7.13; obtained N: 7.53, C: 47.39, H: 7.12.

FT-IR (ATR) ν, cm^-1^: 2946, 2865, 1589, 1527, 1492, 1465, 1394, 1303, 1216, 1179, 1157, 1034, 982, 882, 761, 636, 513.

X-Ray: (CCDC: 2288020)


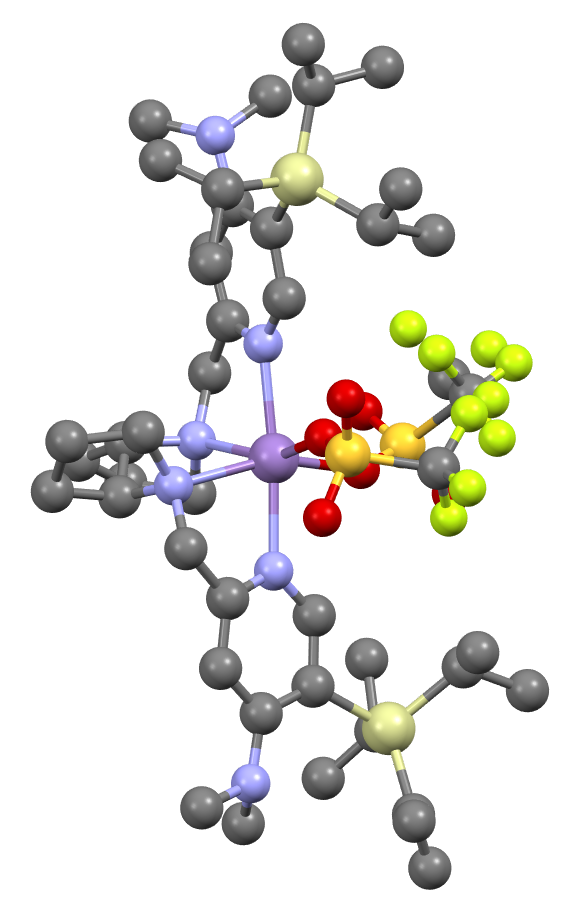


# Synthesis of substrates

Substrates **1d** and **1l** were prepared as in the reported procedures, characterization data agreed with the reported one.^9, 10^

Substrates **1h**, **1i**, **1t** and **1h** were synthesized according to the following reported procedure.^1^ In a 20 mL vial, 0.4 g of 2-naphtol or 2,3-dihydroxynaphtalene was added followed by 3 mL of methylimidazole and 5 mL of acetic anhydride. The mixture was stirred at room temperature for 2 h. After this, crushed ice was added, and the product was extracted with dichloromethane. The organic phase was washed with H_2_SO_4_ 1M, NaHCO_3_ sat., and subsequently cleaned with water, finally it was dried with MgSO_4_ and filtered through a plug of basic alumina. The solvent was removed under vacuum and the substrates were obtained with 60-70% yield as white solids and were used without further purification.

## Substrates characterization data

**Naphthalen-2-yl acetate (1h)**

^1^H NMR (400 MHz, Chloroform-*d*) δ 7.91-7.81 (m, 3H), 7.58 (d, *J* = 2.3 Hz, 1H), 7.50 (dtd, *J* = 7.9, 6.9, 5.3 Hz, 2H), 7.26 (dd, *J* = 8.9, 2.3 Hz, 1H), 2.38 (s, 3H).

^13^C NMR (101 MHz, Chloroform-*d*) δ 169.80, 148.46, 133.90, 131.61, 129.56, 127.91, 127.79, 126.70, 125.85, 121.27, 118.67, 21.35.

HRMS(ESI+) m/z calculated [M+Na]+ 209.0573, found 209.0574.

**Naphthalene-2,3-diyl diacetate (1i)**

^1^H NMR (400 MHz, Chloroform-*d*) δ 7.82 (dd, *J* = 6.2, 3.3 Hz, 2H), 7.68 (s, 2H), 7.50 (dd, *J* = 6.3, 3.2 Hz, 2H), 2.37 (s, 6H).

^13^C NMR (101 MHz, Chloroform-*d*) δ 168.69, 141.06, 131.71, 127.62, 126.51, 121.06, 20.84.

HRMS(ESI+) m/z calculated [M+Na]+ 267.0628, found 267.0627.

**2-phenylnaphthalene (1d)**

^1^H NMR (400 MHz, Chloroform-*d*) δ 8.07 (dd, *J* = 1.8, 0.8 Hz, 1H), 7.96-7.87 (m, 3H), 7.80-7.73 (m, 3H), 7.56-7.48 (m, 4H), 7.43-7.38 (m, 1H).

^13^C NMR (101 MHz, Chloroform-*d*) δ 141.30, 138.74, 133.91, 129.06, 128.64, 127.86, 127.63, 127.55, 126.48, 126.13, 126.00, 125.79.

GC-MS(CI-NH_4_^+^) m/z [M+NH_4_^+^] 222.0.

**Naphthalen-2-ylmethyl acetate (1e)**

^1^H NMR (400 MHz, Chloroform-*d*) δ 7.89 – 7.79 (m, 4H), 7.54 – 7.43 (m, 3H), 5.27 (s, 2H), 2.14 (s, 3H).

^13^C NMR (101 MHz, Chloroform-*d*) δ 171.10, 133.49, 133.34, 133.27, 128.54, 128.12, 127.86, 127.53, 126.46, 126.42, 126.05, 66.61, 21.21.

HRMS(ESI+) m/z calculated [M+Na]+ 223.0730, found 223.0736.

**Naphthalen-2-ylmethyl acetamide (1g)**

^^

^1^H NMR (400 MHz, Chloroform-*d*) δ 7.82 (dd, *J* = 9.3, 6.3 Hz, 3H), 7.72 (d, *J* = 1.7 Hz, 1H), 7.54 – 7.44 (m, 2H), 7.41 (dd, *J* = 8.4, 1.8 Hz, 1H), 4.60 (d, *J* = 5.8 Hz, 2H), 2.06 (s, 3H).

^13^C NMR (101 MHz, Chloroform-*d*) δ 166.92, 135.79, 132.93, 128.75, 127.85, 126.63, 126.49, 126.15, 126.09, 44.07, 23.52.

HRMS(ESI+) m/z calculated [M+Na]+ 222.0889, found 222.0887.

**2-nitronaphthalene (1l)**

^1^H NMR (400 MHz, Chloroform-*d*) δ 8.82 (d, *J* = 2.3 Hz, 1H), 8.25 (dd, *J* = 9.0, 2.3 Hz, 1H), 8.07 – 8.03 (m, 1H), 7.99-7.93 (m, 2H), 7.68 (dddd, *J* = 21.2, 8.2, 6.9, 1.3 Hz, 2H).

^13^C NMR (101 MHz, Chloroform-*d*) δ 129.95, 129.75, 129.49, 127.98, 127.92, 124.56, 119.17.

GC-MS(CI-NH_4_^+^) m/z [M] 173.0, [M+NH_4_^+^] 191.0.

**3-methylnaphthalen-2-yl acetate (1t)**

^1^H NMR (400 MHz, Chloroform-d) δ 7.76 (dtd, J = 6.7, 4.8, 3.9, 2.4 Hz, 2H), 7.69 (s, 1H), 7.49 (s, 1H), 7.45 – 7.39 (m, 2H), 2.38 (s, 3H), 2.35 (d, J = 1.0 Hz, 3H).

^13^C NMR (101 MHz, Chloroform-d) δ 169.66, 148.19, 132.65, 132.05, 129.68, 129.63, 127.43, 127.19, 125.85, 125.82, 119.12, 21.04, 16.92.

HRMS(ESI+) m/z calculated [M+H]+ 201.0910, found 201.0905.

**1-methylnaphthalen-2-yl acetate (1u)**

^1^H NMR (400 MHz, Chloroform-d) δ 8.01 (d, J = 8.4 Hz, 1H), 7.84 (d, J = 7.7 Hz, 1H), 7.72 (d, J = 8.8 Hz, 1H), 7.54 (ddd, J = 8.5, 6.8, 1.5 Hz, 1H), 7.47 (ddd, J = 8.1, 6.9, 1.3 Hz, 1H), 7.17 (d, J = 8.8 Hz, 1H), 2.50 (s, 3H), 2.40 (s, 3H).

^13^C NMR (101 MHz, Chloroform-d) δ 169.74, 146.31, 133.46, 131.93, 128.66, 127.45, 126.50, 125.46, 124.61, 124.28, 121.44, 21.08, 11.76.

HRMS(ESI+) m/z calculated [M+H]+ 201.0910, found 201.0916.

# Catalytic studies

## Reaction conditions for epoxidation of naphthalenes

Conditions A:

In a 3 mL vial equipped with a stir bar and the substrate (47 μmol) it was added the respective acid in 400 μL of acetonitrile, afterwards the first addition of the catalyst (0.5 mg, 0.47 μmol, 1 mol%) was performed. The mixture was cooled in an ice bath at 0ºC and a solution of H_2_O_2_ 50% aq. (79.4 μL, 0.59 M, 1 eq.) was added via syringe pump for 10 minutes.

After this, a second and a third addition of catalyst (1 mol%) and H_2_O_2_ (1 eq.) was performed under the same conditions every 10 minutes leading to the total time of the reaction up to 30 minutes.

Conditions B:

In a 3 mL vial equipped with a stir bar and the substrate (47 μmol) it was added the respective acid in 400 μL of solvent (acetonitrile:C_6_D_6_, 3:1), afterwards the first addition of the catalyst (0.5 mg, 0.47 μmol, 1 mol%) was performed. The mixture was cooled in an acetonitrile:water (3:1) bath at -20 ºC and a solution of H_2_O_2_ 50% aq. (79.4 μL, 0.59 M, 1 eq.) was added via syringe pump for 20 minutes.

After this, a second and a third addition of catalyst (1 mol%) and H_2_O_2_ (1 eq.) was performed under the same conditions every 20 minutes leading to the total time of the reaction up to 1 hour.

## Workup of the catalysis

After the catalysis was finished, an addition of 400 μL of isopropanol was performed and the solution was stirred for 1 minute, after this, the internal standard (1,3,5-trimetoxibenzene) was added and the reaction mixture was filtered through a plug of basic alumina with a very small amount of silica on the top to retain the catalyst. The solvent was removed under vacuum and analyzed by ^1^H-NMR using deuterated chloroform. NMR analysis of the solution provided substrate conversions and product yields relative to the internal standard.

## Epoxide opening

Into the rotary evaporated crude of the reaction it was added 30 mg of NaN_3_, 24 mg of NH_4_Cl and 1mL of MeOH, the reaction was left stirring overnight at 65ºC. The next day, it was added ethyl acetate and brine (3mL each one) and the organic phase was filtered through a plug of MgSO_4_. The solvent was removed under vacuum and the crude of the reaction was dissolved in 2-isopropanol HPLC grade. HPLC analysis of the solution provided the enantiomeric excess. Diastereomeric ratio was determined at this point by ^1^H-NMR of the crude of the reaction.

## Optimization

| ***Table S1.*** Temperature effect. | | | | | | |
| --- | --- | --- | --- | --- | --- | --- |
| **** | | | | | | |
| Entry^a^ | Temperature | Conv. (%) | **2a** (*anti*/*syn*) (%) | **3a** (%) | **4a** (%) | **5a** (%) |
| 1 | -40 ºC | 43 | 20 (9) | 2 | 6 | 4 |
| 2 | r.t | 99 | 65 (4) | - | 4 | 5 |
| 3 | 0ºC | 98 | 66 (6) | - | 6 | 10 |

^a^ Reaction conditions as described in Reaction conditions for epoxidation of naphthalenes and workup for the catalysis (5.1 and 5.2).

| ***Table S2.*** Number of additions. | | | | | | |
| --- | --- | --- | --- | --- | --- | --- |
| **** | | | | | | |
| Entry^a^ | Additions | Conv. (%) | **2a** (*anti*/*syn*) (%) | **3a** (%) | **4a** (%) | **5a** (%) |
| 1 | 2 | 75 | 49 (5) | 3 | 3 | 8 |
| 2 | 3 | 98 | 66 (6) | - | 6 | 10 |
| 3^b^ | 4 | 98 | 74 (6) | - | 11 | 6 |

^a^ Reaction conditions as described in Reaction conditions for epoxidation of naphthalenes and in workup for the catalysis (5.1 and 5.2). ^b^ The ^1^H-NMR of the reaction was noisy suggesting formation of multiple non identified side products.

| ***Table S3.*** Acetic acid loading. | | | | | | |
| --- | --- | --- | --- | --- | --- | --- |
| **** | | | | | | |
| Entry^a^ | AcOH (%) | Conv. (%) | **2a** (*anti*/*syn*) (%) | **3a** (%) | **4a** (%) | **5a** (%) |
| 1 | 3 | 97 | 65 (6) | 1 | 6 | 7 |
| 2 | 5 | 98 | 66 (6) | - | 6 | 10 |
| 3 | 10 | 98 | 61 (6) | - | 6 | 11 |

^a^ Reaction conditions as described in Reaction conditions for epoxidation of naphthalenes and in workup for the catalysis (5.1 and 5.2).

| ***Table S4.*** Solvent screening. | | | | | | | |
| --- | --- | --- | --- | --- | --- | --- | --- |
| **** | | | | | | | |
| Entry^a^ | | Solvent | Conv. (%) | **2a** (*anti*/*syn*) (%) | **3a** (%) | **4a** (%) | **5a** (%) |
| 1 | | Acetone | 44 | 13 (7) | 2 | - | - |
| 2 | AcOEt | | 70 | 30 (3) | 2 | - | 4 |
| 3 | | CH_2_Cl_2_ | 76 | 38 (3) | 4 | 9 | 4 |
| 4 | | CH_3_CN | 97 | 65 (6) | 1 | 6 | 7 |
| 5 | | Acetone: CH_3_CN (1:1) | 47 | 40 (6) | 2 | - | - |
| 6 | | HFIP | 100 | 22 (2) | - | 1 | 17 |
| 7 | | TFE | 100 | 42 (2) | - | 13 | 15 |

^a^ Reaction conditions as described in Reaction conditions for epoxidation of naphthalenes and in workup for the catalysis (5.1 and 5.2).

| ***Table S5.*** Effect of aromatic solvents with using acetic acid. |
| --- |

| Entry^a^ | Solvent | Conv.  (%) | **2a**  (ee)(%) | Ratio  (*anti*/*syn*) | **3a**  (%) | **4a**  (%) | **5a**  (%) |
| --- | --- | --- | --- | --- | --- | --- | --- |
| 1 | CH_3_CN | 98 | 66 (44) | 6 | - | 6 | 10 |
| 2 | Benzonitrile | 98 | 39 (63) | 4 | - | - | 4.5 |
| 3 | CH_3_CN/C_6_D_6_ (1:1) | 62 | 45 (59) | 6 | - | 1.5 | 3.5 |
| 4 | CH_3_CN/C_6_D_6_ (3:1) | 79 | 54 (56) | 6 | - | 2 | 8 |
| 5 | CH_3_CN/C_6_F_6_ (1:1) | 78 | 58 (52) | 6 | - | 1 | 4 |
| 6 | CH_3_CN/C_6_F_6_ (3:1) | 87 | 60 (50) | 7 | - | 1.5 | 8 |
| 7 | CH_3_CN/    (1:1) | 86 | 58 (48) | 6 | - | 1 | 7 |
| 8 | CH_3_CN/    (3:1) | 95 | 67 (46) | 7 | - | 1 | 9 |

^a^ Reaction conditions as described in Reaction conditions for epoxidation of naphthalenes and in workup for the catalysis and in epoxide opening (5.1, 5.2 and 5.3).

| ***Table S6.*** Effect of combined use of aromatic solvents and aromatic acids. | | | | | | | | |  |  |
| --- | --- | --- | --- | --- | --- | --- | --- | --- | --- | --- |
|  | | | | | | | | |  |  |
| Entry^a^ | | | Solvent | Acid | Conv. (%) | **2a** (ee) (%) | Ratio (*anti*/*syn*) | **3a**  (%) | **4a**  (%) | **5a**  (%) |
| 1 | | CH_3_CN/ C_6_D_6_ (1:1) |  | 89 | 49 (59) | 5 | - | - | 11 |  |
| 2 | | CH_3_CN/ C_6_D_6_ (1:1) |  | 87 | 53 (64) | 4 | - | 1 | 9 |  |
| 3 | | CH_3_CN/ C_6_D_6_ (1:1) |  | 93 | 46 (60) | 5 | - | 2 | 13 |  |
| 4 | | CH_3_CN |  | 84 | 51 (58) | 5 | - | 1 | 10 |  |
| 5 | | CH_3_CN/ C_6_D_6_ (1:1) |  | 80 | 46 (61) | 5 | - | <1 | 9 |  |
| 6 | | CH_3_CN/ C_6_F_6_ (1:1) |  | 80 | 51 (60) | 5 | - | - | 9 |  |

^a^ Reaction conditions as described in Reaction conditions for epoxidation of naphthalenes and in workup for the catalysis and in epoxide opening (5.1, 5.2 and 5.3).

| ***Table S7.*** Catalyst screening. | | | | | | | | |
| --- | --- | --- | --- | --- | --- | --- | --- | --- |
| **** | | | | | | | | |
| Entry^a^ | Catalyst | Conv. (%) | **2a** (ee) (%) | | Ratio (*anti*/*syn*) | **3a** (%) | **4a** (%) | **5a** (%) |
| 1^b^ | [Mn(^(tips)2^mcp)] | 16 | | - | - | 5 | - | - |
|  |  | 18 | | - | - | 4 | - | - |
| 2 | [Mn(^(tips)2^pdp)] | 32 | | 4 (38) | 2 | 9 | - | - |
|  |  | 40 | | 5 (38) | 2 | 8 | - | 1 |
| 3 | [Mn(^(tms)2^pdp)] | 26 | | 2 (35) | 2 | 2 | - | 1 |
|  |  | 18 | | 1 (36) | 3 | 3 | - | - |
| 4 | [Mn(^(NMe2)2^pdp)] | 41 | | 25 (3) | 4 | 2 | 9 | 1 |
|  |  | 42 | | 23 (3) | 4 | 3 | 8 | 2 |
| 5 | [Mn(^(NMe2)(tips)^pdp)] | 61 | | 20 (13) | 6 | 3 | 3 | 3 |
|  |  | 70 | | 19 (13) | 6 | 5 | - | 4 |
| 6 | [Mn(^(NMe2,tips)2^pdp)] | 98 | | 66 (44) | 6 | - | 6 | 10 |
|  |  | 99 | | 65 (43) | 6 | - | 4 | 12 |
| 7 | [Mn(^(DMM)2^pdp)] | 68 | | 14 (23) | 1 | - | 8 | 3 |
|  |  | 80 | | 16 (22) | 2 | - | 8 | 3 |

^a^ Reaction conditions as described in Reaction conditions for epoxidation of naphthalenes, workup for the catalysis and in epoxide opening (5.1, 5.2 and 5.3). ^b^1% naphthol formation.

| ***Table S8.*** Acid screening. | | | | | | | |
| --- | --- | --- | --- | --- | --- | --- | --- |
| **** | | | | | | | |
| Entry^a^ | Acid | Conv. (%) | **2a** (ee) (%) | Ratio (*anti*/*syn*) | **3a**  (%) | **4a** (%) | **5a** (%) |
| 1^b^ |  | 67 | 47 (56) | 6 | - | - | - |
| 2 |  | 67 | 40 (56) | 4 | - | - | - |
| 3 |  | 77 | 57 (50) | 4 | - | - | - |
| 4 |  | 50 | 46 (59) | 3 | - | - | - |
| 5^b^ |  | 78 | 51 (61) | 4 | - | - | 7 |
| 6 |  | 53 | 20 (55) | 8 | 3 | 8 | 9 |
| 7 |  | 89 | 29 (65) | 4 | - | 10 | 22 |
| 8 |  | 67 | 43 (54) | 5 | - | - | - |
| 9 |  | 89 | 56 (52) | 4 | - | - | 2 |
| 10 |  | 49 | 41 (55) | 8 | - | - | - |
| 11 |  | 62 | 51 (54) | 6 | - | - | - |
| 12 |  | 72 | 47 (54) | 4 | - | - | - |
| 13 |  | 91 | 67 (58) | 4 |  | - | 1 |
| 14^b^ |  | 98 | 59 (49) | 4 | - | 9 | 11 |
| 15^b^ |  | 93 | 50 (58) | 4 | - | 3 | 6 |
| 16^b^ |  | 84 | 57 (16) | 5 | - | 3 | 6 |

^a^ Reaction conditions as described in Reaction conditions for epoxidation of naphthalenes, workup for the catalysis and in epoxide opening (5.1, 5.2 and 5.3). ^b^ CH_3_CN as solvent, without acetone.

| ***Table S9.*** Acid loading. | | | | | | | | |
| --- | --- | --- | --- | --- | --- | --- | --- | --- |
| **** | | | | | | | | |
| Entry^a^ | Pivalic acid (%) | Conv. (%) | **2a** (ee) (%) | Ratio (*anti*/*syn*) | **3a** (%) | **4a** (%) | **5a** (%) |  |
| 1 | 0 | 47 | 25 (38) | 6 | 3 | 4 | 1 |  |
| 2 | 3 | 78 | 51 (61) | 4 | - | - | 7 |  |
| 3 | 5 | 94 | 57 (69) | 3 | - | - | 12 |  |
| 4 | 10 | 100 | 50 (78) | 2 | - | 2 | 12 |  |

^a^ Reaction conditions as described in *Reaction conditions for epoxidation of naphthalenes,* *workup for the catalysis* and in *epoxide opening* (5.1, 5.2 and 5.3).

| ***Table S10.*** Amino acid screening. | | | | | | | |
| --- | --- | --- | --- | --- | --- | --- | --- |
| **** | | | | | | | |
| Entry^a^ | Amino acid | Conv. (%) | **2a** (ee) (%) | Ratio (*anti*/*syn*) | **3a** (%) | **4a** (%) | **5a** (%) |
| 1 | D | 100 | 48 (75) | 2 | - | 3 | 25 |
| 2 | L | 100 | 50 (65) | 2 | - | 1 | 24 |
| 3 | L | 100 | 61 (79) | 3 | - | 3 | 11 |
| 4 | D | 100 | 62 (67) | 4 | - | 2 | 15 |
| 5 | L | 100 | 63 (66) | 4 | - | 3 | 9 |
| 6 |  L | 100 | 53 (80) | 5 | - | 2 | 22 |
| 7 | L | 28 | 22 (61) | 6 | 2 | - | - |
| 8 | L | 89 | 54 (45) | 5 | - | 1 | 16 |
| 9 | L | 84 | 48 (46) | 6 | 1 | - | 15 |
| 10 | L | 100 | 48 (62) | 3 | - | 2 | 20 |
| 11 | L | 100 | 48 (63) | 3 | - | - | 21 |
| 12 | L | 100 | 54 (62) | 3 | - | - | 18 |
| 13 |  | 100 | 50 (61) | 3 | - | - | 22 |
| 14 | L | 100 | 43 (69) | 2 | - | - | 22 |
| 15 | L | 100 | 35 (81) | 2 | - | 1 | 25 |

^a^ Reaction conditions as described in *Reaction conditions for epoxidation of naphthalenes,* *workup for the catalysis* and in *epoxide opening* (5.1, 5.2 and 5.3).

| ***Table S11.*** Solvent screening with Piv-L-*tert*-Leucine as acid. | | | | | | | | |
| --- | --- | --- | --- | --- | --- | --- | --- | --- |
| **** | | | | | | | | |
| Entry^a^ | Solvent | Conv. (%) | **2a** (ee) (%) | Ratio (*anti*/*syn*) | **3a** (%) | **4a** (%) | **5a** (%) |  |
| 1 | CH_3_CN | 100 | 53 (80) | 5 | - | 2 | 22 |  |
| 2 | Acetone:CH_3_CN (1:1) | 89 | 48 (64) | 6 | 2 | - | 13 |  |
| 3 | Acetone | 68 | 23 (67) | 5 | - | 1 | - |  |

^a^ Reaction conditions as described in *Reaction conditions for epoxidation of naphthalenes,* *workup for the catalysis* and in *epoxide opening* (5.1, 5.2 and 5.3).

| ***Table S12.*** Base screening with Piv-L-*tert*-Leucine as acid. | | | | | | | |  |
| --- | --- | --- | --- | --- | --- | --- | --- | --- |
| **** | | | | | | | | |
| Entry^a^ | Base | Conv. (%) | **2a** (ee) (%) | Ratio (*anti*/*syn*) | **3a** (%) | **4a** (%) | **5a** (%) |  |
| 1 | Lutidine | 58 | 22 (86) | 4 | 6 | - | 14 |  |
| 2 | KOH | - | - | - | - | - | - |  |
| 3 | DBU | - | - | - | - | - | - |  |
| 4 | NaHCO_3_ | 100 | 53 (79) | 6 | - | - | 21 |  |
| 5 | Na_2_CO_3_ | - | - | - | - | - | - |  |

^a^ Reaction conditions as described in *Reaction conditions for epoxidation of naphthalenes,* *workup for the catalysis* and in *epoxide opening* (5.1, 5.2 and 5.3).

| ***Table S13.*** Amino acid screening, catalyst with (*S,S*) chirality. | | | | | | | |
| --- | --- | --- | --- | --- | --- | --- | --- |
| **** | | | | | | | |
| Entry^a^ | Amino acid | Conv. (%) | **2a** (ee) (%) | Ratio (*anti*/*syn*) | **3a** (%) | **4a** (%) | **5a** (%) |
| 1 | L | 100 | 66 (69) | 3 | - | - | 13 |
| 2 | D | 100 | 58 (82) | 3 | - | 2 | 10 |
| 3 |  L | 94 | 50 (67) | 4 | - | - | 17 |
| 4 | L | 100 | 64 (60) | 5 | - | - | 8 |
| 5 | L | 87 | 44 (57) | 4 | - | 3 | 18 |
| 6 | L | 100 | 51 (66) | 3 | - | 4 | 14 |
| 7 | L | 94 | 44 (55) | 5 | - | - | 19 |
| 8 |  | 100 | 49 (68) | 3 | - | - | 25 |

^a^ Reaction conditions as described in *Reaction conditions for epoxidation of naphthalenes,* *workup for the catalysis* and in *epoxide opening* (5.1, 5.2 and 5.3).

| ***Table S14.*** Amino acid screening in presence/absence of base. | | | | | | | | |
| --- | --- | --- | --- | --- | --- | --- | --- | --- |
| **** | | | | | | | | |
| Entry^a^ | Amino acid  (15 mol %) | Base | Conv. (%) | **2a** (ee) (%) | Ratio (*anti*/*syn*) | **3a** (%) | **4a** (%) | **5a** (%) |
| 1 | D | None | 100 | 58 (82) | 3 | - | 2 | 10 |
| 2 | D | 25% Lutidine | 100 | 58 (92) | 2 | - | - | 14 |
| 3 | D | 15% Lutidine | 100 | 54 (94) | 3 | - | - | 11 |
| 4 | None | 25% Lutidine | - | - | - | - | - | - |
| 5 | None | 15% Dipicolinic acid | - | - | - | - | - | - |
| 6 | D | 25% 2,6-Di-tert-butylpyridine | 100 | 58 (88) | 2 | - | - | 10 |
| 7 | D | 15% NaOH | 32 | 1 (84) | 2 | 2 | - | 1 |
| 8 | D | 15% NEt_3_ | 47 | 6 (87) | 2 | 6 | - | 4 |
| 9 | D | 15% Pyridine | 100 | 50 (91) | 2 | - | - | 11 |
| 10 |  | None | 100 | 52 (79) | 2 | - | - | 15 |
| 11 |  | 15% Lutidine | 100 | 57 (82) | 1 | - | - | 14 |
| 12 |  L | None | 100 | 53 (80) | 5 | - | 2 | 22 |
| 13 |  L | 15% Lutidine | 100 | 39 (84) | 2 | - | - | 23 |
| 14 | L | None | 100 | 35 (81) | 2 | - | 1 | 25 |
| 15 | L | 15% Lutidine | 100 | 30 (74) | 2 | - | - | 26 |

^a^ Reaction conditions as described in *Reaction conditions for epoxidation of naphthalenes,* *workup for the catalysis* and in *epoxide opening* (5.1, 5.2 and 5.3).

| ***Table S15.*** Effect of aromatic solvents with using Boc-D-*tert*-Leu as acid. 3 | | | | | | | | ** | |  |
| --- | --- | --- | --- | --- | --- | --- | --- | --- | --- | --- |
|  | | | | | | | | | |  |
| Entry^a^ | | Solvent | Lutidine  (mol%) | Conv.  (%) | **2a**  (ee)  (%) | Ratio  (*anti*/*syn*) | **3a**  (%) | **4a**  (%) | | **5a**  (%) |
| 1 | | CH_3_CN | 0 | 100 | 58 (82) | 3 | - | 2 | | 10 |
| 2 | | CH_3_CN | 15 | 100 | 54 (94) | 3 | - | - | | 11 |
| 3 | | CH_3_CN/ C_6_D_6_ (1:1) | 0 | 93 | 60 (91) | 3 | - | - | | 12 |
| 4 | | CH_3_CN/ C_6_D_6_ (1:1) | 15 | 92 | 46 (93) | 3 | 1.5 | - | | 9 |
| 5 | | Benzonitrile | 0 | 94 | 56 (88) | 3 | - | - | | 9.5 |
| 6 | | Benzonitrile | 15 | 88 | 49 (91) | 3 | - | - | | 9 |
| 7 | | CH_3_CN/ **  (1:1) | 0 | 100 | 58 (88) | 3 | - | - | | 12 |
| 8 | | CH_3_CN/ **  (1:1) | 15 | 94 | 47 (91) | 2 | 3 | - | | 9 |

^a^ Reaction conditions as described in *Reaction conditions for epoxidation of naphthalenes,* *workup for the catalysis* and in *epoxide opening* (5.1, 5.2 and 5.3).

| ***Table S16.*** Effect of aromatic solvents with using Piv-L-*tert*-Leu as acid. | | | | | | | | ** | |
| --- | --- | --- | --- | --- | --- | --- | --- | --- | --- |
|  | | | | | | | | | |
| Entry^a^ | Solvent | Lutidine  (mol%) | Conv.  (%) | **2a**  (ee)  (%) | Ratio  (*anti*/*syn*) | **3a**  (%) | **4a**  (%) | | **5a**  (%) |
| 1 | CH_3_CN | 0 | 100 | 53 (80) | 5 | - | 2 | | 22 |
| 2 | CH_3_CN | 15 | 100 | 39 (84) | 2 | - | - | | 23 |
| 3 | CH_3_CN/ C_6_D_6_ (1:1) | 0 | 100 | 55 (86) | 5 | - | 2 | | 15 |
| 4 | CH_3_CN/ C_6_F_6_ (1:1) | 0 | 100 | 53 (85) | 5 | - | 1.5 | | 18 |
| 5 | Benzonitrile | 0 | 100 | 45 (85) | 4 | - | 2 | | 16 |

^a^ Reaction conditions as described in *Reaction conditions for epoxidation of naphthalenes,* *workup for the catalysis* and in *epoxide opening* (5.1, 5.2 and 5.3).

| ***Table S17.*** Effect of temperature with using Piv-L-*tert*-Leu as acid in aromatic solvents. | | | | | | | | ** | |
| --- | --- | --- | --- | --- | --- | --- | --- | --- | --- |
|  | | | | | | | | | |
| Entry^a^ | Solvent | Temp.  (^o^C) | Conv.  (%) | **2a**  (ee)  (%) | Ratio  (*anti*/*syn*) | **3a**  (%) | **4a**  (%) | | **5a**  (%) |
| 1 | CH_3_CN/ C_6_D_6_ (1:1) | 0 | 100 | 55 (86) | 5 | - | 2 | | 15 |
| 2 | CH_3_CN/ C_6_D_6_ (1:1) | -20 | 94 | 47 (94) | 4 | - | 1 | | 16 |
| 3 | CH_3_CN/ C_6_D_6_ (3:1) | -20 | 89 | 47 (92) | 5 | - | - | | 19 |
| 4 | CH_3_CN/ C_6_D_6_ (3:1) | -40 | 85 | 45 (91) | 4 | - | - | | 18 |
| 5 | CH_3_CN/ C_6_F_6_ (1:1) | -20 | 99 | 46 (87) | 4 | - | 1 | | 18 |
| 6 | CH_3_CN/ ** (1:1) | -20 | 86 | 45 (86) | 4 | - | - | | 18 |

^a^ Reaction conditions as described in *Reaction conditions for epoxidation of naphthalenes,* *workup for the catalysis* and in *epoxide opening* (5.1, 5.2 and 5.3).

| ***Table S18.*** Effect of elongating H_2_O_2_ addition time with using Piv-D-*tert*-Leu as acid in aromatic solvents. | | | | | | | ** | | |
| --- | --- | --- | --- | --- | --- | --- | --- | --- | --- |
|  | | | | | | | | | |
| Entry^a^ | Solvent | H_2_O_2_ Addition Time  (min) | Conv.  (%) | **2a**  (ee)  (%) | Ratio  (*anti*/*syn*) | **3a**  (%) | | **4a**  (%) | **5a**  (%) |
| 1 | CH_3_CN/ C_6_D_6_ (3:1) | 30 | 90 | 49 (92) | 5 | - | | - | 19 |
| 2 | CH_3_CN/ C_6_D_6_ (3:1) | 30+(30 extra reaction time) | 91 | 48 (92) | 5 | - | | - | 16 |
| 3 | CH_3_CN/ C_6_D_6_ (3:1) | 60 | 99 | 62 (91) | 5 | - | | 2 | 26 |

^a^ Reaction conditions as described in *Reaction conditions for epoxidation of naphthalenes,* *workup for the catalysis* and in *epoxide opening* (5.1, 5.2 and 5.3).

| ***Table S19.*** Effect of elongating H_2_O_2_ addition time with using acetic acid in CH_3_CN. | | | | | | | | |
| --- | --- | --- | --- | --- | --- | --- | --- | --- |
|  | | | | | | | | |
| Entry^a^ | Cat. Loading  (mol% of each addition) | H_2_O_2_ Addition Time  (min) | Conv.  (%) | **2a**  (ee)  (%) | Ratio  (*anti*/*syn*) | **3a**  (%) | **4a**  (%) | **5a**  (%) |
| 1 | 1 | 30 | 98 | 66 (44) | 6 | - | 6 | 10 |
| 2 | 1 | 30+(30 extra reaction time) | 99 | 65 (45) | 6 | - | 3 | 9 |
| 3 | 1 | 60 | 90 | 82 (45) | 6 | - | - | 10 |
| 4 | 1.1 | 60 | 99 | 87 (44) | 7 | - | - | 9 |

^a^ Reaction conditions as described in *Reaction conditions for epoxidation of naphthalenes,* *workup for the catalysis* and in *epoxide opening* (5.1, 5.2 and 5.3).

| ***Table S20.*** Competitive reaction. | | | | | | |
| --- | --- | --- | --- | --- | --- | --- |
|  | | | | | | |
| Entry^a^ | R | Conv. 1a  (%) | **2a**  (%) | Ratio  (*anti*/*syn*) | **5a**  (%) | **6q/6r**  (%) |
| 1 | H | 100 | 63 | 3 | - | - |
| 2 | Me | 100 | 49 | 4 | - | - |

Reaction conditions as described in *Reaction conditions for epoxidation of naphthalenes,* *workup for the catalysis* and in *epoxide opening* (5.1 and 5.2).

Analysis of reaction in Entry 1, Table S20

Analysis of reaction in Entry 2, Table S20

# General procedure for isolation of epoxidation products

## Epoxide formation

Conditions A:

In a 15 mL vial equipped with a stir bar and the substrate (0.28 mmol) it was added the Boc-D-*tert-*Leu (42 μmol, 0.15 eq.) and lutidine (42 μmol, 0.15 eq.) in 4 mL of acetonitrile, afterwards the first addition of the (*S,S*)-catalyst (3 mg, 2.8 μmol, 1 mol%) was performed. The mixture was cooled in an ice bath at 0ºC and a solution of H_2_O_2_ 50% aq. (476 μL, 0.59 M, 1 eq.) was added via syringe pump for 10 minutes.

After this, a second and a third addition of catalyst (1 mol%) and H_2_O_2_ (1 eq.) was performed under the same conditions every 10 minutes leading to the total time of the reaction up to 30 minutes.

Conditions B:

In a 15 mL vial equipped with a stir bar and the substrate (94 μmol) it was added the Piv-D-*tert*-Leu (14 μmol, 0.15 eq.) in 783 μL of solvent (acetonitrile:C_6_D_6_, 3:1), afterwards the first addition of the (*S,S*)-catalyst (1 mg, 0.94 μmol, 1 mol%) was performed. The mixture was cooled in an acetonitrile:water (3:1) bath at -20 ^o^C and a solution of H_2_O_2_ 50% aq. (159 μL, 0.59 M, 1 eq.) was added via syringe pump for 20 minutes.

After this, a second and a third addition of catalyst (1 mol%) and H_2_O_2_ (1 eq.) was performed under the same conditions every 20 minutes leading to the total time of the reaction up to 1 hour.

## Workup of the catalysis

After the catalysis was finished, the reaction mixture was filtered through a plug of basic alumina with a very small amount of silica on the top to retain the catalyst and the solvent was removed under vacuum.

## Epoxide opening

Into the rotary evaporated crude of the reaction it was added 285 mg of NaN_3_, 240 mg of NH_4_Cl and 3 mL of MeOH, the reaction was left stirring overnight at 65ºC. The next day, the solvent was removed under vacuum and it was added ethyl acetate and brine (4 mL of each one), the organic phase was dried with MgSO_4_. The solvent was removed under vacuum and the crude of the reaction was purified by flash chromatography silica gel. The purity of the products obtained was checked by ^1^H-NMR, ^13^C-NMR and HRMS-TOF. Diastereomeric ratio was determined by ^1^H-NMR of the reaction crude.

| ***Table S21.*** Substrate scope under Conditions A. | | | | | | | |
| --- | --- | --- | --- | --- | --- | --- | --- |
|  | | | | | | | |
| Entry^a^ | **Substrate 1x** | Conv. (%) | **2x** (%) | Ratio (*anti*/*syn*) | ee *anti* (%) | ee *syn* (%) | **5x** (%) |
| 1 | 1a | 100 | 56 | 3.1 | 94 | meso | 11 |
| 2^b^ | 1b | 100 | 36 | 3.0 | 92 | n.d | 10 |
| 3 | 1c | 100 | 40 | 1.5 | 99 | meso | 14 |
| 4 | 1d | 100 | 39 | 2.1 | 87 | 11 | 1 |
| 5 | 1e | 100 | 49 | 2.7 | 88 | 16 | - |
| 6 | 1f | 100 | 44 | 2.5 | 86 | 2 | - |
| 7 | 1g | 100 | 34 | 2.7 | >99 | 20 | - |
| 8 | 1h | 100 | 51 | 3.7 | >99 | n.d | 11 |
| 9 | 1i | 85 | 46 | 7.2 | >99 | meso | 7 |
| 10 | 1j | 100 | 54 | 2.6 | 76 | 1 | 4 |
| 11 | 1k | 76 | 58 | 2.7 | 86 | 9 | - |
| 12 | 1l | 67 | 32 | 2.6 | 86 | 14 | - |
| 13 | 1m | 100 | 65 | 2.1 | 93 | 86 | 5 |
| 14 | 1n | 100 | 66 | 2.3 | 89 | 85 | 6 |
| 15^b,c^ | 1o | 100 | 36 | 3.0 | 67 | n.d | - |
| 16^b,c^ | 1p | 100 | 40 | 5.0 | 63 | n.d | - |
| 17 | 1q | 54 | 32 | 8.4 | 42 | 71 | - |
| 18 | 1r | 70 | 10 | 6.9 | 68 | 56 | - |
| 19 | 1s | 100 | 32 | 2.2 | 76 | 14 | 2 |

^a^ Reaction conditions as described in *Epoxide formation*, *workup for the catalysis* and in *epoxide opening* (6.1, 6.2 and 6.3).*^b^ Yields and ratios determined by ^1^H-NMR upon integration through the crude of the reaction without purification due to the instability of syn isomer. ^c^ Epoxide formed on the substituted ring.*

| ***Table S22.*** Substrate scope under Conditions B. | | | | | | | |
| --- | --- | --- | --- | --- | --- | --- | --- |
|  | | | | | | | |
| Entry^a^ | **Substrate 1x** | Conv. (%) | **2x** (%) | Ratio (*anti*/*syn*) | ee *anti* (%) | ee *syn* (%) | **5x** (%) |
| 1 | 1a | 99 | 62 | 5.2 | 91 | meso | 26 |
| 2^b^ | 1b | 100 | <5 | n.d | n.d | n.d | n.d |
| 3^b^ | 1c | 100 | 9 | n.d | n.d | meso | n.d |
| 4^b^ | 1d | 100 | <5 | n.d | n.d | n.d | n.d |
| 5 | 1e | 100 | 52 | 2.4 | 74 | 16 | - |
| 6 | 1f | 100 | 59 | 3.3 | 50 | 4 | - |
| 7 | 1g | 100 | 39 | 4.7 | 94 | 18 | - |
| 8 | 1h | 100 | 58 | 5.9 | 96 | 11 | 25 |
| 9 | 1i | 100 | 56 | 10.2 | 99 | meso | 22 |
| 10 | 1j | 95 | 61 | 3.6 | 91 | 15 | 17 |
| 11 | 1k | 100 | 76 | 4.4 | 90 | 7 | 7 |
| 12 | 1l | 59 | 38 | 2.2 | 84 | 14 | 7 |
| 13 | 1m | 100 | 86 | 5.2 | 96 | 5 | 6 |
| 14 | 1n | 100 | 74 | 5 | 94 | 18 | 8 |
| 15^b^ | 1o | 100 | n.d^c^ | - | - | - | n.d |
| 16^b^ | 1p | 100 | <5 | n.d | n.d | n.d | n.d |
| 17 | 1q | 60 | 48 | 5.6 | 64 | 55 | 8 |
| 18 | 1r | 100 | 18 | 5.3 | 68 | 36 | 9 |
| 19 | 1s | 100 | 25 | 3.3 | 80 | 42 | 12 |
| 20 | 1t | 99 | 49 | 3.5 | 94 | 7 | 21 |
| 21 | 1u | 100 | 47 | 2.4 | 88 | - | 22 |
| 22^d^ | 1v | 100 | 33 | - | 76 | - | - |

^a^ Reaction conditions as described in *Epoxide formation*, *workup for the catalysis* and in *epoxide opening* (6.1, 6.2 and 6.3). Conversion, product yields and ratios were determined by ^1^H NMR from crude reaction mixture. *^b^* Reactions were dirty with multiple unidentified products and the desired diepoxides were <5% under Condition B. *^c^* The desired diepoxides were not detected by neither NMR nor HPLC from crude reaction mixture. *^d^* Another 24% of *ipso* addition product was detected, please see *Sections 7 and 9*.

# Characterization of isolated products

**1,2,3,4-tetrahydronaphtho[1,2:3,4]bis(oxirene) (2a)**

Purification by flash chromatography on previously deactivated silica gel with triethyl amine (SiO_2_; Hexane:AcOEt, 9:1) gave the products, as a light yellow solid for both isomers. Chiral SFC analysis was done using Chiralpak IJ-3.

Conditions A: 54% yield (95% ee), 3.1 d.r *anti*/*syn*.

Conditions B: 60% yield (91% ee), 4.9 d.r *anti*/*syn*.

**(1α,2α,3β,4β)-1,2,3,4-tetrahydronaphtho[1,2:3,4]bis(oxirene) (2a*^anti^*)**

^1^H NMR (400 MHz, Chloroform-*d*) δ 7.44 (dd, *J* = 5.5, 3.3 Hz, 2H), 7.35 (dd, *J* = 5.6, 3.3 Hz, 2H), 4.07 – 3.96 (m, 2H), 3.77 – 3.68 (m, 2H).

^13^C NMR (101 MHz, Chloroform-*d*) δ 136.69, 131.66, 129.58, 54.91, 52.11.

HRMS(ESI+) m/z calculated [M+Na]+ 183.0417, found 183.0421.

**(1α,2α,3α,4α)-1,2,3,4-tetrahydronaphtho[1,2:3,4]bis(oxirene) (2a*^syn^*)**

^1^H NMR (400 MHz, Chloroform-*d*) δ 7.66 (dd, *J* = 5.6, 3.3 Hz, 2H), 7.43 (dd, *J* = 5.6, 3.3 Hz, 2H), 4.00 (d, *J* = 2.1 Hz, 2H), 3.92 (dd, *J* = 2.7, 1.2 Hz, 2H).

^13^C NMR (101 MHz, Chloroform-*d*) δ 132.15, 131.38, 129.34, 51.39, 48.04.

HRMS(ESI+) m/z calculated [M+Na]+ 183.0417, found 183.0414.

**1,4-diazido-1,2,3,4-tetrahydronaphthalene-2,3-diol (6e)**

Purification by flash chromatography on silica gel (SiO_2_; Hexane:AcOEt, 7:3) gave the products as a beige solid for *syn* isomer and pale yellow oil for *anti* isomer. Chiral SFC analysis was done using Chiralpak IA-3.

Conditions A: 56% yield (94% ee), 3.1 d.r *anti*/*syn*.

**(1α,2β,3α,4β)-1,4-diazido-1,2,3,4-tetrahydronaphthalene-2,3-diol (6e*^anti^*)**

^1^H NMR (400 MHz, Acetonitrile-*d*_3_) δ 7.48 (dd, *J* = 5.8, 3.5 Hz, 2H), 7.36 (dd, *J* = 5.9, 3.4 Hz, 2H), 4.63 – 4.54 (m, 2H), 4.11 (s, 2H), 3.77 (dd, *J* = 6.5, 2.5 Hz, 2H).

^13^C NMR (101 MHz, Acetonitrile-*d*_3_) δ 133.71, 129.36, 128.71, 75.74, 66.34.

HRMS(ESI+) m/z calculated [M+Na]+ 269.0757, found 269.0762.

[α]D^24^ -20.56 (CHCl_3_, c 0.495).

X-Ray: (CCDC: 2288019)


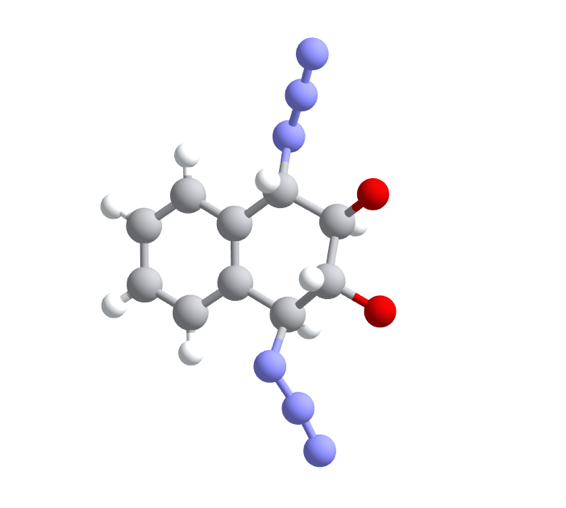


**(1α,2β,3β,4α)-1,4-diazido-1,2,3,4-tetrahydronaphthalene-2,3-diol (6e*^syn^*)**

^1^H NMR (400 MHz, Acetonitrile-*d*_3_) δ 7.50 – 7.45 (m, 2H), 7.44 – 7.40 (m, 2H), 4.68 (d, *J* = 6.0 Hz, 2H), 4.04 (d, *J* = 5.7 Hz, 2H).

^13^C NMR (101 MHz, Acetonitrile-*d*_3_) δ 133.60, 130.21, 129.66, 72.22, 64.03.

HRMS(ESI+) m/z calculated [M+Na]+ 269.0757, found 269.0763.

**(1α,2β,3α,4β)-1,4-diazido-6-ethyl-1,2,3,4-tetrahydronaphthalene-2,3-diol (2b*^anti^*)**

Purification by flash chromatography on silica gel (SiO_2_; Hexane:AcOEt, 8:2) gave the product as a brownish yellow oil. Chiral SFC analysis was done using Chiralpak IG-3.

Conditions A: 24% yield (92% ee).

Conditions B: <5% yield from crude NMR (n.d ee), n.d d.r *anti*/*syn*.

^1^H NMR (400 MHz, Acetonitrile-*d*_3_) δ 7.38 (d, *J* = 8.0 Hz, 1H), 7.32 (s, 1H), 7.22 (dd, *J* = 8.0, 1.1 Hz, 1H), 4.58 – 4.49 (m, 2H), 4.06 (s, 2H), 3.78 – 3.67 (m, 2H), 2.64 (q, *J* = 7.6 Hz, 2H), 1.20 (t, *J* = 7.6 Hz, 3H).

^13^C NMR (101 MHz, Acetonitrile-*d*_3_) δ 145.74, 133.59, 130.98, 129.06, 128.79, 127.92, 75.80, 75.78, 66.42, 66.31, 29.04, 15.86.

HRMS(ESI+) m/z calculated [M+Na]+ 297.1070, found 297.1066.

[α]D^24^ -14.21 (CHCl_3_, c 0.390).

**1,4-diazido-6,7-dimethyl-1,2,3,4-tetrahydronaphthalene-2,3-diol (2c)**

Purification by flash chromatography on silica gel (SiO_2_; Hexane:AcOEt, 8:2) gave the products as a white solid for *syn* isomer and as a beige solid for *anti* isomer. Chiral SFC analysis was done using Chiralpak IG-3.

Conditions A: 40% yield (>99% ee), 1.5 d.r *anti*/*syn*.

Conditions B: 9% yield from crude NMR (n.d ee), n.d d.r *anti*/*syn*.

**(1α,2β,3α,4β)-1,4-diazido-6,7-dimethyl-1,2,3,4-tetrahydronaphthalene-2,3-diol (2c*^anti^*)**

^1^H NMR (400 MHz, Acetonitrile-*d*_3_) δ 7.22 (s, 2H), 4.55 – 4.43 (m, 2H), 4.05 (d, *J* = 2.5 Hz, 2H), 3.70 (d, *J* = 7.4 Hz, 2H), 2.25 (s, 6H).

^13^C NMR (101 MHz, Acetonitrile-*d*_3_) δ 138.18, 130.90, 129.46, 75.77, 66.29, 19.50.

HRMS(ESI+) m/z calculated [M+Na]+ 297.1070, found 297.1070.

[α]D^24^ -10.16 (CHCl_3_, c 0.105).

**(1α,2β,3β,4α)-1,4-diazido-6,7-dimethyl-1,2,3,4-tetrahydronaphthalene-2,3-diol (2c*^syn^*)**

^1^H NMR (400 MHz, Acetonitrile-*d*_3_) δ 7.21 (s, 2H), 4.58 (d, *J* = 6.0 Hz, 2H), 4.05 – 3.95 (m, 2H), 3.62 (s, 2H), 2.27 (s, 6H).

^13^C NMR (101 MHz, Acetonitrile-*d*_3_) δ 138.54, 131.03, 130.81, 72.36, 63.97, 19.54.

HRMS(ESI+) m/z calculated [M+Na]+ 297.1070, found 297.1069.

**1,4-diazido-6-phenyl-1,2,3,4-tetrahydronaphthalene-2,3-diol (2d)**

Purification by flash chromatography on silica gel (SiO_2_; Hexane:AcOEt, 8:2) gave the products as a brown solid for *syn* isomer and as a beige solid for *anti* isomer. Chiral SFC analysis was done using Chiralpak IA-3.

Conditions A: 39% yield (87% ee 2d^anti^ and 11% ee 2d*^syn^*), 2.1 d.r *anti*/*syn*.

Conditions B: <5% yield from crude NMR (n.d ee), n.d d.r *anti*/*syn*.

**(1α,2β,3α,4β)-1,4-diazido-6-phenyl-1,2,3,4-tetrahydronaphthalene-2,3-diol (2d*^anti^*)**

^1^H NMR (400 MHz, Acetonitrile-*d*_3_) δ 7.74 (s, 1H), 7.69 – 7.60 (m, 3H), 7.58 – 7.53 (m, 1H), 7.51 – 7.44 (m, 2H), 7.42 – 7.37 (m, 1H), 4.69 – 4.58 (m, 2H), 4.13 (s, 2H), 3.81 (d, *J* = 8.6 Hz, 2H).

^13^C NMR (101 MHz, Acetonitrile-*d*_3_) δ 141.89, 140.79, 134.32, 132.86, 129.93, 129.42, 128.78, 127.92, 127.86, 127.14, 75.87, 75.80, 66.36, 66.18.

HRMS(ESI+) m/z calculated [M+Na]+ 345.1070, found 345.1068.

[α]D^24^ -20.69 (CHCl_3_, c 0.170).

**(1α,2β,3β,4α)-1,4-diazido-6-phenyl-1,2,3,4-tetrahydronaphthalene-2,3-diol (2d*^syn^*)**

^1^H NMR (400 MHz, Acetonitrile-*d*_3_) δ 7.73 – 7.65 (m, 4H), 7.56 (d, *J* = 8.1 Hz, 1H), 7.52 – 7.46 (m, 2H), 7.43 – 7.38 (m, 1H), 4.75 (dd, *J* = 8.7, 6.2 Hz, 2H), 4.08 (s, 2H), 3.72 (s, 2H).

^13^C NMR (101 MHz, Acetonitrile-*d*_3_) δ 142.20, 140.75, 134.15, 132.81, 130.80, 129.95, 128.68, 128.23, 127.92, 72.34, 72.25, 64.09, 63.84.

HRMS(ESI+) m/z calculated [M+Na]+ 345.1070, found 345.1077.

**1,2,3,4-tetrahydronaphtho[1,2,3,4]bis(oxirene)-7-yl)methyl acetate (2e)**

Purification by flash chromatography on silica gel (SiO_2_; Hexane:AcOEt, 8:2) gave the products as a white solid for *syn* isomer and as a beige solid for *anti* isomer. Chiral SFC analysis was done using Chiralpak IG-3.

Conditions A: 49% yield (88% ee 2e*^anti^* and 16% ee 2e*^syn^*), 2.7 d.r *anti*/*syn*.

Conditions B: 52% yield (74% ee 2e*^anti^* and 16% ee 2e*^syn^*), 2.4 d.r *anti*/*syn*.

**(1α,2α,3β,4β)-1,2,3,4-tetrahydronaphtho[1,2,3,4]bis(oxirene)-7-yl)methyl acetate (2e*^anti^*)**

^1^H NMR (400 MHz, Chloroform-*d*) δ 7.47 – 7.43 (m, 2H), 7.35 (dd, *J* = 7.7, 1.7 Hz, 1H), 4.06 – 4.00 (m, 2H), 3.78 – 3.69 (m, 2H), 2.13 (s, 3H).

^13^C NMR (101 MHz, Chloroform-*d*) δ 170.85, 137.60, 132.14, 131.82, 131.71, 131.39, 129.22, 65.52, 54.89, 54.88, 51.92, 51.75, 21.08.

HRMS(ESI+) m/z calculated [M+Na]+ 255.0628, found 255.0633.

**(1α,2α,3α,4α)-1,2,3,4-tetrahydronaphtho[1,2,3,4]bis(oxirene)-7-yl)methyl acetate (2e*^syn^*)**

^1^H NMR (400 MHz, Chloroform-*d*) δ 7.70 – 7.66 (m, 2H), 7.44 (d, *J* = 7.7 Hz, 1H), 4.02 (s, 2H), 3.96 (dt, *J* = 2.7, 1.5 Hz, 2H), 2.14 (s, 3H).

^13^C NMR (101 MHz, Chloroform-*d*) δ 170.88, 137.39, 132.56, 132.15, 131.63, 131.07, 129.00, 65.62, 51.31, 51.11, 48.08, 48.06, 21.10.

HRMS(ESI+) m/z calculated [M+Na]+ 255.0628, found 255.0627.

**1,2,3,4-tetrahydronaphtho[1,2,3,4]bis(oxirene)-6-yl)acetonitrile (2f)**

Purification by flash chromatography on silica gel (SiO_2_; Hexane:AcOEt, 8:2) gave the products as a white solid for *syn* isomer and as a beige solid for *anti* isomer. Chiral SFC analysis was done using Chiralpak IJ-3 for *anti* isomer and IG-3 for *syn* isomer.

Conditions A: 44% yield (86% ee 2f*^anti^* and 2% ee 2f*^syn^*), 2.5 d.r *anti*/*syn*.

Conditions B: 59% yield (50% ee 2f*^anti^* and 4% ee 2f*^syn^*), 3.3 d.r *anti*/*syn*.

**(1α,2α,3β,4β)-1,2,3,4-tetrahydronaphtho[1,2,3,4]bis(oxirene)-6-yl)acetonitrile (2f*^anti^*)**

^1^H NMR (400 MHz, Chloroform-*d*) δ 7.46 (d, *J* = 7.7 Hz, 1H), 7.42 (s, 1H), 7.32 (dd, *J* = 7.6, 1.7 Hz, 1H), 4.04 – 3.99 (m, 2H), 3.77 (s, 2H), 3.72 (q, *J* = 2.7 Hz, 2H).

^13^C NMR (101 MHz, Chloroform-*d*) δ 133.02, 132.36, 131.90, 131.41, 131.07, 128.96, 117.30, 54.87, 54.85, 51.66, 51.50, 23.46.

HRMS(ESI+) m/z calculated [M+Na]+ 222.0525, found 222.0528.

**(1α,2α,3α,4α)-1,2,3,4-tetrahydronaphtho[1,2,3,4]bis(oxirene)-6-yl)acetonitrile (2f*^syn^*)**

^1^H NMR (400 MHz, Chloroform-*d*) δ 7.69 (d, *J* = 7.8 Hz, 1H), 7.64 (d, *J* = 1.9 Hz, 1H), 7.40 (dd, *J* = 7.9, 2.0 Hz, 1H), 3.99 (d, *J* = 2.6 Hz, 2H), 3.95 (dt, *J* = 2.7, 1.5 Hz, 2H), 3.81 (s, 2H).

^13^C NMR (101 MHz, Chloroform-*d*) δ 133.38, 132.31, 132.20, 131.18, 130.78, 128.78, 117.39, 51.08, 50.89, 48.02, 23.56.

HRMS(ESI+) m/z calculated [M+Na]+ 222.0525, found 222.0519.

**1,2,3,4-tetrahydronaphtho[1,2,3,4]bis(oxirene)-6-yl)methyl acetamide (2g)**

Purification by flash chromatography on silica gel (SiO_2_; Hexane:AcOEt:NH_3_, 7:3:0.1) gave the products as a white solid for *syn* isomer and as a beige solid for *anti* isomer. Chiral SFC analysis was done using Chiralpak IB-3 for *anti* isomer and IJ-3 for *syn* isomer.

Conditions A: 34% yield (>99% ee 2g*^anti^* and 20% ee 2g*^syn^*), 2.7 d.r *anti*/*syn*.

Conditions B: 39% yield (94% ee 2g*^anti^* and 18% ee 2g*^syn^*), 4.7 d.r *anti*/*syn*.

**(1α,2α,3β,4β)-1,2,3,4-tetrahydronaphtho[1,2,3,4]bis(oxirene)-6-yl)methyl acetamide (2g*^anti^*)**

^1^H NMR (400 MHz, Methylene Chloride-*d*_2_) δ 7.39 (d, *J* = 7.6 Hz, 1H), 7.35 (d, *J* = 1.8 Hz, 1H), 7.24 (dd, *J* = 7.7, 1.8 Hz, 1H), 4.38 (dd, *J* = 6.0, 2.9 Hz, 2H), 3.99 – 3.91 (m, 2H), 3.71 – 3.62 (m, 2H), 1.97 (s, 3H).

^13^C NMR (101 MHz, Methylene Chloride-*d*_2_) δ 170.17, 140.77, 132.65, 131.97, 131.28, 131.03, 128.80, 55.14, 55.08, 52.02, 51.81, 43.27, 23.36.

HRMS(ESI+) m/z calculated [M+Na]+ 254.0788, found 254.0793.

**(1α,2α,3α,4α)-1,2,3,4-tetrahydronaphtho[1,2,3,4]bis(oxirene)-6-yl)methyl acetamide (2g*^syn^*)**

^1^H NMR (400 MHz, Methylene Chloride-*d*_2_) δ 7.62 (d, *J* = 7.8 Hz, 1H), 7.57 (d, *J* = 1.9 Hz, 1H), 7.34 (dd, *J* = 7.8, 1.9 Hz, 1H), 4.44 (dd, *J* = 7.7, 6.0 Hz, 2H), 3.94 (dq, *J* = 3.2, 1.7 Hz, 2H), 3.88 (dt, *J* = 2.7, 1.5 Hz, 2H), 2.00 (s, 3H).

^13^C NMR (101 MHz, Methylene Chloride-*d*_2_) δ 169.22, 140.47, 131.76, 131.71, 131.17, 130.57, 128.59, 51.33, 51.11, 48.30, 48.27, 43.37, 23.40.

HRMS(ESI+) m/z calculated [M+Na]+ 254.0788, found 254.0788.

**(4α,5α,6β,7β)-4,5,6,7-tetrahydronaphtho[4,5:6,7]bis(oxirene)-1-yl acetate (2h*^anti^*)**

Purification by crystallization (double layer dichloromethane:pentane) gave the products as white crystals. Chiral SFC analysis was done using Chiralpak IG-3.

Conditions A: 51% yield (>99% ee), 3.7 d.r *anti*/*syn*.

Conditions B: 52% yield (96% ee 2h*^anti^* and 11% ee 2h*^syn^*), 5.9 d.r *anti*/*syn*.

^1^H NMR (400 MHz, Chloroform-*d*) δ 7.44 (d, *J* = 8.2 Hz, 1H), 7.21 (d, *J* = 2.3 Hz, 1H), 7.08 (dd, *J* = 8.2, 2.4 Hz, 1H), 4.02 – 3.96 (m, 2H), 3.74 – 3.70 (m, 1H), 3.70 – 3.66 (m, 1H), 2.30 (s, 3H).

^13^C NMR (101 MHz, Chloroform-*d*) δ 169.10, 151.47, 133.37, 132.68, 129.22, 125.01, 122.49, 54.76, 51.71, 51.52, 21.26.

HRMS(ESI+) m/z calculated [M+Na]+ 241.0471, found 241.0477.

[α]D^24^ +3.08 (CHCl_3_, c 0.105).

**(5α,6α,7β,8β)-5,6,7,8-tetrahydronaphtho[5,6:7,8]bis(oxirene)-1,2-diyl acetate (2i*^anti^*)**

Purification by crystallization (double layer dichloromethane:pentane) gave the products as white crystals. Chiral SFC analysis was done using Chiralpak IG-3.

Conditions A: 46% yield (>99% ee), 7.2 d.r *anti*/*syn*.

Conditions B: 51% yield (>99% ee), 10.2 d.r *anti*/*syn*.

^1^H NMR (400 MHz, Chloroform-*d*) δ 7.29 (s, 2H), 4.01 – 3.95 (m, 2H), 3.71 – 3.63 (m, 2H), 2.29 (s, 6H).

^13^C NMR (101 MHz, Chloroform-*d*) δ 167.93, 142.69, 130.41, 126.79, 54.58, 51.22, 20.75.

HRMS(ESI+) m/z calculated [M+Na]+ 299.0526, found 299.0532.

[α]D^24^ +54.51 (CHCl_3_, c 0.475).

X-Ray: (CCDC: 2298139)


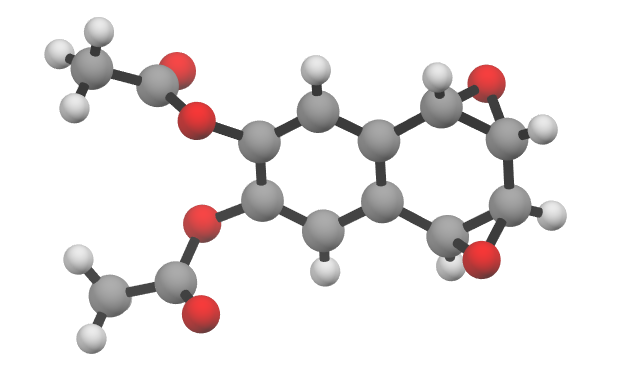


**5,8-diazido-6,7-dihydroxy-5,6,7,8-tetrahydronaphthalen-2-yl)ethan-1-one (2j)**

Purification by flash chromatography on silica gel (SiO_2_; Hexane:AcOEt, 8:2) gave the products as a white solid for *syn* isomer and as a beige oil for *anti* isomer. Chiral SFC analysis was done using Chiralpak IG-3.

Conditions A: 54% yield (76% ee 2j*^anti^* and 1% ee 2j*^syn^*), 2.6 d.r *anti*/*syn*.

Conditions B: 54% yield (91% ee 2j*^anti^* and 15% ee 2j*^syn^*), 3.6 d.r *anti*/*syn*.

**1-((5α,6β,7α,8β)-5,8-diazido-6,7-dihydroxy-5,6,7,8-tetrahydronaphthalen-2-yl)ethan-1-one (2j*^anti^*)**

^1^H NMR (400 MHz, Acetonitrile-*d*_3_) δ 8.04 (s, 1H), 7.91 (ddd, *J* = 8.2, 1.9, 0.8 Hz, 1H), 7.60 (d, *J* = 8.2 Hz, 1H), 4.68 – 4.58 (m, 2H), 4.18 (s, 1H), 3.86 – 3.76 (m, 2H), 2.57 (s, 3H).

^13^C NMR (101 MHz, Acetonitrile-*d*_3_) δ 198.30, 138.56, 138.05, 134.34, 129.24, 128.90, 128.71, 118.26, 75.85, 75.65, 66.02, 65.93, 27.05, 1.89, 1.68.

HRMS(ESI+) m/z calculated [M+Na]+ 311.0863, found 311.0872.

[α]D^24^ -15.20 (CHCl_3_, c 0.390).

**1-((5α,6β,7β,8α)-5,8-diazido-6,7-dihydroxy-5,6,7,8-tetrahydronaphthalen-2-yl)ethan-1-one (2j*^syn^*)**

^1^H NMR (400 MHz, Acetonitrile-*d*_3_) δ 8.04 (d, *J* = 1.9 Hz, 1H), 7.97 (dd, *J* = 8.1, 1.9 Hz, 1H), 7.60 (d, *J* = 8.1 Hz, 1H), 4.76 (dd, *J* = 9.2, 6.1 Hz, 2H), 4.10 – 4.03 (m, 2H), 2.58 (s, 3H).

^13^C NMR (101 MHz, Acetonitrile-*d*_3_) δ 197.25, 137.59, 137.26, 133.15, 129.58, 129.12, 128.24, 71.21, 71.03, 62.70, 62.64, 26.06.

HRMS(ESI+) m/z calculated [M+Na]+ 311.0863, found 311.0862.

**5,8-diazido-6,7-dihydroxy-5,6,7,8-tetrahydronaphthalene-2-carbonitrile (2k)**

Purification by flash chromatography on silica gel (SiO_2_; Hexane:AcOEt, 8:2) gave the products as a beige-pink solid for *syn* isomer and as a beige oil for *anti* isomer. Chiral SFC analysis was done using Chiralpak IA-3.

Conditions A: 58% yield (86% ee 2k*^anti^* and 9% ee 2k*^syn^*), 2.7 d.r *anti*/*syn*.

Conditions B: 72% yield (90% ee 2k*^anti^* and 7% ee 2k*^syn^*), 4.4 d.r *anti*/*syn*.

**(5α,6β,7α,8β)-5,8-diazido-6,7-dihydroxy-5,6,7,8-tetrahydronaphthalene-2-carbonitrile (2k*^anti^*)**

^1^H NMR (400 MHz, Acetonitrile-*d*_3_) δ 7.85 (s, 1H), 7.74 – 7.60 (m, 2H), 4.71 – 4.54 (m, 2H), 3.87 – 3.73 (m, 2H).

^13^C NMR (101 MHz, Acetonitrile-*d*_3_) δ 139.00, 135.39, 132.91, 132.63, 129.93, 119.09, 112.97, 75.60, 75.56, 65.74, 65.42.

HRMS(ESI+) m/z calculated [M+Na]+ 294.0710, found 294.0707.

[α]D^24^ -17.02 (CHCl_3_, c 0.300).

**(5α,6β,7β,8α)-5,8-diazido-6,7-dihydroxy-5,6,7,8-tetrahydronaphthalene-2-carbonitrile (2k*^syn^*)**

^1^H NMR (400 MHz, Acetonitrile-*d*_3_) δ 7.85 (s, 1H), 7.74 (dd, *J* = 8.1, 1.7 Hz, 1H), 7.63 (d, *J* = 8.1 Hz, 1H), 4.74 (dd, *J* = 14.2, 6.3 Hz, 2H), 4.09 – 4.04 (m, 2H).

^13^C NMR (101 MHz, Acetonitrile-*d*_3_) δ 138.97, 135.33, 134.14, 133.00, 131.23, 119.02, 113.27, 71.92, 71.89, 63.38, 63.12.

HRMS(ESI+) m/z calculated [M+Na]+ 294.0710, found 294.0714.

**1-nitro-4,5,6,7-tetrahydronaphtho[4,5:6,7]bis(oxirene) (2l)**

Purification by flash chromatography on previously deactivated silica gel with triethyl amine (SiO_2_; Hexane:AcOEt, 9:1) gave the products as a white solid for both isomers. Chiral SFC analysis was done using Chiralpak IA-3.

Conditions A: 32% yield (86% ee 2l*^anti^* and 14% ee 2l*^syn^*), 2.6 d.r *anti*/*syn*.

Conditions B: 35% yield (84% ee 2l*^anti^* and 14% ee 2l*^syn^*), 2.2 d.r *anti*/*syn*.

**(4α,5α,6β,7β)-1-nitro-4,5,6,7-tetrahydronaphtho[4,5:6,7]bis(oxirene) (2l*^anti^*)**

^1^H NMR (400 MHz, Chloroform-*d*) δ 8.30 (d, *J* = 2.3 Hz, 1H), 8.21 (dd, *J* = 8.3, 2.3 Hz, 1H), 7.62 (d, *J* = 8.3 Hz, 1H), 4.11 – 4.02 (m, 2H), 3.80 (dd, *J* = 10.6, 3.9 Hz, 2H).

^13^C NMR (101 MHz, Chloroform-*d*) δ 148.54, 139.03, 134.03, 132.53, 126.35, 124.46, 55.30, 54.84, 51.15, 50.97.

HRMS(ESI+) m/z calculated [M+Na]+ 228.0267, found 228.0271.

[α]D^24^ +16.29 (CHCl_3_, c 0.070).

**(4α,5α,6α,7α)-1-nitro-4,5,6,7-tetrahydronaphtho[4,5:6,7]bis(oxirene) (2l*^syn^*)**

^1^H NMR (400 MHz, Chloroform-*d*) δ 8.57 (d, *J* = 2.3 Hz, 1H), 8.31 (dd, *J* = 8.3, 2.4 Hz, 1H), 7.89 (d, *J* = 8.3 Hz, 1H), 4.13 (d, *J* = 3.3 Hz, 1H), 4.10 (d, *J* = 3.3 Hz, 1H), 4.04 (p, *J* = 2.9 Hz, 2H).

^13^C NMR (101 MHz, Chloroform-*d*) δ 139.23, 134.25, 132.52, 126.23, 123.96, 50.84, 50.46, 48.23, 47.86.

HRMS(ESI+) m/z calculated [M+Na]+ 228.0267, found 228.0265.

[α]D^24^ +14.07 (CHCl_3_, c 0.150).

**1,4-diazido-6-bromo-1,2,3,4-tetrahydronaphthalene-2,3-diol (2m)**

Purification by flash chromatography on silica gel (SiO_2_; Hexane:AcOEt, 8:2) gave the products as a white solid for *syn* isomer and as a beige solid for *anti* isomer. Chiral SFC analysis was done using Chiralpak IA-3.

Conditions A: 65% yield (93% ee 2m*^anti^* and 86% ee 2m*^syn^*), 2.1 d.r *anti*/*syn*.

Conditions B: 83% yield (96% ee 2m*^anti^* and 5% ee 2m*^syn^*), 5.2 d.r *anti*/*syn*.

**(1α,2β,3α,4β)-1,4-diazido-6-bromo-1,2,3,4-tetrahydronaphthalene-2,3-diol (2m*^anti^*)**

^1^H NMR (400 MHz, Acetonitrile-*d*_3_) δ 7.65 (dd, *J* = 2.1, 1.1 Hz, 1H), 7.51 (dd, *J* = 8.5, 2.1 Hz, 1H), 7.39 (dd, *J* = 8.4, 1.0 Hz, 1H), 4.55 (dd, *J* = 17.2, 8.3 Hz, 2H), 4.13 (s, 2H), 3.76 (dd, *J* = 6.5, 2.5 Hz, 2H).

^13^C NMR (101 MHz, Acetonitrile-*d*_3_) δ 136.20, 133.14, 132.35, 131.53, 130.88, 122.69, 75.70, 75.66, 65.78, 65.62.

HRMS(ESI+) m/z calculated [M+Na]+ 346.9863 and 348.9842, found 346.9870 and 348.9855.

[α]D^24^ -20.84 (CHCl_3_, c 0.360).

**(1α,2β,3β,4α)-1,4-diazido-6-bromo-1,2,3,4-tetrahydronaphthalene-2,3-diol (2m*^syn^*)**

^1^H NMR (400 MHz, Acetonitrile-*d*_3_) δ 7.66 (d, *J* = 1.9 Hz, 1H), 7.56 (dd, *J* = 8.2, 2.1 Hz, 1H), 7.38 (d, *J* = 8.3 Hz, 1H), 4.66 (t, *J* = 5.7 Hz, 2H), 4.03 (t, *J* = 5.0 Hz, 2H), 3.73 (t, *J* = 3.9 Hz, 2H).

^13^C NMR (101 MHz, Acetonitrile-*d*_3_) δ 136.26, 132.91, 132.90, 132.64, 132.25, 123.00, 72.11, 72.03, 63.45, 63.30.

HRMS(ESI+) m/z calculated [M+Na]+ 346.9863 and 348.9842, found 346.9891 and 348.9890.

[α]D^24^ -12.27 (CHCl_3_, c 0.250).

**1,4-diazido-6-chloro-1,2,3,4-tetrahydronaphthalene-2,3-diol (2n)**

Purification by flash chromatography on silica gel (SiO_2_; Hexane:AcOEt, 8:2) gave the products as a brown solid for *syn* isomer and as a beige solid for *anti* isomer. Chiral SFC analysis was done using Chiralpak IA-3.

Conditions A: 66% yield (89% ee 2n*^anti^* and 85% ee 2n*^syn^*), 2.3 d.r *anti*/*syn*.

Conditions B: 72% yield (94% ee 2n*^anti^* and 18% ee 2n*^syn^*), 5.0 d.r *anti*/*syn*.

**(1α,2β,3α,4β)-1,4-diazido-6-chloro-1,2,3,4-tetrahydronaphthalene-2,3-diol (2n*^anti^*)**

^1^H NMR (400 MHz, Acetonitrile-*d*_3_) δ 7.52 – 7.42 (m, 2H), 7.36 (dd, *J* = 8.2, 2.0 Hz, 1H), 4.61 – 4.51 (m, 2H), 4.16 (s, 2H), 3.76 (dd, *J* = 6.5, 2.5 Hz, 2H).

^13^C NMR (101 MHz, Acetonitrile-*d*_3_) δ 135.96, 134.64, 132.63, 130.67, 129.40, 128.49, 75.69, 65.73, 65.66.

HRMS(ESI+) m/z calculated [M+Na]+ 303.0368, found 303.0366.

[α]D^24^ -22.22 (CHCl_3_, c 0.525).

**(1α,2β,3β,4α)-1,4-diazido-6-chloro-1,2,3,4-tetrahydronaphthalene-2,3-diol (2n*^syn^*)**

^1^H NMR (400 MHz, Acetonitrile-*d*_3_) δ 7.51 (d, *J* = 2.1 Hz, 1H), 7.47 – 7.39 (m, 2H), 4.67 (dd, *J* = 6.1, 2.6 Hz, 2H), 4.03 (t, *J* = 4.6 Hz, 2H), 3.74 (s, 2H).

^13^C NMR (101 MHz, Acetonitrile-*d*_3_) δ 136.05, 134.96, 132.43, 132.05, 129.87, 129.68, 72.16, 72.03, 63.40, 63.36.

HRMS(ESI+) m/z calculated [M+Na]+ 303.0368, found 303.0362.

[α]D^24^ -17.84 (CHCl_3_, c 0.170).

**(1α,2β,3α,4β)-1,4-diazido-1-ethyl-1,2,3,4-tetrahydronaphthalene-2,3-diol (2o*^anti^*)**

Purification by flash chromatography on silica gel (SiO_2_; Hexane:AcOEt, 8:2) gave the products as a brown oil. Chiral SFC analysis was done using Chiralpak IG-3.

Conditions A: 25% yield (67% ee).

Conditions B: n.d.

^1^H NMR (400 MHz, Acetonitrile-*d*_3_) δ 7.55 – 7.51 (m, 1H), 7.51 – 7.46 (m, 1H), 7.45 – 7.38 (m, 2H), 4.62 (d, *J* = 7.7 Hz, 1H), 4.10 – 3.90 (m, 4H), 2.33 – 2.22 (m, 1H), 1.64 (dq, *J* = 14.7, 7.4 Hz, 1H), 0.86 (t, *J* = 7.5 Hz, 3H).

^13^C NMR (101 MHz, Acetonitrile-*d*_3_) δ 136.90, 133.28, 129.44, 129.34, 129.03, 128.83, 77.00, 74.25, 70.59, 66.95, 27.98, 8.60.

HRMS(ESI+) m/z calculated [M+Na]+ 297.1070, found 297.1075.

[α]D^24^ -15.77 (CHCl_3_, c 0.190).

**(1α,2β,3α,4β)-1,4-diazido-1-methyl-1,2,3,4-tetrahydronaphthalene-2,3-diol (2q*^anti^*)**

Purification by flash chromatography on silica gel (SiO_2_; Hexane:AcOEt, 8:2) gave the products as a brown oil. Chiral SFC analysis was done using Chiralpak IG-3.

Conditions A: 33% yield (63% ee).

Conditions B: <5% yield (n.d ee).

^1^H NMR (400 MHz, Acetonitrile-*d*_3_) δ 7.54 – 7.43 (m, 2H), 7.43 – 7.32 (m, 2H), 4.60 (d, *J* = 8.5 Hz, 1H), 4.03 (s, 2H), 3.94 – 3.79 (m, 2H), 1.48 (s, 3H).

^13^C NMR (101 MHz, Acetonitrile-*d*_3_) δ 139.66, 132.62, 129.62, 129.27, 128.81, 128.17, 76.19, 74.13, 67.94, 66.66, 23.22.

HRMS(ESI+) m/z calculated [M+Na]+ 283.0914, found 283.0912.

[α]D^24^ +20.75 (CHCl_3_, c 0.285).

**5,8-diazido-6,7-dihydroxy-5,6,7,8-tetrahydronaphthalene-1-carbonitrile (2q)**

Purification by flash chromatography on silica gel (SiO_2_; Hexane:AcOEt, 8:2) gave the products as a beige solid for *syn* isomer and as a yellow oil for *anti* isomer. Chiral SFC analysis was done using Chiralpak IG-3.

Conditions A: 32% yield (42% ee 2q*^anti^* and 71% ee 2q*^syn^*), 8.4 d.r *anti*/*syn*.

Conditions B: 49% yield (64% ee 2q*^anti^* and 55% ee 2q*^syn^*), 5.6 d.r *anti*/*syn*.

**(5α,6β,7α,8β)-5,8-diazido-6,7-dihydroxy-5,6,7,8-tetrahydronaphthalene-1-carbonitrile (2q*^anti^*)**

^1^H NMR (400 MHz, Acetonitrile-*d*_3_) δ 7.80 (ddt, *J* = 7.6, 2.0, 1.1 Hz, 2H), 7.54 (td, *J* = 7.8, 0.7 Hz, 1H), 4.80 (d, *J* = 8.2 Hz, 1H), 4.65 (dq, *J* = 9.4, 1.2 Hz, 1H), 3.88 (dd, *J* = 10.1, 8.2 Hz, 1H), 3.72 (dd, *J* = 10.1, 9.4 Hz, 1H).

^13^C NMR (101 MHz, Acetonitrile-*d*_3_) δ 137.26, 136.33, 135.30, 133.29, 130.03, 113.62, 76.05, 74.99, 66.47, 65.22.

HRMS(ESI+) m/z calculated [M+Na]+ 294.0710, found 294.0714.

[α]D^24^ +32.11 (CHCl_3_, c 0.280).

**(5α,6β,7β,8α)-5,8-diazido-6,7-dihydroxy-5,6,7,8-tetrahydronaphthalene-1-carbonitrile (2q*^syn^*)**

^1^H NMR (400 MHz, Acetonitrile-*d*_3_) δ 7.85 – 7.77 (m, 2H), 7.57 (t, *J* = 7.8 Hz, 1H), 4.93 (d, *J* = 4.4 Hz, 1H), 4.73 (d, *J* = 8.0 Hz, 1H), 4.22 (dd, *J* = 4.5, 2.1 Hz, 1H), 4.05 (dd, *J* = 8.1, 2.2 Hz, 1H).

^13^C NMR (101 MHz, Acetonitrile-*d*_3_) δ 136.60, 136.39, 134.71, 134.60, 130.44, 114.64, 72.45, 71.38, 63.11, 63.08.

HRMS(ESI+) m/z calculated [M+Na]+ 294.0710, found 294.0705.

[α]D^24^ +29.81 (CHCl_3_, c 0.070).

**1,4-diazido-5-bromo-1,2,3,4-tetrahydronaphthalene-2,3-diol (2r)**

Purification by flash chromatography on silica gel (SiO_2_; Hexane:AcOEt, 8.5:1.5) gave the products as a brown oil for both isomers. Chiral SFC analysis was done using Chiralpak IG-3.

Conditions A: 10% yield (68% ee 2r*^anti^* and 56% ee 2r*^syn^*), 6.9 d.r *anti*/*syn*.

Conditions B: 16% yield (68% ee 2r*^anti^* and 36% ee 2r*^syn^*), 5.3 d.r *anti*/*syn*.

**(1α,2β,3α,4β)-1,4-diazido-5-bromo-1,2,3,4-tetrahydronaphthalene-2,3-diol (2r*^anti^*)**

^1^H NMR (400 MHz, Acetonitrile-*d*_3_) δ 7.70 – 7.61 (m, 1H), 7.61 – 7.50 (m, 1H), 7.38 – 7.26 (m, 1H), 4.71 (d, *J* = 5.8 Hz, 1H), 4.62 (d, *J* = 9.1 Hz, 1H), 4.11 (s, 1H), 4.05 – 3.92 (m, 2H), 3.60 (t, *J* = 8.7 Hz, 1H).

^13^C NMR (101 MHz, Acetonitrile-*d*_3_) δ 138.05, 134.09, 132.19, 131.24, 127.67, 125.69, 76.52, 75.69, 67.74, 64.92.

HRMS(ESI+) m/z calculated [M+Na]+ 346.9863 and 348.9842, found 346.9862 and 348.9844.

**(1α,2β,3β,4α)-1,4-diazido-5-bromo-1,2,3,4-tetrahydronaphthalene-2,3-diol (2r*^syn^*)**

^1^H NMR (400 MHz, Acetonitrile-*d*_3_) δ 7.70 – 7.61 (m, 3H), 7.61 – 7.50 (m, 3H), 7.38 – 7.26 (m, 3H), 4.89 (d, *J* = 3.6 Hz, 1H), 4.68 (d, *J* = 9.1 Hz, 1H), 4.21 – 4.18 (m, 2H), 4.05 – 3.92 (m, 5H), 3.80 – 3.67 (m, 2H).

^13^C NMR (101 MHz, Acetonitrile-*d*_3_) δ 138.39, 133.84, 131.48, 130.89, 129.16, 127.00, 72.94, 71.24, 64.51, 63.43.

HRMS(ESI+) m/z calculated [M+Na]+ 346.9863 and 348.9842, found 346.9862 and 348.9844.

**1,4-diazido-5-fluoro-1,2,3,4-tetrahydronaphthalene-2,3-diol (2s)**

Purification by flash chromatography on silica gel (SiO_2_; Hexane:AcOEt, 8:2) gave the products as a beige solid for both isomers, as a white solid for *syn* isomer and as a beige solid for *anti* isomer. Chiral SFC analysis was done using Chiralpak IG-3.

Conditions A: 32% yield (76% ee 2s*^anti^* and 14% ee 2s*^syn^*), 2.2 d.r *anti*/*syn*.

Conditions B: 19% yield (80% ee 2s*^anti^* and 42% ee 2s*^syn^*), 3.3 d.r *anti*/*syn*.

**(1α,2β,3α,4β)-1,4-diazido-5-fluoro-1,2,3,4-tetrahydronaphthalene-2,3-diol (2s*^anti^*)**

^1^H NMR (400 MHz, Acetonitrile-*d*_3_) δ 7.46 – 7.39 (m, 1H), 7.34 (dt, *J* = 8.1, 1.3 Hz, 1H), 7.13 (ddt, *J* = 10.5, 8.2, 1.1 Hz, 1H), 4.70 (d, *J* = 7.7 Hz, 1H), 4.58 (dt, *J* = 9.3, 1.1 Hz, 1H), 4.09 (s, 2H), 3.78 (dd, *J* = 9.9, 7.8 Hz, 1H), 3.63 (t, *J* = 9.6 Hz, 1H).

^13^C NMR (101 MHz, Acetonitrile-*d*_3_) δ 163.57, 161.10, 137.23 (d), 131.45 (d), 124.20 (d), 121.20 (d), 116.14 (d), 75.74, 75.12, 65.14 (d), 64.28.

^19^F NMR (377 MHz, Acetonitrile-*d*_3_) δ -115.77.

HRMS(ESI+) m/z calculated [M+Na]+ 287.0663, found 287.0660.

[α]D^24^ 50.56 (CHCl_3_, c 0.185).

**(1α,2β,3β,4α)-1,4-diazido-5-fluoro-1,2,3,4-tetrahydronaphthalene-2,3-diol (2s*^syn^*)**

^1^H NMR (400 MHz, Acetonitrile-*d*_3_) δ 7.47 (td, *J* = 8.0, 5.7 Hz, 1H), 7.38 (dt, *J* = 8.0, 1.2 Hz, 1H), 7.17 (ddt, *J* = 10.1, 8.1, 1.0 Hz, 1H), 4.92 (dd, *J* = 3.9, 1.5 Hz, 1H), 4.68 (d, *J* = 8.6 Hz, 1H), 4.14 – 4.07 (m, 1H), 3.98 (ddd, *J* = 8.4, 5.6, 2.1 Hz, 1H), 3.72 (dd, *J* = 13.1, 5.1 Hz, 2H).

^13^C NMR (101 MHz, Acetonitrile-*d*_3_) δ 163.81, 161.37, 137.77 (d), 131.73 (d), 125.45 (d), 120.33(d), 115.83 (d), 72.33, 71.35, 63.14 (d), 59.30 (d).

^19^F NMR (377 MHz, Acetonitrile-*d*_3_) δ -119.04.

HRMS(ESI+) m/z calculated [M+Na]+ 287.0663, found 287.0660.

[α]D^24^ +15.04 (CHCl_3_, c 0.150).

**2-methyl-5,6,7,8-tetrahydronaphtho[5,6:7,8]bis(oxirene)-1-yl acetate (2t)**

Purification by flash chromatography on silica gel (SiO_2_; Hexane:AcOEt, 9:1) gave the products as a beige solid for both isomers. Chiral SFC analysis was done using Chiralpak IG-3.

Conditions B: 31% yield for 2t*^anti^* (94% ee 2t*^anti^* and 7% ee 2t*^syn^*), 3.5 d.r *anti*/*syn*.

**(5α,6α,7β,8β)-2-methyl-5,6,7,8-tetrahydronaphtho[5,6:7,8]bis(oxirene)-1-yl acetate (2t*^anti^*)**

^1^H NMR (400 MHz, Chloroform-d) δ 7.30 (s, 1H), 7.12 (s, 1H), 4.02 – 3.90 (m, 2H), 3.67 (dd, J = 7.1, 4.3 Hz, 2H), 2.32 (s, 3H), 2.18 (s, 3H).

^13^C NMR (101 MHz, Chloroform-d) δ 168.92, 135.14, 134.48, 130.65, 129.28, 125.36, 109.36, 54.72, 54.61, 51.59, 51.58, 20.92, 16.10.

HRMS(ESI+) m/z calculated [M+H]+ 233.0808, found 233.0765.

X-Ray: (CCDC: 2359644).


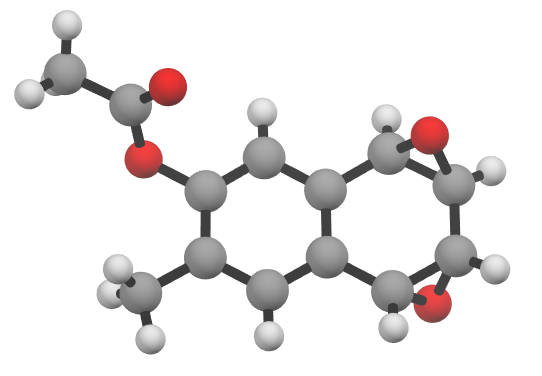


**(4α,5α,6β,7β)-2-methyl-4,5,6,7,-tetrahydronaphtho[4,5:6,7]bis(oxirene)-1-yl acetate (2u*^anti^*)**

Purification by flash chromatography on silica gel (SiO_2_; Hexane:AcOEt, 9:1) gave the products as a beige solid. Chiral SFC analysis was done using Chiralpak IB-3.

Conditions B: 28% yield for 2u*^anti^* (88% ee 2u*^anti^* and n.d ee 2u*^syn^*), 2.4 d.r *anti*/*syn*.

^1^H NMR (400 MHz, Chloroform-*d*) δ 7.29 (d, *J* = 8.1 Hz, 1H), 7.02 (d, *J* = 8.1 Hz, 1H), 4.01 – 3.95 (m, 2H), 3.89 (d, *J* = 4.7 Hz, 1H), 3.72 – 3.69 (m, 1H), 2.33 (s, 3H), 2.27 (s, 3H).

^13^C NMR (101 MHz, Chloroform-*d*) δ 169.03, 150.42, 132.62, 131.48, 130.07, 129.49, 122.94, 54.79, 54.02, 52.10, 48.56, 20.94, 11.71.

HRMS(ESI+) m/z calculated [M+H]+ 233.0808, found 233.0811.

**1,4-diazido-6-bromo-5-methyl-1,2,3,4-tetrahydronaphthalene-2,3-diol (2v)**

Purification by flash chromatography on silica gel (SiO_2_; Hexane:AcOEt, 8:2) gave the products as a beige solid. Chiral SFC analysis was done using Chiralpak IA-3.

Conditions B: 26% yield for 2v*^anti^* (76% ee 2v*^anti^*), 16% yield for 2v.1.

**1,4-diazido-6-bromo-5-methyl-1,2,3,4-tetrahydronaphthalene-2,3-diol (2v.1)**

+

HRMS(ESI-) m/z calculated [M-H]- 337.0075, found 337.0054.

**(1α,2β,3α,4β)-1,4-diazido-6-bromo-5-methyl-1,2,3,4-tetrahydronaphthalene-2,3-diol (2v*^anti^*)**

^1^H NMR (400 MHz, Acetonitrile-*d*_3_) δ 7.78 (d, *J* = 8.4 Hz, 1H), 7.44 (d, *J* = 8.5 Hz, 1H), 4.84 (d, *J* = 6.6 Hz, 1H), 4.71 (d, *J* = 9.1 Hz, 1H), 4.22 (d, *J* = 19.1 Hz, 1H), 4.12 (dd, *J* = 9.1, 6.7 Hz, 1H), 3.75 (t, *J* = 9.1 Hz, 1H), 2.63 (s, 3H).

^13^C NMR (101 MHz, Acetonitrile-*d*_3_) δ 136.80, 133.39, 131.94, 131.70, 125.66, 124.76, 74.40, 73.27, 64.50, 63.26, 18.95.

HRMS(ESI-) m/z calculated [M-H]- 337.0063, found 337.0054.

# Product derivatization

Product derivatizations were made following the procedures reported.^11^

**1,2,3,4-tetrahydronaphthalene-2,3-diol (6a)**

Starting with 10 mg of 1a, 5.3 mg of product were obtained as white solid (82% yield, >99% ee). Chiral SFC analysis was done using Chiralpak IA-3.

^1^H NMR (400 MHz, Chloroform-*d*) δ 7.19 – 7.06 (m, 4H), 3.89 (dd, *J* = 9.0, 4.6 Hz, 2H), 3.27 – 3.16 (m, 2H), 2.94 – 2.77 (m, 2H), 2.44 – 2.34 (m, 2H).

^13^C NMR (101 MHz, Chloroform-*d*) δ 133.93, 128.99, 126.47, 72.45, 36.96.

ESI-MS(H^+^) m/z [M+Na^+^] 187.0.

**1,2,3,4-tetrahydronaphthalene-1,2,3,4-tetraol (6b)**

Starting with 10 mg of 1a, 7.4 mg of product were obtained as white solid (60% yield, 91% ee). Chiral SFC analysis was done using Chiralpak IC-3.

^1^H NMR (400 MHz, Chloroform-*d*) δ 7.53 (dt, *J* = 7.2, 1.1 Hz, 1H), 7.47 – 7.36 (m, 3H), 4.60 (s, 1H), 4.54 (d, *J* = 11.0 Hz, 1H), 4.06 (d, *J* = 3.9 Hz, 1H), 3.93 (ddd, *J* = 3.8, 2.9, 2.0 Hz, 1H), 2.73 (d, *J* = 11.9 Hz, 1H), 1.80 (s, 1H), 1.66 (s, 2H).

^13^C NMR (101 MHz, Chloroform-*d*) δ 135.51, 131.91, 131.82, 130.11, 128.98, 72.75, 67.02, 57.04, 53.12, 28.15, 27.26.

ESI-MS m/z [M] 196.0.

**3-methoxy-1,2,3,7-tetrahydronaphtho[1,2]oxiren-2-ol (6c)**

Starting with 10 mg of 1a, 9.9 mg of product were obtained as white solid (82% yield, 91% ee). Chiral SFC analysis was done using Chiralpak IG-3.

^1^H NMR (400 MHz, Chloroform-*d*) δ 7.57 – 7.50 (m, 1H), 7.43 – 7.36 (m, 2H), 7.36 – 7.32 (m, 1H), 4.53 (dt, *J* = 7.4, 2.8 Hz, 1H), 4.27 (dd, *J* = 3.2, 1.6 Hz, 1H), 3.93 (d, *J* = 3.8 Hz, 1H), 3.79 (ddd, *J* = 3.9, 2.5, 1.6 Hz, 1H), 3.42 (s, 3H), 1.73 (d, *J* = 7.5 Hz, 1H).

^13^C NMR (101 MHz, Chloroform-*d*) δ 132.97, 132.23, 131.65, 130.67, 129.15, 129.01, 81.84, 67.17, 57.76, 56.30, 51.29.

ESI-MS(H^+^) m/z [M+Na^+^] 215.0.

**1,4-bis(dimethylamino)-1,2,3,4-tetrahydronaphthalene-2,3-diol (6d)**

Starting with 10 mg of 1a, 9.4 mg of product were obtained as white solid (96% yield, >99% ee). Chiral SFC analysis was done using Chiralpak IG-3.

^1^H NMR (400 MHz, Chloroform-*d*) δ 7.46 (dd, *J* = 5.7, 3.5 Hz, 2H), 7.27 (s, 1H), 7.25 (d, *J* = 3.3 Hz, 1H), 3.94 (dd, *J* = 5.4, 2.4 Hz, 2H), 3.76 (dd, *J* = 5.3, 2.4 Hz, 2H), 2.47 (s, 12H).

^13^C NMR (101 MHz, Chloroform-*d*) δ 135.56, 129.09, 127.34, 70.96, 68.60, 42.13.

ESI-MS(H^+^) m/z [M+H^+^] 251.1.

**1,4-dimethoxy-1,2,3,4-tetrahydronaphthalene-2,3-diol (6i)**

Starting with 10 mg of 1a, 7.5 mg of product were obtained as white solid (71% yield, >99% ee). Chiral SFC analysis was done using Chiralpak IA-3.

^1^H NMR (400 MHz, Chloroform-*d*) δ 7.44 (dd, *J* = 5.6, 3.5 Hz, 1H), 7.39 – 7.33 (m, 1H), 4.46 – 4.39 (m, 1H), 4.04 – 3.97 (m, 1H), 3.58 (s, 3H), 3.12 – 3.07 (m, 1H).

^13^C NMR (101 MHz, Chloroform-*d*) δ 134.05, 128.62, 128.19, 81.81, 72.92, 58.02.

ESI-MS(Na^+^) m/z [M+Na^+^] 247.1.

**1,4-bis(*p*-tolyloxy)-1,2,3,4-tetrahydronaphthalene-2,3-diol (6h)**

Starting with 10 mg of 1a, 13 mg of product were obtained as white solid (55% yield, 92% ee). Chiral SFC analysis was done using Chiralpak IC-3.

^1^H NMR (400 MHz, Chloroform-*d*) δ 7.42 (dd, *J* = 5.7, 3.5 Hz, 2H), 7.39 – 7.29 (m, 2H), 7.16 – 7.11 (m, 4H), 7.04 – 6.97 (m, 4H), 5.45 (d, *J* = 5.2 Hz, 2H), 4.45 (d, *J* = 5.1 Hz, 2H), 2.64 (d, *J* = 1.9 Hz, 2H), 2.32 (s, 6H).

^13^C NMR (101 MHz, Chloroform-*d*) δ 157.01, 134.12, 131.27, 130.38, 128.69, 128.30, 115.92, 70.99, 20.67.

HRMS(ESI+) m/z calculated [M-H]+ 375.1602, found 375.1605.

**1,4-dimethyl-1,2,3,4-tetrahydronaphthalene-2,3-diol (6g)**

Starting with 10 mg of 1a, 5.3 mg of product were obtained as white solid (44% yield, 91% ee). Chiral SFC analysis was done using Chiralpak IG-3.

^1^H NMR (400 MHz, Chloroform-*d*) δ 7.32 – 7.27 (m, 2H), 7.24 – 7.18 (m, 2H), 3.47 – 3.38 (m, 2H), 2.85 (dq, *J* = 9.2, 6.6 Hz, 2H), 2.53 (s, 2H), 1.49 (s, 3H), 1.48 (s, 3H).

^13^C NMR (101 MHz, Chloroform-*d*) δ 138.57, 126.99, 126.67, 77.65, 40.06, 17.70.

ESI-MS(Na^+^) m/z [M+Na^+^] 215.0.

**1,4-bis(butylthiol)-1,2,3,4-tetrahydronaphthalene-2,3-diol (6f)**

Starting with 10 mg of 1a, 3.3 mg of product were obtained as white solid (76% yield, 91% ee). Chiral SFC analysis was done using Chiralpak IB-3.

^1^H NMR (400 MHz, Chloroform-*d*) δ 7.86 – 7.80 (m, 1H), 7.62 – 7.57 (m, 1H), 7.31 (dd, *J* = 5.9, 3.4 Hz, 1H), 7.26 (dd, *J* = 5.8, 3.4 Hz, 1H), 4.46 – 4.42 (m, 1H), 4.06 (d, *J* = 5.9 Hz, 1H), 3.91 (dt, *J* = 7.8, 3.9 Hz, 1H), 3.84 – 3.77 (m, 1H), 2.62 (dt, *J* = 12.2, 7.3 Hz, 1H), 2.55 – 2.45 (m, 2H), 2.36 (dt, *J* = 11.9, 7.5 Hz, 1H), 1.65 – 1.55 (m, 5H), 1.49 – 1.35 (m, 4H), 0.91 (dt, *J* = 11.3, 7.3 Hz, 6H).

^13^C NMR (101 MHz, Chloroform-*d*) δ 134.67, 134.27, 130.01, 129.42, 127.84, 127.63, 73.90, 69.69, 52.14, 48.78, 31.81, 31.67, 30.40, 28.96, 28.16, 27.30, 22.21, 13.81, 13.80.

HRMS(ESI+) m/z calculated [M+Na]+ 363.1423, found 363.1425.

# NMR

**(1α,2α,3β,4β)-1,2,3,4-tetrahydronaphtho[1,2:3,4]bis(oxirene) (2a*^anti^*)**

**2a*^anti^*** - ^1^H NMR (400 MHz, CDCl_3_)

**2a*^anti^*** -^13^C NMR (100 MHz, CDCl_3_)

**2a*^anti^*** -^1^H-^1^H COSY (CDCl_3_)

**(1α,2α,3α,4α)-1,2,3,4-tetrahydronaphtho[1,2:3,4]bis(oxirene) (2a*^syn^*)**

**2a*^syn^*** - ^1^H NMR (400 MHz, CDCl_3_)

**2a*^syn^*** -^13^C NMR (100 MHz, CDCl_3_)

**2a*^syn^*** -DEPT (CDCl_3_)

**2a*^syn^*** -DEPTQ (CDCl_3_)

**2a*^syn^*** -^1^H-^1^H COSY (CDCl_3_)

**2a*^syn^*** -^1^H-^13^C HSQCED (CDCl_3_)

**2a*^syn^*** -^1^H-^13^C HMBC (CDCl_3_)

**(1α,2α,3β,4β)-1,2,3,4-tetrahydronaphtho[1,2,3,4]bis(oxirene)-7-yl)methyl acetate (2e*^anti^*)**

**2e*^anti^*** - ^1^H NMR (400 MHz, CDCl_3_)

**2e*^anti^*** -^13^C NMR (100 MHz, CDCl_3_)

**2e*^anti^*** -DEPT (CDCl_3_)

**2e*^anti^*** -DEPTQ (CDCl_3_)

**2e*^anti^*** -^1^H-^1^H COSY (CDCl_3_)

**2e*^anti^*** -^1^H-^13^C HSQCED (CDCl_3_)

**2e*^anti^*** -^1^H-^13^C HMBC (CDCl_3_)

**(1α,2α,3α,4α)-1,2,3,4-tetrahydronaphtho[1,2,3,4]bis(oxirene)-7-yl)methyl acetate (2e*^syn^*)**

**2e*^syn^*** - ^1^H NMR (400 MHz, CDCl_3_)

**2e*^syn^*** -^13^C NMR (100 MHz, CDCl_3_)

**2e*^syn^*** -DEPT (CDCl_3_)

**2e*^syn^*** -DEPTQ (CDCl_3_)

**2e*^syn^*** -^1^H-^1^H COSY (CDCl_3_)

**2e*^syn^*** -^1^H-^13^C HSQCED (CDCl_3_)

**2e*^syn^*** -^1^H-^13^C HMBC (CDCl_3_)

**(1α,2α,3β,4β)-1,2,3,4-tetrahydronaphtho[1,2,3,4]bis(oxirene)-6-yl)acetonitrile (2f*^anti^*)**

**2f*^anti^*** - ^1^H NMR (400 MHz, CDCl_3_)

**2f*^anti^*** -^13^C NMR (100 MHz, CDCl_3_)

**2f*^anti^*** -DEPT (CDCl_3_)

**2f*^anti^*** -DEPTQ (CDCl_3_)

**2f*^anti^*** -^1^H-^1^H COSY (CDCl_3_)

**2f*^anti^*** -^1^H-^13^C HSQCED (CDCl_3_)

**2f*^anti^*** -^1^H-^13^C HMBC (CDCl_3_)

**(1α,2α,3α,4α)-1,2,3,4-tetrahydronaphtho[1,2,3,4]bis(oxirene)-6-yl)acetonitrile (2f*^syn^*)**

**2f*^syn^*** - ^1^H NMR (400 MHz, CDCl_3_)

**2f*^syn^*** -^13^C NMR (100 MHz, CDCl_3_)

**2f*^syn^*** -DEPT (CDCl_3_)

**2f*^syn^*** -DEPTQ (CDCl_3_)

**2f*^syn^*** -^1^H-^1^H COSY (CDCl_3_)

**2f*^syn^*** -^1^H-^13^C HSQCED (CDCl_3_)

**2f*^syn^*** -^1^H-^13^C HMBC (CDCl_3_)

**(1α,2α,3β,4β)-1,2,3,4-tetrahydronaphtho[1,2,3,4]bis(oxirene)-6-yl)methyl acetamide (2g*^anti^*)**

**2g*^anti^*** - ^1^H NMR (400 MHz, CD_2_Cl_2_)

**2g*^anti^*** -^13^C NMR (100 MHz, CD_2_Cl_2_)

**2g*^anti^*** -DEPT (CD_2_Cl_2_)

**2g*^anti^*** -DEPTQ (CD_2_Cl_2_)

**2g*^anti^*** -^1^H-^1^H COSY (CD_2_Cl_2_)

**2g*^anti^*** -^1^H-^13^C HSQCED (CD_2_Cl_2_)

**2g*^anti^*** -^1^H-^13^C HMBC (CD_2_Cl_2_)

**(1α,2α,3α,4α)-1,2,3,4-tetrahydronaphtho[1,2,3,4]bis(oxirene)-6-yl)methyl acetamide (2g*^syn^*)**

**2g*^syn^*** - ^1^H NMR (400 MHz, CD_2_Cl_2_)

**2g*^syn^*** -^13^C NMR (100 MHz, CD_2_Cl_2_)

**2g*^syn^*** -DEPT (CD_2_Cl_2_)

**2g*^syn^*** -^1^H-^1^H COSY (CD_2_Cl_2_)

**2g*^syn^*** -^1^H-^13^C HSQCED (CD_2_Cl_2_)

**2g*^syn^*** -^1^H-^13^C HMBC (CD_2_Cl_2_)

**(4α,5α,6β,7β)-4,5,6,7-tetrahydronaphtho[4,5:6,7]bis(oxirene)-1-yl acetate (2h*^anti^*)**

**2h*^anti^*** - ^1^H NMR (400 MHz, CDCl_3_)

**2h*^anti^*** -^13^C NMR (100 MHz, CDCl_3_)

**2h*^anti^*** -DEPT (CDCl_3_)

**2h*^anti^*** -^1^H-^1^H COSY (CDCl_3_)

**2h*^anti^*** -^1^H-^13^C HSQCED (CDCl_3_)

**(5α,6α,7β,8β)-5,6,7,8-tetrahydronaphtho[5,6:7,8]bis(oxirene)-1,2-diyl acetate (2i*^anti^*)**

**2i*^anti^*** - ^1^H NMR (400 MHz, CDCl_3_)

**2i*^anti^*** -^13^C NMR (100 MHz, CDCl_3_)

**2i*^anti^***  -DEPT (CDCl_3_)

**2i*^anti^*** -^1^H-^1^H COSY (CDCl_3_)

**2i*^anti^*** -^1^H-^13^C HSQCED (CDCl_3_)

**(4α,5α,6β,7β)-1-nitro-4,5,6,7-tetrahydronaphtho[4,5:6,7]bis(oxirene) (2l*^anti^*)**

**2l*^anti^*** - ^1^H NMR (400 MHz, CDCl_3_)

**2l*^anti^*** -^13^C NMR (100 MHz, CDCl_3_)

**2l*^anti^*** -DEPT (CDCl_3_)

**2l*^anti^*** -DEPTQ (CDCl_3_)

**2l*^anti^*** -^1^H-^1^H COSY (CDCl_3_)

**2l*^anti^*** -^1^H-^13^C HSQCED (CDCl_3_)

**2l*^anti^*** -^1^H-^13^C HMBC (CDCl_3_)

**(4α,5α,6α,7α)-1-nitro-4,5,6,7-tetrahydronaphtho[4,5:6,7]bis(oxirene) (2l*^syn^*)**

**2l*^syn^*** - ^1^H NMR (400 MHz, CDCl_3_)

**2l*^syn^*** -^13^C NMR (100 MHz, CDCl_3_)

**2l*^syn^*** -DEPT (CDCl_3_)

**2l*^syn^*** -DEPTQ (CDCl_3_)

**2l*^syn^*** -^1^H-^1^H COSY (CDCl_3_)

**2l*^syn^*** -^1^H-^13^C HSQCED (CDCl_3_)

**2l*^syn^*** -^1^H-^13^C HMBC (CDCl_3_)

**(1α,2β,3α,4β)-1,4-diazido-1,2,3,4-tetrahydronaphthalene-2,3-diol (6e*^anti^*)**

**6e*^anti^*** - ^1^H NMR (400 MHz, CD_3_CN)

**6e*^anti^*** -^13^C NMR (100 MHz, CD_3_CN)

**6e*^anti^*** -DEPT (CD_3_CN)

**6e*^anti^*** -DEPTQ (CD_3_CN)

**6e*^anti^*** -^1^H-^1^H COSY (CD_3_CN)

**6e*^anti^*** -^1^H-^13^C HSQCED (CD_3_CN)

**6e*^anti^*** -^1^H-^13^C HMBC (CD_3_CN)

**(1α,2β,3β,4α)-1,4-diazido-1,2,3,4-tetrahydronaphthalene-2,3-diol (6e*^syn^*)**

**6e*^syn^*** - ^1^H NMR (400 MHz, CD_3_CN)

**6e*^syn^*** -^13^C NMR (100 MHz, CD_3_CN)

**6e*^syn^*** -DEPT (CD_3_CN)

**6e*^syn^*** -DEPTQ (CD_3_CN)

**6e*^syn^*** -^1^H-^1^H COSY (CD_3_CN)

**6e*^syn^*** -^1^H-^13^C HSQCED (CD_3_CN)

**6e*^syn^*** -^1^H-^13^C HMBC (CD_3_CN)

**1-((5α,6β,7α,8β)-5,8-diazido-6,7-dihydroxy-5,6,7,8-tetrahydronaphthalen-2-yl)ethan-1-one (2j*^anti^*)**

**2j*^anti^*** - ^1^H NMR (400 MHz, CD_3_CN)

**2j*^anti^*** -^13^C NMR (100 MHz, CD_3_CN)

**2j*^anti^*** -DEPT (CD_3_CN)

**2j*^anti^*** -DEPTQ (CD_3_CN)

**2j*^anti^*** -^1^H-^1^H COSY (CD_3_CN)

**2j*^anti^*** -^1^H-^13^C HSQCED (CD_3_CN)

**2j*^anti^*** -^1^H-^13^C HMBC (CD_3_CN)

**1-((5α,6β,7β,8α)-5,8-diazido-6,7-dihydroxy-5,6,7,8-tetrahydronaphthalen-2-yl)ethan-1-one (2j*^syn^*)**

**2j*^syn^*** - ^1^H NMR (400 MHz, CD_3_CN)

**2j*^syn^*** -^13^C NMR (100 MHz, CD_3_CN)

**2j*^syn^*** -DEPT (CD_3_CN)

**2j*^syn^*** -DEPTQ (CD_3_CN)

**2j*^syn^*** -^1^H-^1^H COSY (CD_3_CN)

**2j*^syn^*** -^1^H-^13^C HSQCED (CD_3_CN)

**2j*^syn^*** -^1^H-^13^C HMBC (CD_3_CN)

**(5α,6β,7α,8β)-5,8-diazido-6,7-dihydroxy-5,6,7,8-tetrahydronaphthalene-2-carbonitrile (2k***^a^****^nti^*)**

**2k*^anti^*** - ^1^H NMR (400 MHz, CD_3_CN)

**2k*^anti^*** -^13^C NMR (100 MHz, CD_3_CN)

**2k*^anti^*** -DEPT (CD_3_CN)

**2k*^anti^*** -DEPTQ (CD_3_CN)

**2k*^anti^*** -^1^H-^1^H COSY (CD_3_CN)

**2k*^anti^*** -^1^H-^13^C HSQCED (CD_3_CN)

**2k*^anti^*** -^1^H-^13^C HMBC (CD_3_CN)

**(5α,6β,7β,8α)-5,8-diazido-6,7-dihydroxy-5,6,7,8-tetrahydronaphthalene-2-carbonitrile (2k*^syn^*)**

**2k*^syn^*** - ^1^H NMR (400 MHz, CD_3_CN)

**2k*^syn^*** -^13^C NMR (100 MHz, CD_3_CN)

**2k*^syn^*** -DEPT (CD_3_CN)

**2k*^syn^*** -DEPTQ (CD_3_CN)

**2k*^syn^*** -^1^H-^1^H COSY (CD_3_CN)

**2k*^syn^*** -^1^H-^13^C HSQCED (CD_3_CN)

**2k*^syn^*** -^1^H-^13^C HMBC (CD_3_CN)

**(5α,6β,7α,8β)-5,8-diazido-6,7-dihydroxy-5,6,7,8-tetrahydronaphthalene-1-carbonitrile (2q*^anti^*)**

**2q*^anti^*** - ^1^H NMR (400 MHz, CD_3_CN)

**2q*^anti^*** -^13^C NMR (100 MHz, CD_3_CN)

**2q*^anti^*** -DEPT (CD_3_CN)

**2q*^anti^*** -DEPTQ (CD_3_CN)

**2q*^anti^*** -^1^H-^1^H COSY (CD_3_CN)

**2q*^anti^*** -^1^H-^13^C HSQCED (CD_3_CN)

**2q*^anti^*** -^1^H-^13^C HMBC (CD_3_CN)

**(5α,6β,7β,8α)-5,8-diazido-6,7-dihydroxy-5,6,7,8-tetrahydronaphthalene-1-carbonitrile (2q*^syn^*)**

**2q*^syn^*** - ^1^H NMR (400 MHz, CD_3_CN)

**2q*^syn^*** -^13^C NMR (100 MHz, CD_3_CN)

**2q*^syn^*** -DEPT (CD_3_CN)

**2q*^syn^*** -DEPTQ (CD_3_CN)

**2q*^syn^*** -^1^H-^1^H COSY (CD_3_CN)

**2q*^syn^*** -^1^H-^13^C HSQCED (CD_3_CN)

**2q*^syn^*** -^1^H-^13^C HMBC (CD_3_CN)

**(1α,2β,3α,4β)-1,4-diazido-6-ethyl-1,2,3,4-tetrahydronaphthalene-2,3-diol (2b*^anti^*)**

**2b*^anti^*** - ^1^H NMR (400 MHz, CD_3_CN)

**2b*^anti^*** -^13^C NMR (100 MHz, CD_3_CN)

**2b*^anti^*** -DEPT (CD_3_CN)

**2b*^anti^*** -DEPTQ (CD_3_CN)

**2b*^anti^*** -^1^H-^1^H COSY (CD_3_CN)

**2b*^anti^*** -^1^H-^13^C HSQCED (CD_3_CN)

**2b*^anti^*** -^1^H-^13^C HMBC (CD_3_CN)

**(1α,2β,3α,4β)-1,4-diazido-1-ethyl-1,2,3,4-tetrahydronaphthalene-2,3-diol (2o*^anti^*)**

**2o*^anti^*** - ^1^H NMR (400 MHz, CD_3_CN)

**2o*^anti^*** -^13^C NMR (100 MHz, CD_3_CN)

**2o*^anti^*** -DEPT (CD_3_CN)

**2o*^anti^*** -DEPTQ (CD_3_CN)

**2o*^anti^*** -^1^H-^1^H COSY (CD_3_CN)

**2o*^anti^*** -^1^H-^13^C HSQCED (CD_3_CN)

**2o*^anti^*** -^1^H-^13^C HMBC (CD_3_CN)

**(1α,2β,3α,4β)-1,4-diazido-1-methyl-1,2,3,4-tetrahydronaphthalene-2,3-diol (2p*^anti^*)**

**2q*^anti^*** - ^1^H NMR (400 MHz, CD_3_CN)

**2q*^anti^*** -^13^C NMR (100 MHz, CD_3_CN)

**2q*^anti^*** -DEPT (CD_3_CN)

**2q*^anti^*** -DEPTQ (CD_3_CN)

**2q*^anti^*** -^1^H-^1^H COSY (CD_3_CN)

**2q*^anti^*** -^1^H-^13^C HSQCED (CD_3_CN)

**2q*^anti^*** -^1^H-^13^C HMBC (CD_3_CN)

**(1α,2β,3α,4β)-1,4-diazido-6-bromo-1,2,3,4-tetrahydronaphthalene-2,3-diol (2m*^anti^*)**

**2m*^anti^***  - ^1^H NMR (400 MHz, CD_3_CN)

**2m*^anti^*** -^13^C NMR (100 MHz, CD_3_CN)

**2m*^anti^*** -DEPT (CD_3_CN)

**2m*^anti^*** -DEPTQ (CD_3_CN)

**2m*^anti^*** -^1^H-^1^H COSY (CD_3_CN)

**2m*^anti^*** -^1^H-^13^C HSQCED (CD_3_CN)

**2m*^anti^*** -^1^H-^13^C HMBC (CD_3_CN)

**(1α,2β,3β,4α)-1,4-diazido-6-bromo-1,2,3,4-tetrahydronaphthalene-2,3-diol (2m*^syn^*)**

**2m*^syn^*** - ^1^H NMR (400 MHz, CD_3_CN)

**2m*^syn^*** -^13^C NMR (100 MHz, CD_3_CN)

**2m*^syn^*** -DEPT (CD_3_CN)

**2m*^syn^*** -DEPTQ (CD_3_CN)

**2m*^syn^*** -^1^H-^1^H COSY (CD_3_CN)

**2m*^syn^*** -^1^H-^13^C HSQCED (CD_3_CN)

**2m*^syn^*** -^1^H-^13^C HMBC (CD_3_CN)

**(1α,2β,3α,4β)-1,4-diazido-5-bromo-1,2,3,4-tetrahydronaphthalene-2,3-diol (2r*^anti^*)**

**2r*^anti^*** - ^1^H NMR (400 MHz, CD_3_CN)

**2r*^anti^*** -^13^C NMR (100 MHz, CD_3_CN)

**(1α,2β,3β,4α)-1,4-diazido-5-bromo-1,2,3,4-tetrahydronaphthalene-2,3-diol (2r*^syn^*)**

**2r*^syn^*** - ^1^H NMR (400 MHz, CD_3_CN)

**2r*^syn^*** -^13^C NMR (100 MHz, CD_3_CN)

**1,4-diazido-5-bromo-1,2,3,4-tetrahydronaphthalene-2,3-diol (2r)**

**2r** -DEPT (CD_3_CN)

**2r** -DEPTQ (CD_3_CN)

**2r** -^1^H-^1^H COSY (CD_3_CN)

**2r** -^1^H-^13^C HSQCED (CD_3_CN)

**2r** -^1^H-^13^C HMBC (CD_3_CN)

**(1α,2β,3α,4β)-1,4-diazido-6-chloro-1,2,3,4-tetrahydronaphthalene-2,3-diol (2n*^anti^*)**

**2n*^anti^*** - ^1^H NMR (400 MHz, CD_3_CN)

**2n*^anti^*** -^13^C NMR (100 MHz, CD_3_CN)

**2n*^anti^*** -DEPT (CD_3_CN)

**2n*^anti^*** -DEPTQ (CD_3_CN)

**2n*^anti^*** -^1^H-^1^H COSY (CD_3_CN)

**2n*^anti^*** -^1^H-^13^C HSQCED (CD_3_CN)

**2n*^anti^*** -^1^H-^13^C HMBC (CD_3_CN)

**(1α,2β,3β,4α)-1,4-diazido-6-chloro-1,2,3,4-tetrahydronaphthalene-2,3-diol (2n*^syn^*)**

**2n*^syn^*** - ^1^H NMR (400 MHz, CD_3_CN)

**2n*^syn^*** -^13^C NMR (100 MHz, CD_3_CN)

**2n*^syn^*** -DEPT (CD_3_CN)

**2n*^syn^*** -DEPTQ (CD_3_CN)

**2n*^syn^*** -^1^H-^1^H COSY (CD_3_CN)

**2n*^syn^*** -^1^H-^13^C HSQCED (CD_3_CN)

**2n*^syn^*** -^1^H-^13^C HMBC (CD_3_CN)

**(1α,2β,3α,4β)-1,4-diazido-6,7-dimethyl-1,2,3,4-tetrahydronaphthalene-2,3-diol (2c*^anti^*)**

**2c*^anti^*** - ^1^H NMR (400 MHz, CD_3_CN)

**2c*^anti^*** -^13^C NMR (100 MHz, CD_3_CN)

**2c*^anti^*** -DEPT (CD_3_CN)

**2c*^anti^*** -DEPTQ (CD_3_CN)

**2c*^anti^*** -^1^H-^1^H COSY (CD_3_CN)

**2c*^anti^*** -^1^H-^13^C HSQCED (CD_3_CN)

**2c*^anti^*** -^1^H-^13^C HMBC (CD_3_CN)

**(1α,2β,3β,4α)-1,4-diazido-6,7-dimethyl-1,2,3,4-tetrahydronaphthalene-2,3-diol (2c*^syn^*)**

**2c*^syn^*** - ^1^H NMR (400 MHz, CD_3_CN)

**2c*^syn^*** -^13^C NMR (100 MHz, CD_3_CN)

**2c*^syn^*** -DEPT (CD_3_CN)

**2c*^syn^*** -DEPTQ (CD_3_CN)

**2c*^syn^*** -^1^H-^1^H COSY (CD_3_CN)

**2c*^syn^*** -^1^H-^13^C HSQCED (CD_3_CN)

**2c*^syn^*** -^1^H-^13^C HMBC (CD_3_CN)

**(1α,2β,3α,4β)-1,4-diazido-6-phenyl-1,2,3,4-tetrahydronaphthalene-2,3-diol (2d*^anti^*)**

**2d*^anti^*** - ^1^H NMR (400 MHz, CD_3_CN)

**2d*^anti^*** -^13^C NMR (100 MHz, CD_3_CN)

**2d*^anti^*** -DEPT (CD_3_CN)

**2d*^anti^*** -DEPTQ (CD_3_CN)

**2d*^anti^*** -^1^H-^1^H COSY (CD_3_CN)

**2d*^anti^*** -^1^H-^13^C HSQCED (CD_3_CN)

**2d*^anti^*** -^1^H-^13^C HMBC (CD_3_CN)

**(1α,2β,3β,4α)-1,4-diazido-6-phenyl-1,2,3,4-tetrahydronaphthalene-2,3-diol (2d*^syn^*)**

**2d*^syn^*** - ^1^H NMR (400 MHz, CD_3_CN)

**2d*^syn^*** -^13^C NMR (100 MHz, CD_3_CN)

**2d*^syn^*** -DEPT (CD_3_CN)

**2d*^syn^*** -DEPTQ (CD_3_CN)

**2d*^syn^*** -^1^H-^1^H COSY (CD_3_CN)

**2d*^syn^*** -^1^H-^13^C HSQCED (CD_3_CN)

**2d*^syn^*** -^1^H-^13^C HMBC (CD_3_CN)

**(1α,2β,3α,4β)-1,4-diazido-5-fluoro-1,2,3,4-tetrahydronaphthalene-2,3-diol (2s*^anti^*)**

**2s*^anti^*** - ^1^H NMR (400 MHz, CD_3_CN)

**2s*^anti^*** -^19^F NMR (377 MHz, CD_3_CN)

**2s*^anti^*** -^13^C NMR (100 MHz, CD_3_CN)

**2s*^anti^*** -DEPT (CD_3_CN)

**2s*^anti^*** -DEPTQ (CD_3_CN)

**2s*^anti^*** -^1^H-^1^H COSY (CD_3_CN)

**2s*^anti^*** -^1^H-^13^C HSQCED (CD_3_CN)

**2s*^anti^*** -^1^H-^13^C HMBC (CD_3_CN)

**(1α,2β,3β,4α)-1,4-diazido-5-fluoro-1,2,3,4-tetrahydronaphthalene-2,3-diol (2s*^syn^*)**

**2s*^syn^*** - ^1^H NMR (400 MHz, CD_3_CN)

**2s*^syn^*** -^13^C NMR (100 MHz, CD_3_CN)

**2s*^syn^*** -^19^F NMR (377 MHz, CD_3_CN)

**2s*^syn^*** -DEPT (CD_3_CN)

**2s*^syn^*** -DEPTQ (CD_3_CN)

**2s*^syn^*** -^1^H-^1^H COSY (CD_3_CN)

**2s*^syn^*** -^1^H-^13^C HSQCED (CD_3_CN)

**2s*^syn^*** -^1^H-^13^C HMBC (CD_3_CN)

**(5α,6α,7β,8β)-2-methyl-5,6,7,8-tetrahydronaphtho[5,6:7,8]bis(oxirene)-1-yl acetate (2t*^anti^*)**

**2t*^anti^*** - ^1^H NMR (400 MHz, CDCl_3_)

**2t*^anti^*** -^13^C NMR (100 MHz, CDCl_3_)

**2t*^anti^*** -DEPT (CDCl_3_)

**2t*^anti^*** -DEPTQ (CDCl_3_)

**2t*^anti^*** -^1^H-^1^H COSY (CDCl_3_)

**2t*^anti^*** -^1^H-^13^C HSQCED (CDCl_3_)

**2t*^anti^*** -^1^H-^13^C HMBC (CDCl_3_)

**(4α,5α,6β,7β)-2-methyl-4,5,6,7,-tetrahydronaphtho[4,5:6,7]bis(oxirene)-1-yl acetate (2u*^anti^*)**

**2u*^anti^*** - ^1^H NMR (400 MHz, CDCl_3_)

**2u*^anti^*** -^13^C NMR (100 MHz, CDCl_3_)

**2u*^anti^*** -DEPT (CDCl_3_)

**2u*^anti^*** -DEPTQ (CDCl_3_)

**2u*^anti^*** -^1^H-^1^H COSY (CDCl_3_)

**2u*^anti^*** -^1^H-^13^C HSQCED (CDCl_3_)

**2u*^anti^*** -^1^H-^13^C HMBC (CDCl_3_)

**2-bromo-4,5,6,7,-tetrahydronaphtho[4,5:6,7]bis(oxirene)-1-yl acetate (2v)**

**(4α,5α,6β,7β)-2-bromo-4,5,6,7,-tetrahydronaphtho[4,5:6,7]bis(oxirene)-1-yl acetate (2v*^anti^*)**

**2v*^anti^*** - ^1^H NMR (400 MHz, CD_3_CN)

**2v*^anti^*** -^13^C NMR (100 MHz, CD_3_CN)

**2v*^anti^*** -DEPT (CD_3_CN)

**2v*^anti^*** -^1^H-^1^H COSY (CD_3_CN)

**2v*^anti^*** -^1^H-^13^C HSQCED (CD_3_CN)

**2v*^anti^*** -^1^H-^13^C HMBC (CD_3_CN)

**1,4-diazido-2-bromo-1-methyl-1,2,3,4-tetrahydronaphthalene-2,3-diol (2v.1)**

+

**2v.1** - ^1^H NMR (400 MHz, CD_3_CN)

**2v.1** -^13^C NMR (100 MHz, CD_3_CN)

**2v.1** -^1^H-^1^H COSY (CD_3_CN)

**2v.1** -^1^H-^13^C HSQCED (CD_3_CN)

**2v.1** -^1^H-^13^C HMBC (CD_3_CN)

**1,4-dimethoxy-1,2,3,4-tetrahydronaphthalene-2,3-diol (6i)**

**6i** -^1^H NMR (400 MHz, CDCl_3_)

**6i** -^13^C NMR (100 MHz, CDCl_3_)

**1,4-dimethyl-1,2,3,4-tetrahydronaphthalene-2,3-diol (6g)**

**6g** -^1^H NMR (400 MHz, CDCl_3_)

**6g** -^13^C NMR (100 MHz, CDCl_3_)

**1,4-bis(*p*-tolyloxy)-1,2,3,4-tetrahydronaphthalene-2,3-diol (6h)**

**6h** -^1^H NMR (400 MHz, CDCl_3_)

**6h** -^13^C NMR (100 MHz, CDCl_3_)

**1,4-bis(butylthio)-1,2,3,4-tetrahydronaphthalene-2,3-diol (6f)**

**6f** -^1^H NMR (400 MHz, CDCl_3_)

**6f** -^13^C NMR (100 MHz, CDCl_3_)

**6f** -COSY (CDCl_3_)

**6f** -DEPT (CDCl_3_)

**1,2,3,4-tetrahydronaphthalene-1,2,3,4-tetraol (6b)**

**6b** -^1^H NMR (400 MHz, CDCl_3_)

**6b** -^13^C NMR (100 MHz, CDCl_3_)

**1,2,3,4-tetrahydronaphthalene-2,3-diol (6a)**

**6a** -^1^H NMR (400 MHz, CDCl_3_)

**6a** -^13^C NMR (100 MHz, CDCl_3_)

**1,4-bis(dimethylamino)-1,2,3,4-tetrahydronaphthalene-2,3-diol (6d)**

**6d** -^1^H NMR (400 MHz, CDCl_3_)

**6d** -^13^C NMR (100 MHz, CDCl_3_)

**3-methoxy-1,2,3,7-tetrahydronaphtho[1,2]oxiren-2-ol (6c)**

**6c** -^1^H NMR (400 MHz, CDCl_3_)

**6c** -^13^C NMR (100 MHz, CDCl_3_)

**6c** -COSY (CDCl_3_)

# SCF and UV-Vis

For the racemic samples a mixture of *(S,S)*-Mn^(Tips,NMe2)2^pdp and *(R,R)*-Mn^(Tips,NMe2)2^pdp was manually mixed as powder and used for the catalysis. For this reason, some of the racemic samples do not correspond to exactly racemic mixtures, however the enantiomers were checked by UV-vis, and the spectra perfectly matched. The reaction crude or the racemic products was directly analyzed without further purification. The samples were diluted in acetonitrile and directly analyzed by SCF-HPLC.

**(1α,2α,3β,4β)-1,2,3,4-tetrahydronaphtho[1,2:3,4]bis(oxirene) (2a*^anti^*)**

Racemic


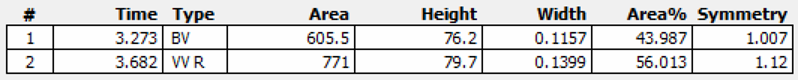


Chiral product

Conditions A


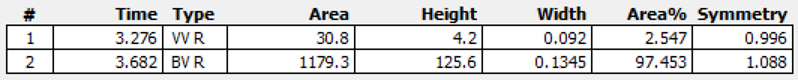


Conditions B


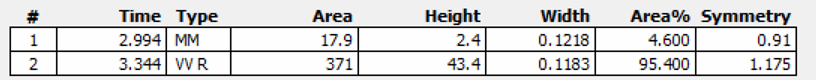


UV-vis racemic sample


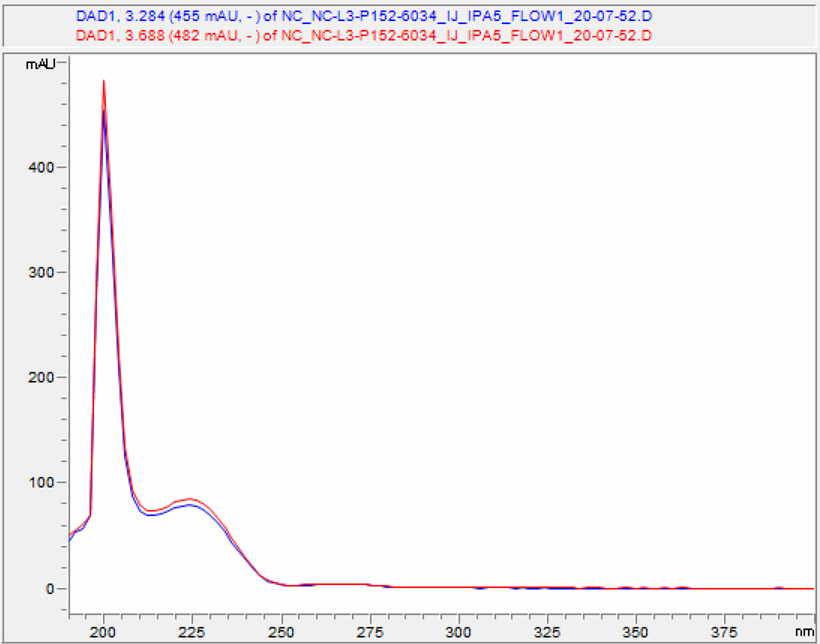


UV-vis chiral product


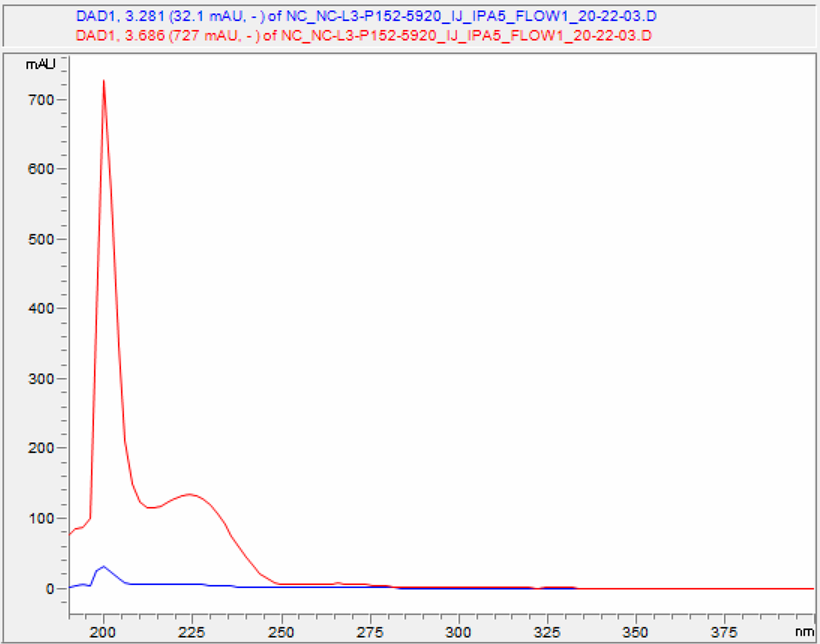


**(1α,2α,3β,4β)-1,2,3,4-tetrahydronaphtho[1,2,3,4]bis(oxirene)-7-yl)methyl acetate (2e*^anti^*)**

**
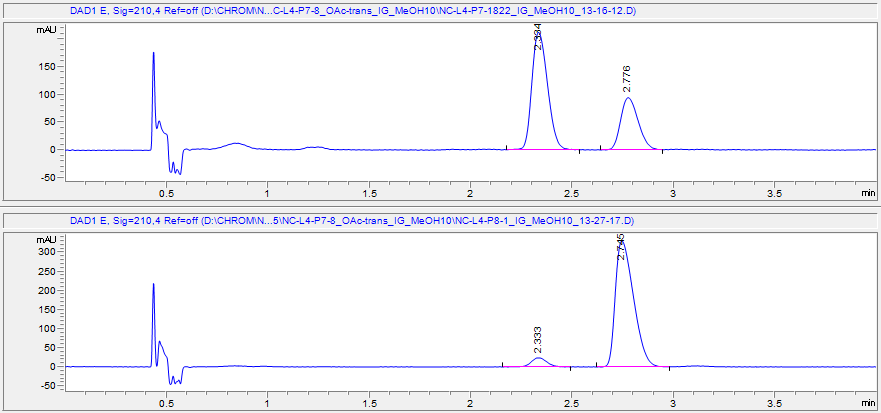
**

Conditions B


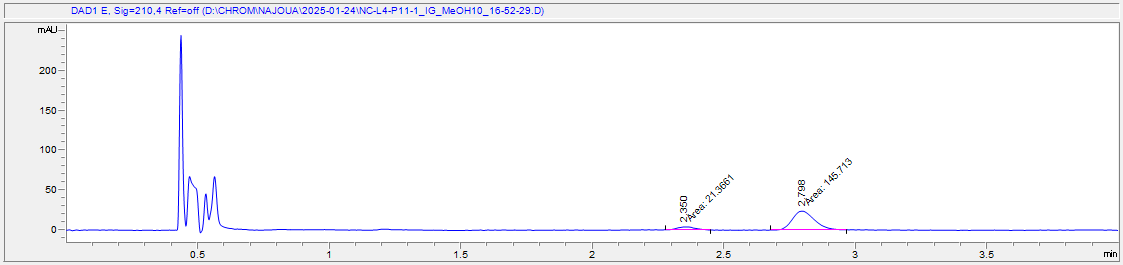


Racemic


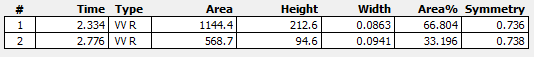


Chiral product

Conditions A


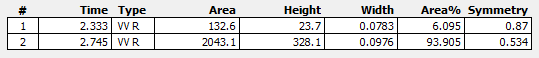


Conditions B


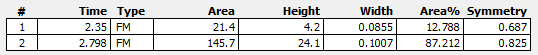


UV-vis racemic sample

**
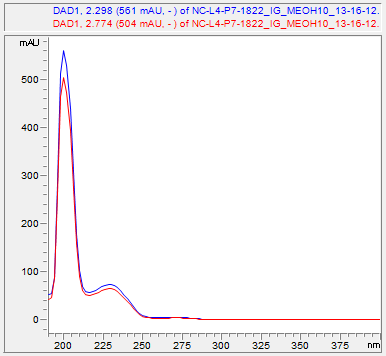
**

UV-vis chiral sample

**
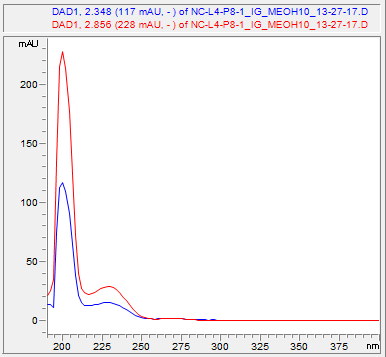
**

**(1α,2α,3α,4α)-1,2,3,4-tetrahydronaphtho[1,2,3,4]bis(oxirene)-7-yl)methyl acetate (2e*^syn^*)**


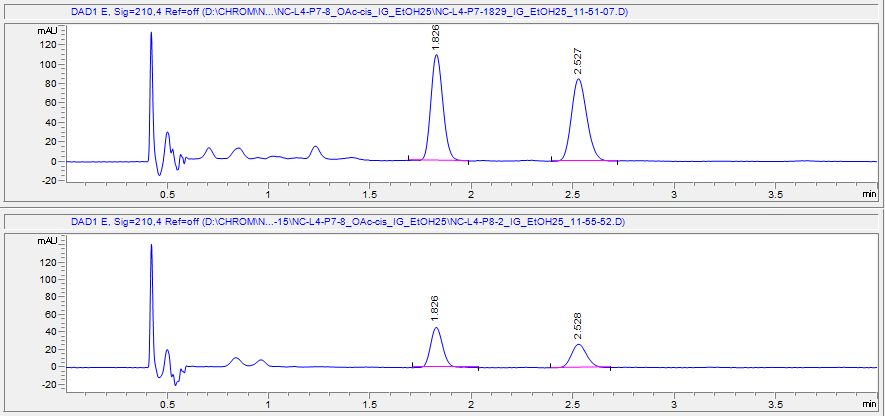


Conditions B


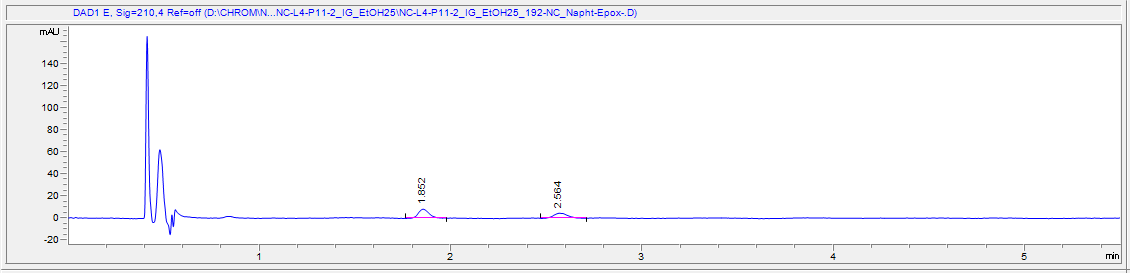


Racemic


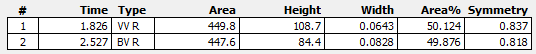


Chiral product

Conditions A

**
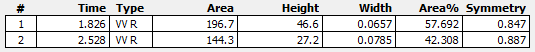
**

Conditions B

**
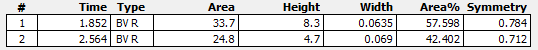
**

UV-vis racemic sample

**
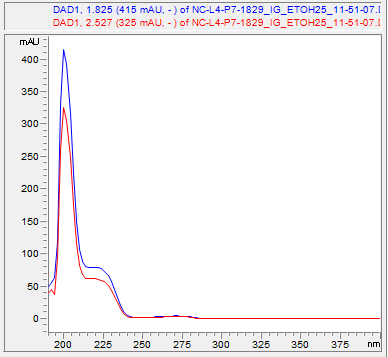
**

UV-vis chiral sample

**
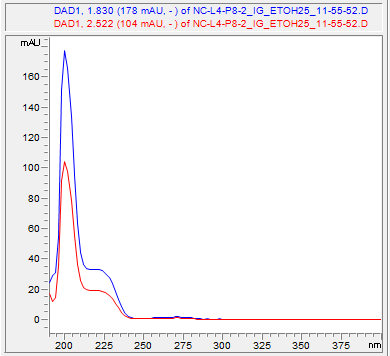
**

**(1α,2α,3β,4β)-1,2,3,4-tetrahydronaphtho[1,2,3,4]bis(oxirene)-6-yl)acetonitrile (2f*^anti^*)**

**
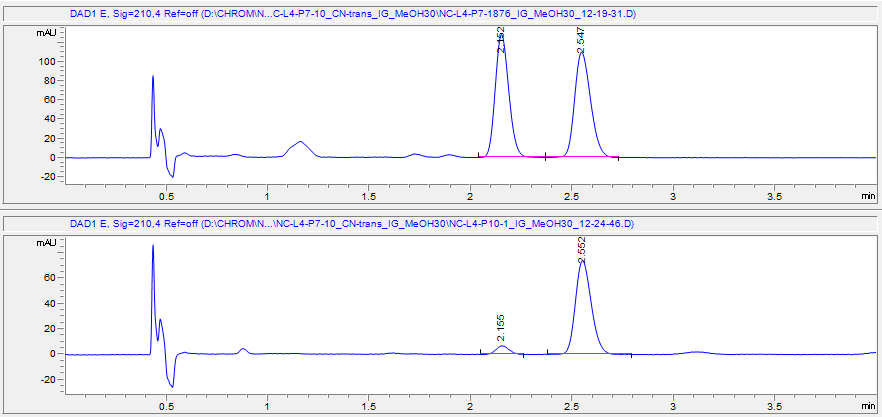
**

Conditions B

**
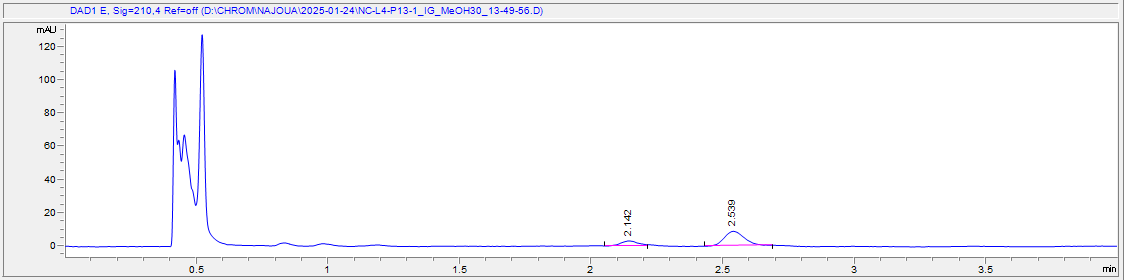
**

Racemic


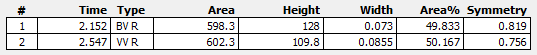


Chiral product

Conditions A


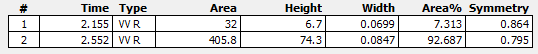


Conditions B


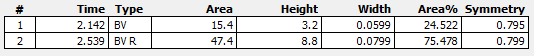


UV-vis racemic sample

**
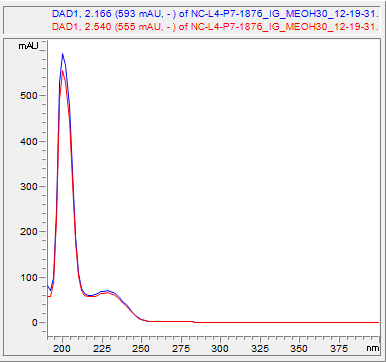
**

UV-vis chiral sample

**
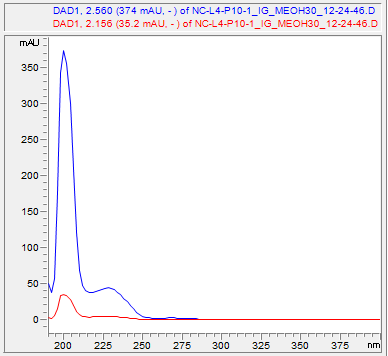
**

**(1α,2α,3α,4α)-1,2,3,4-tetrahydronaphtho[1,2,3,4]bis(oxirene)-6-yl)acetonitrile (2f*^syn^*)**

**
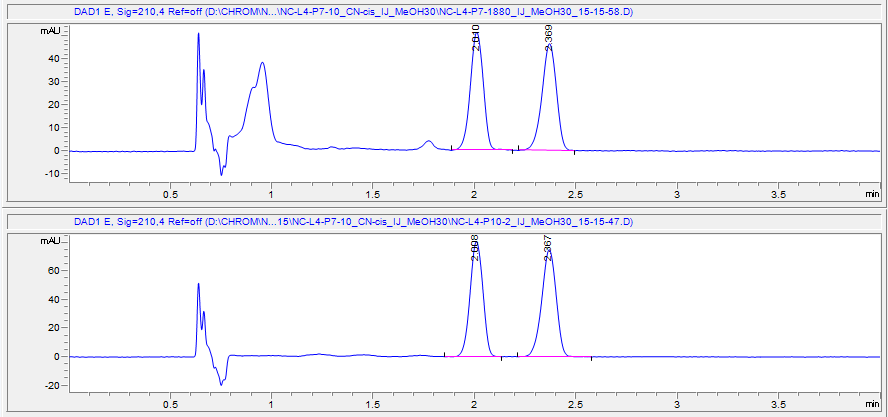
**

Conditions B

**
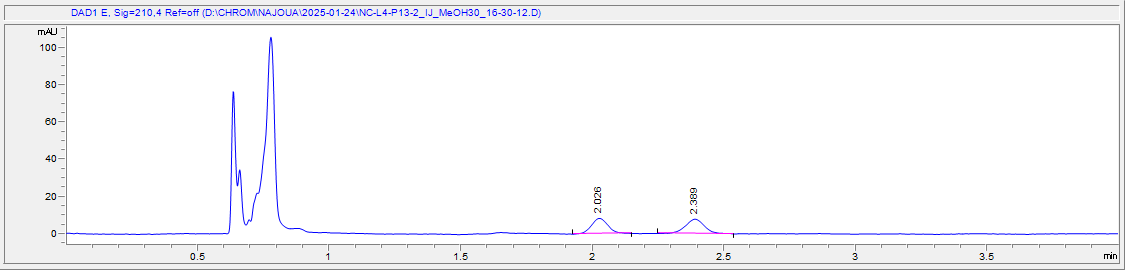
**

Racemic


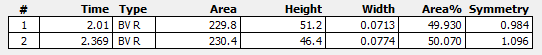


Chiral product

Conditions A


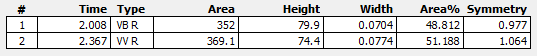


Conditions B


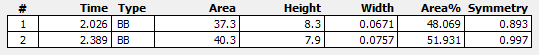


UV-vis racemic sample

**
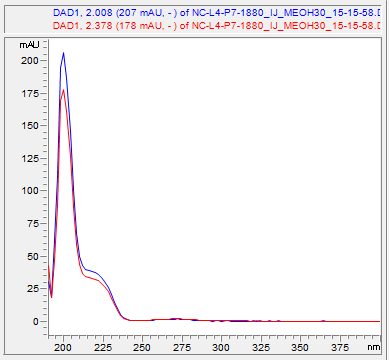
**

UV-vis chiral sample

**
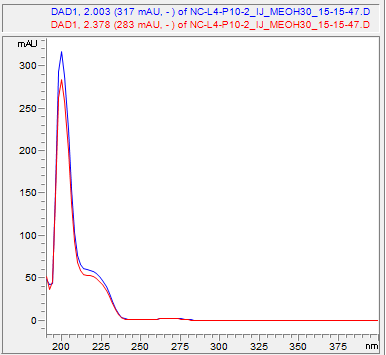
**

**(1α,2α,3β,4β)-1,2,3,4-tetrahydronaphtho[1,2,3,4]bis(oxirene)-6-yl)methyl acetamide (2g*^anti^*)**

**
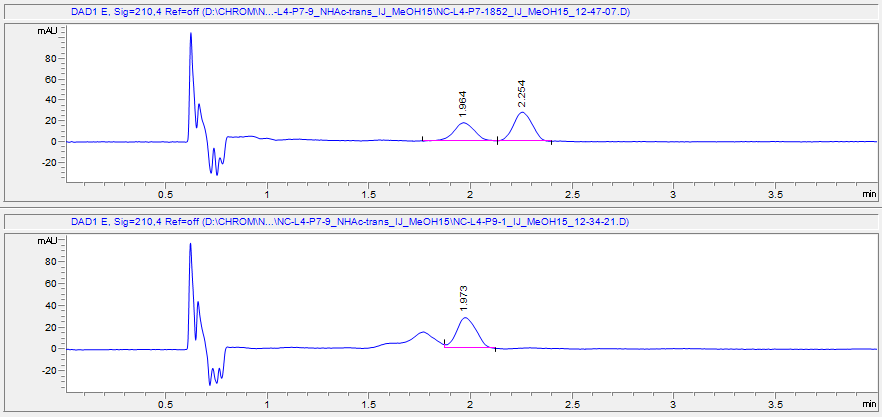
**

Conditions B

**
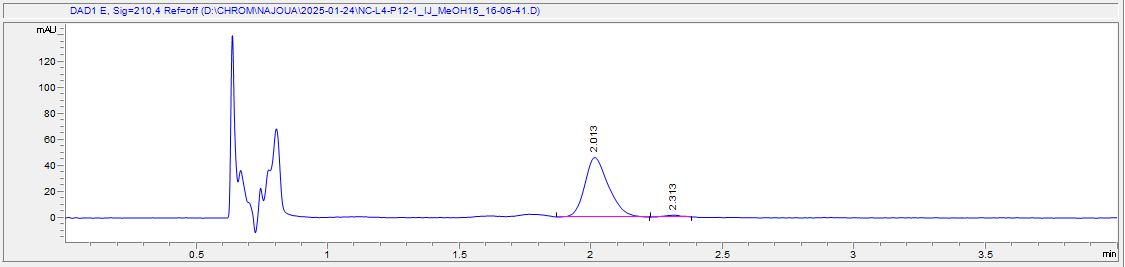
**

Racemic


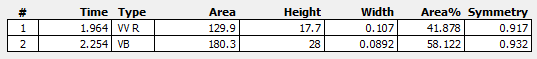


Chiral product: Conditions B


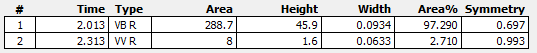


UV-vis racemic sample


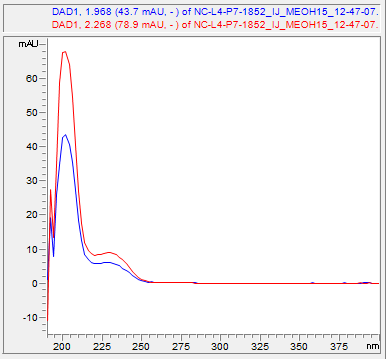


UV-vis quiral sample

**
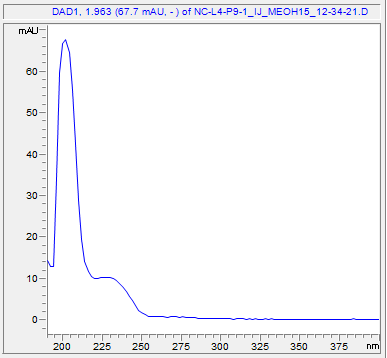
**

**(1α,2α,3α,4α)-1,2,3,4-tetrahydronaphtho[1,2,3,4]bis(oxirene)-6-yl)methyl acetamide (2g*^syn^*)**

**
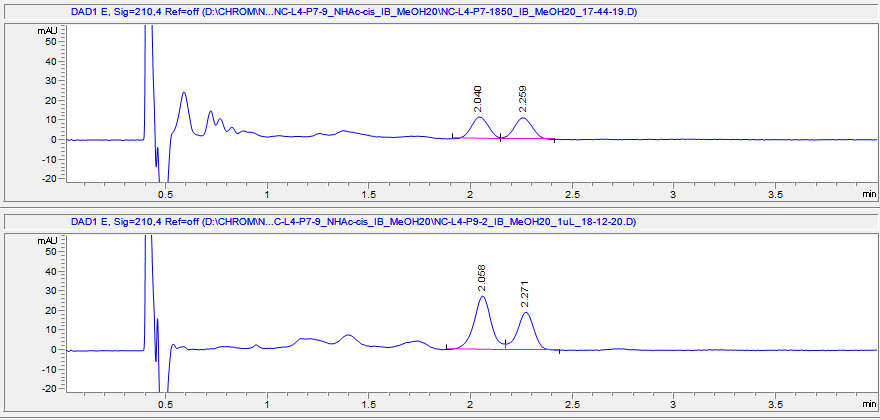
**

Conditions B

**
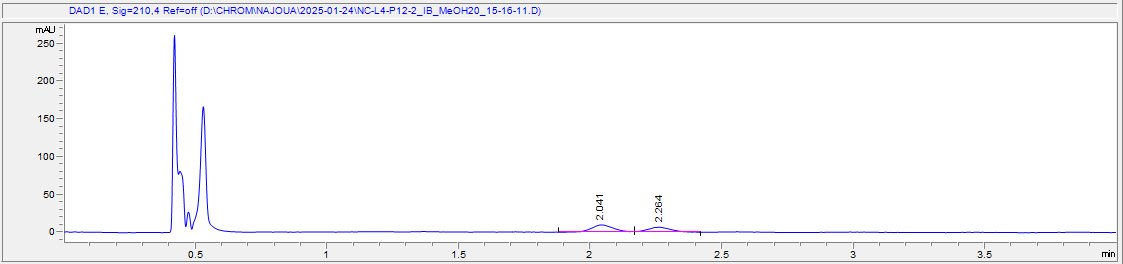
**

Racemic


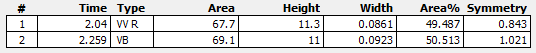


Chiral product

Conditions A


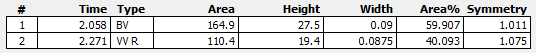


Conditions B


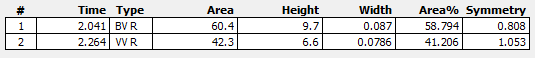


UV-vis racemic sample


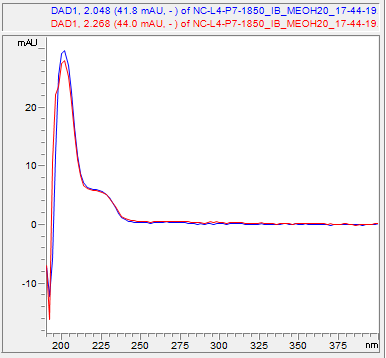


UV-vis quiral sample

**
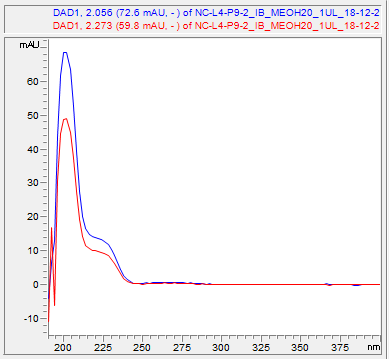
**

**(4α,5α,6β,7β)-4,5,6,7-tetrahydronaphtho[4,5:6,7]bis(oxirene)-1-yl acetate (2h*^anti^*)**

Racemic


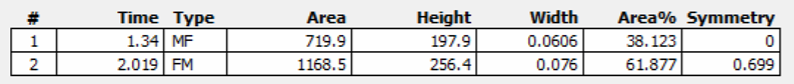


Chiral product

Conditions A

**
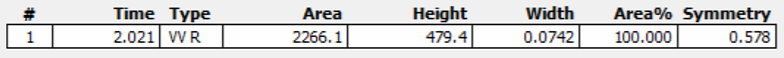
**

Conditions B

**
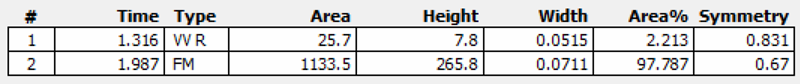
**

UV-vis racemic sample

**
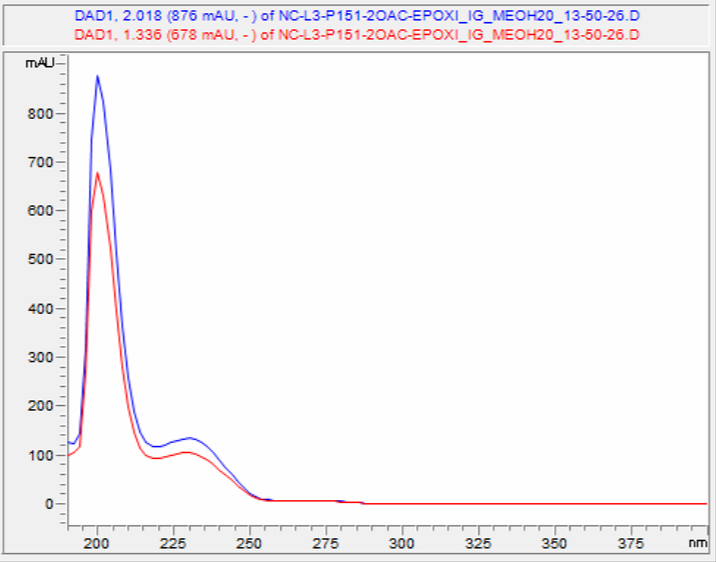
**

UV-vis chiral product

**
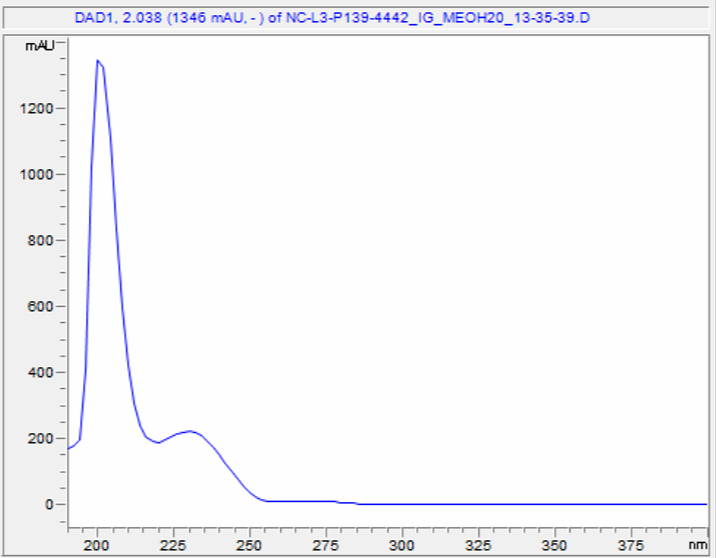
**

**(5α,6α,7β,8β)-5,6,7,8-tetrahydronaphtho[5,6:7,8]bis(oxirene)-1,2-diyl acetate (2i*^anti^*)**

Racemic


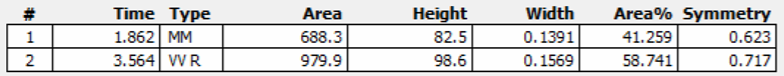


Chiral product

Conditions A

**
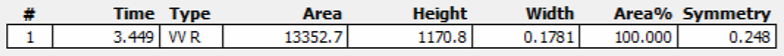
**

Conditions B

**
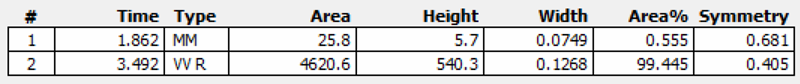
**

UV-vis racemic sample

**
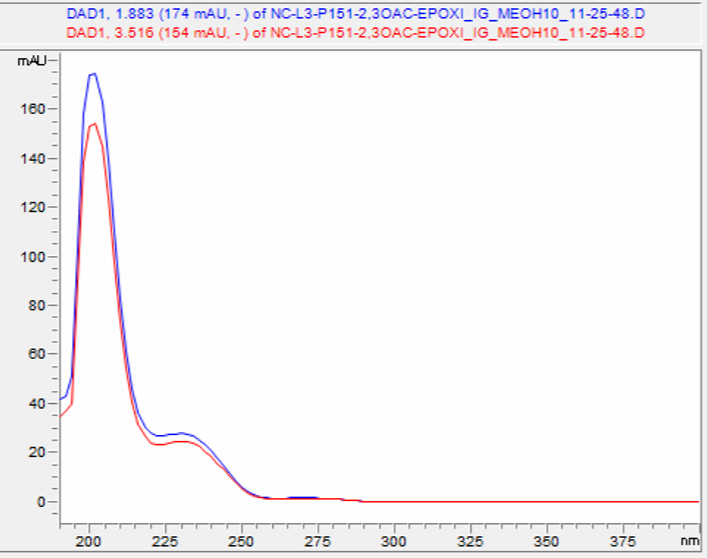
**

UV-vis chiral product

**
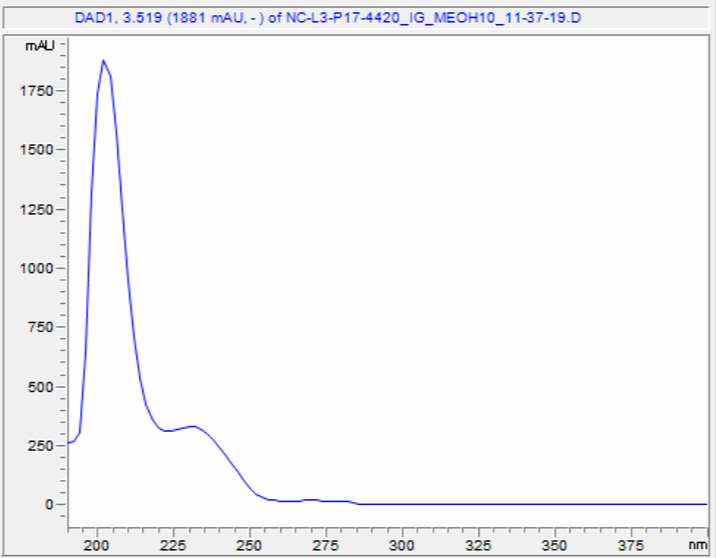
**

**1-nitro-4,5,6,7-tetrahydronaphtho[4,5:6,7]bis(oxirene) (2l)**

Racemic

**
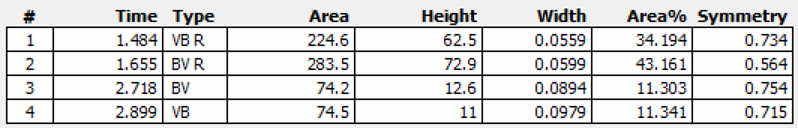
**

Conditions A

**
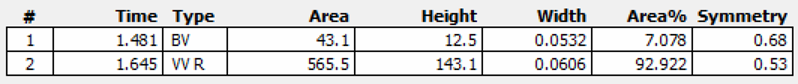
**

Conditions B

**
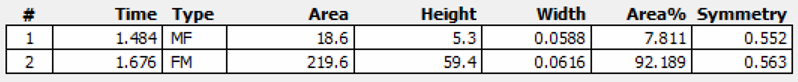
**

Conditions A

**
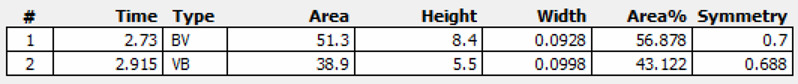
**

Conditions B

**
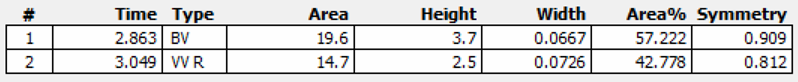
**

UV-vis racemic sample

**
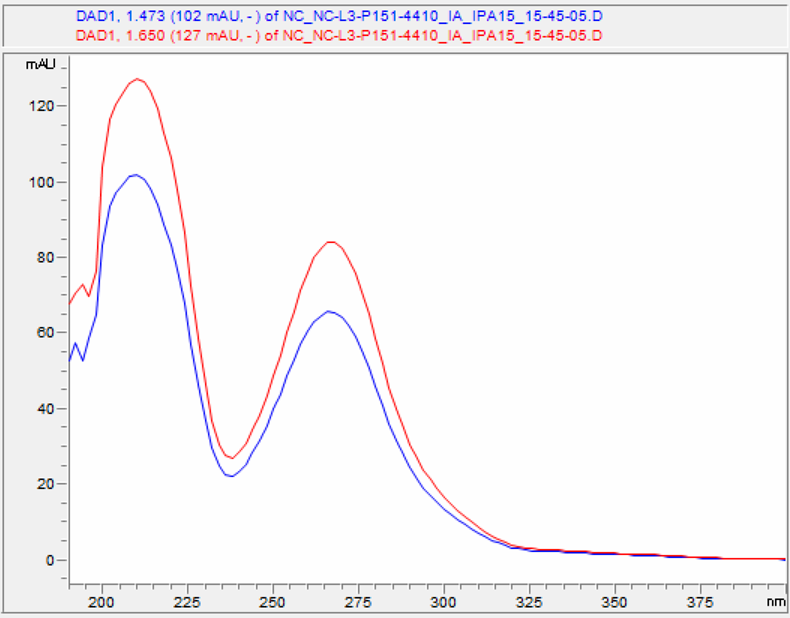
**

**
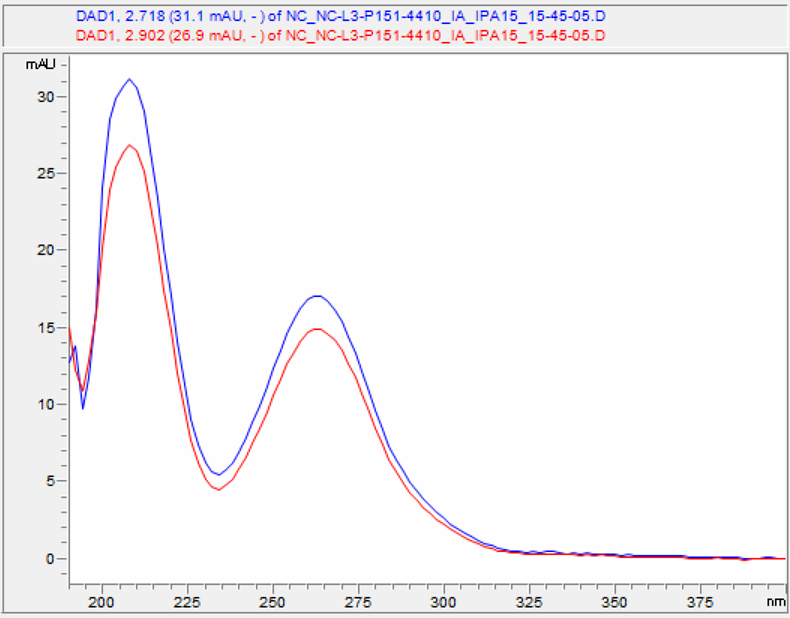
**

UV-vis chiral product

**
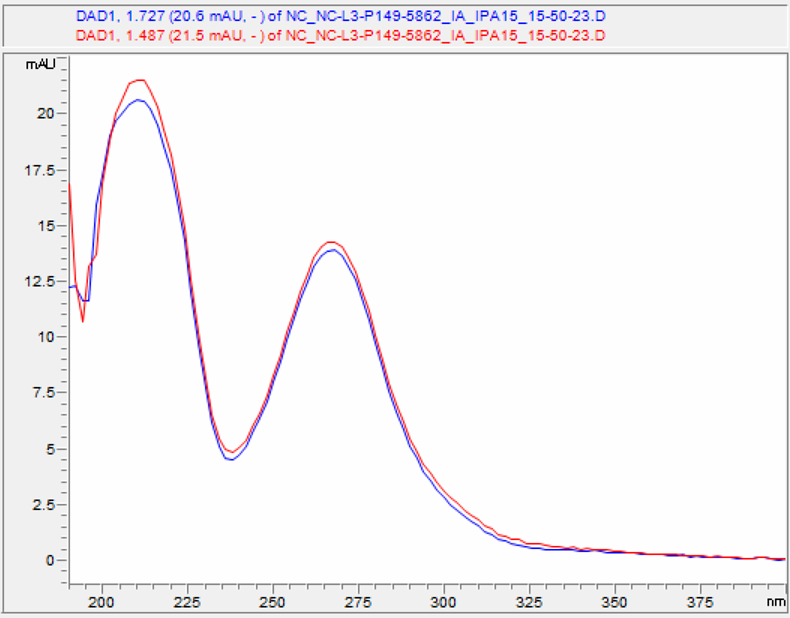
**

**
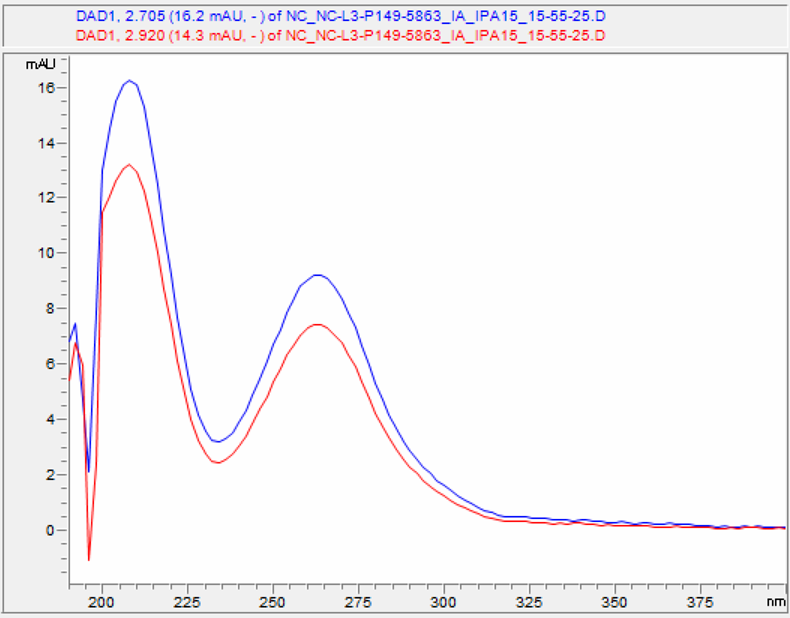
**

**(1α,2β,3α,4β)-1,4-diazido-1,2,3,4-tetrahydronaphthalene-2,3-diol (6e*^anti^*)**

Racemic


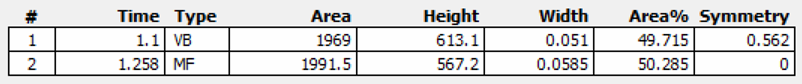


Chiral product

Conditions A

**
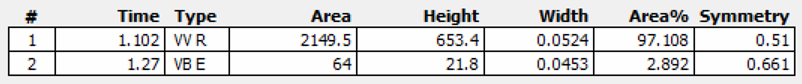
**

Conditions B


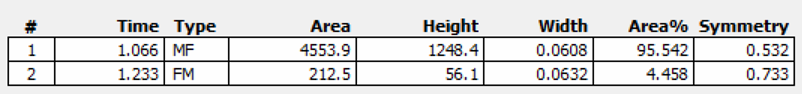


UV-vis racemic sample


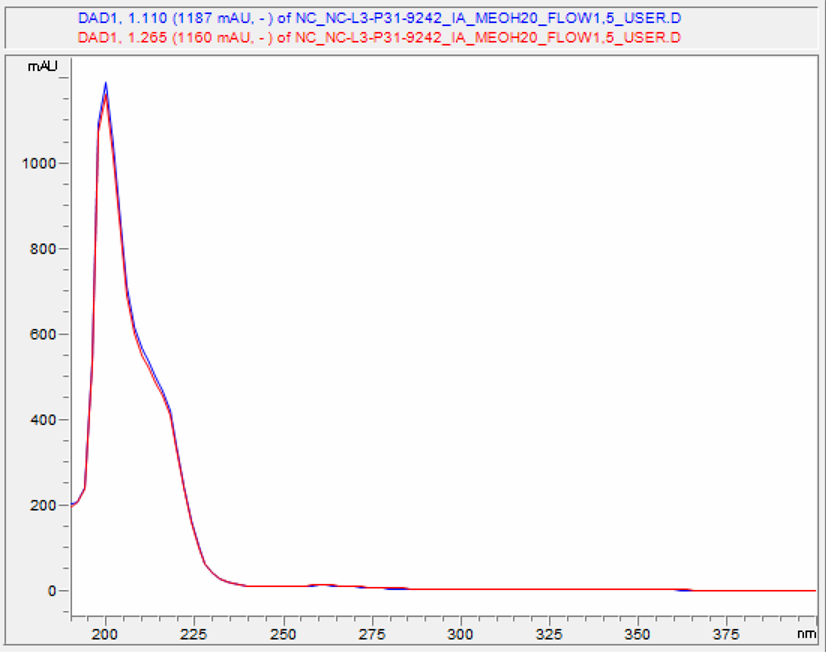


UV-vis chiral product


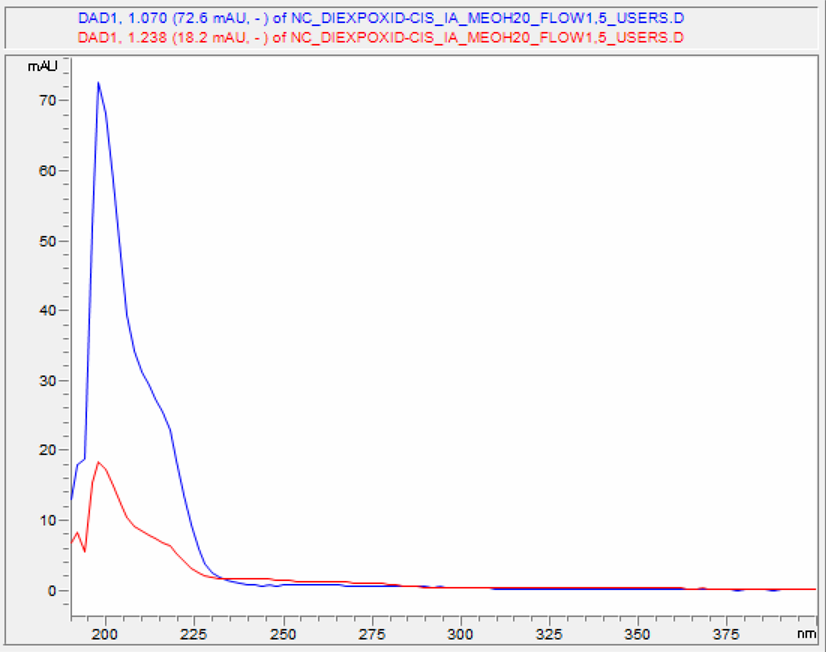


**5,8-diazido-6,7-dihydroxy-5,6,7,8-tetrahydronaphthalen-2-yl)ethan-1-one (2j)**

Racemic


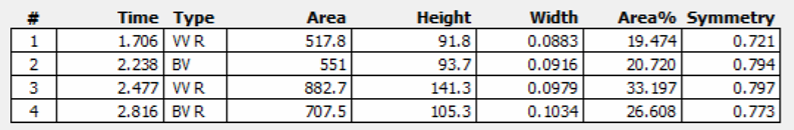


Conditions A
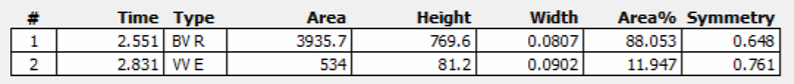


Conditions B


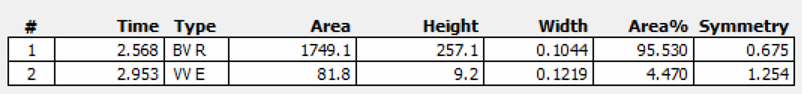


Conditions A
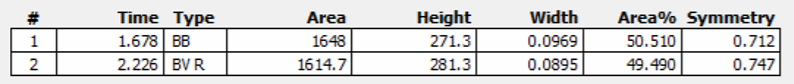


Conditions B


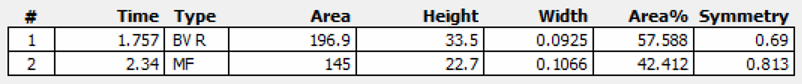


UV-vis racemic sample

**
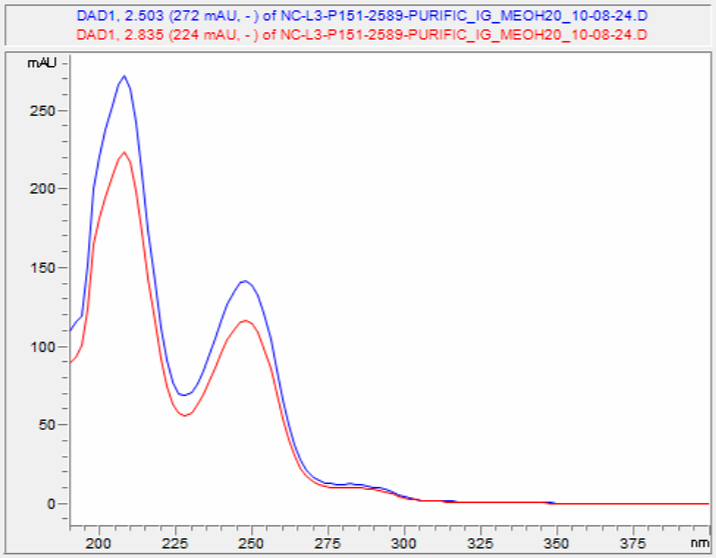
**

**
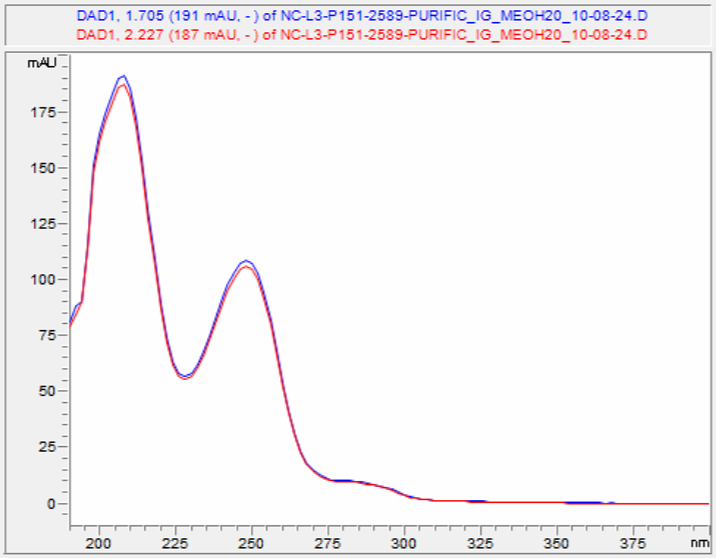
**

UV-vis chiral product

**
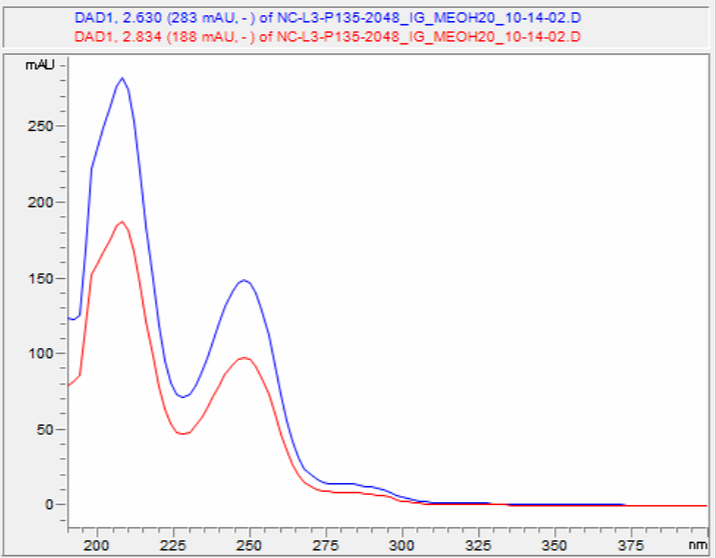
**

**
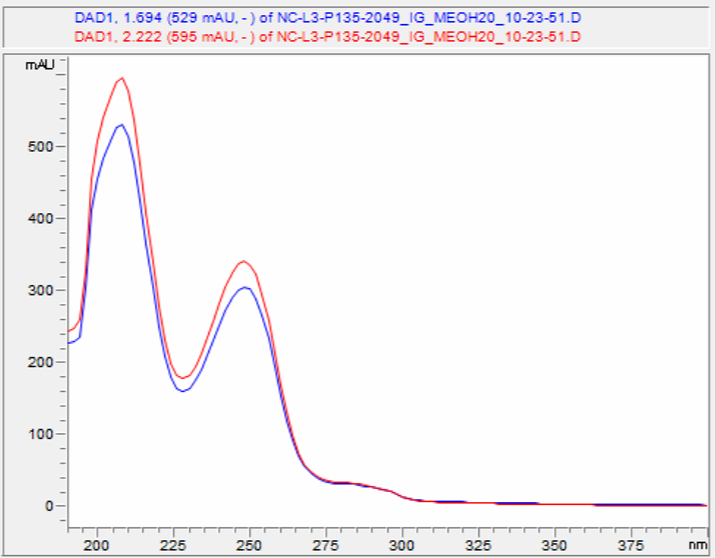
**

**5,8-diazido-6,7-dihydroxy-5,6,7,8-tetrahydronaphthalene-2-carbonitrile (2k)**

Racemic

**
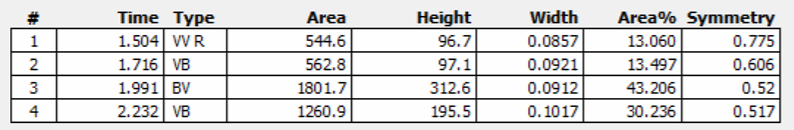
**

Conditions A**
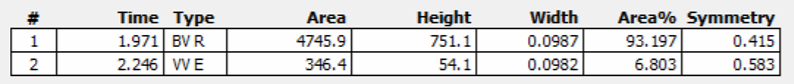
**

Conditions B

**
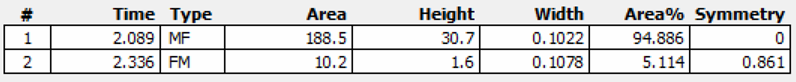
**

Conditions A**
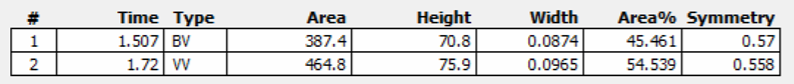
**

Conditions B


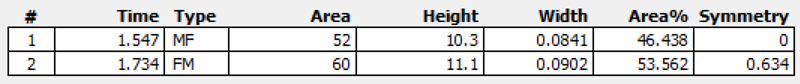


UV-vis racemic sample

**
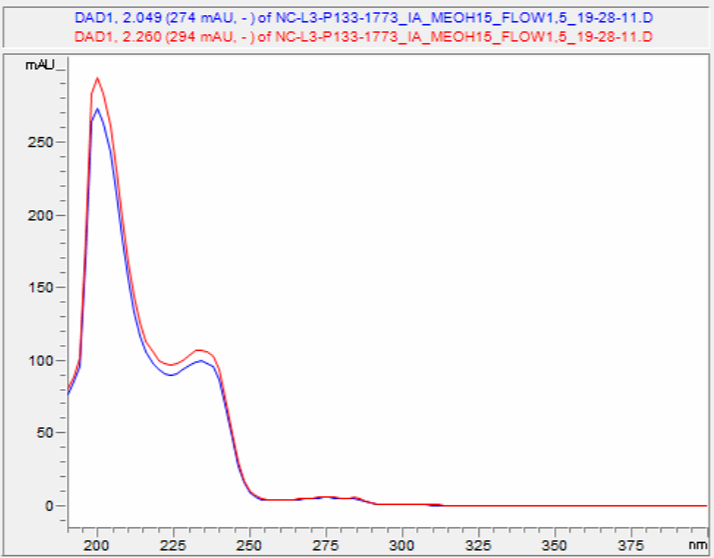
**

**
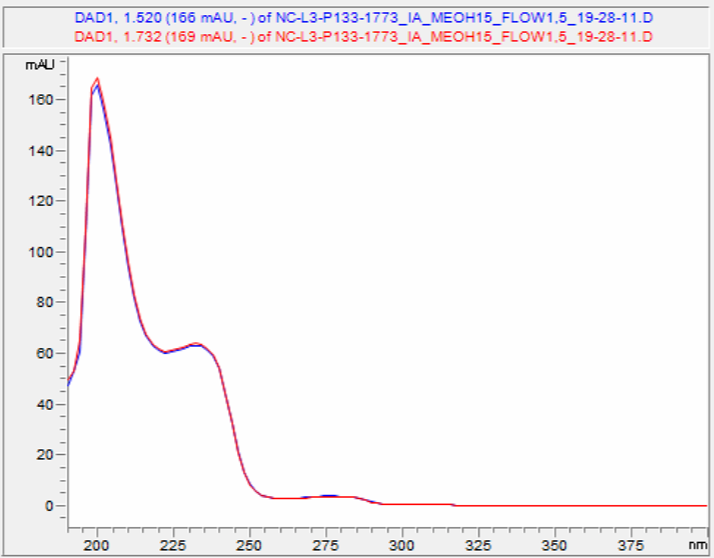
**

UV-vis chiral product

**
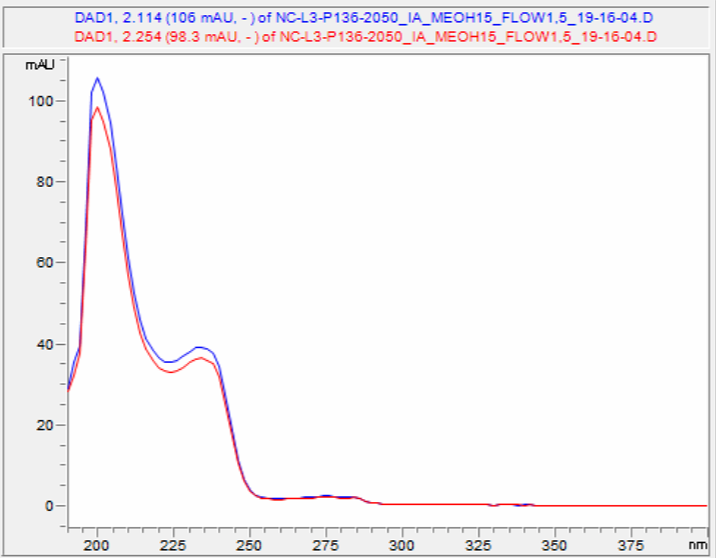
**

**
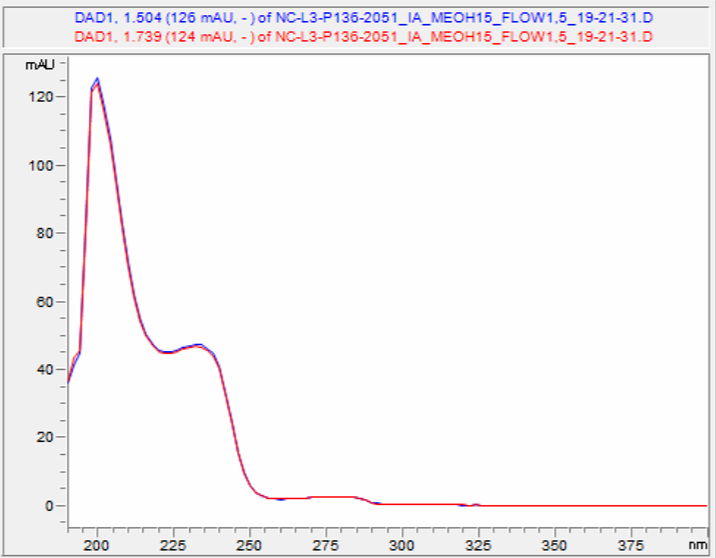
**

**5,8-diazido-6,7-dihydroxy-5,6,7,8-tetrahydronaphthalene-1-carbonitrile (2q)**

Racemic


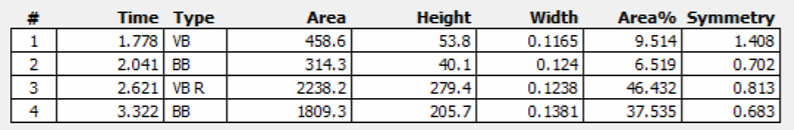


Conditions A


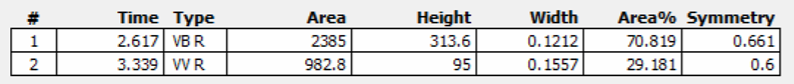


Conditions B


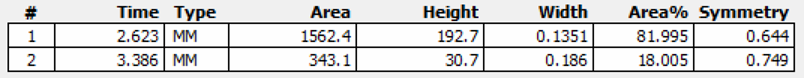


Conditions A


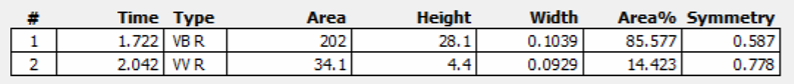


Conditions B


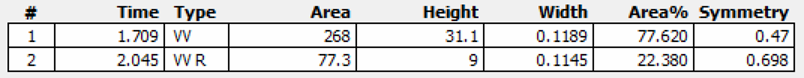


UV-vis racemic sample

**
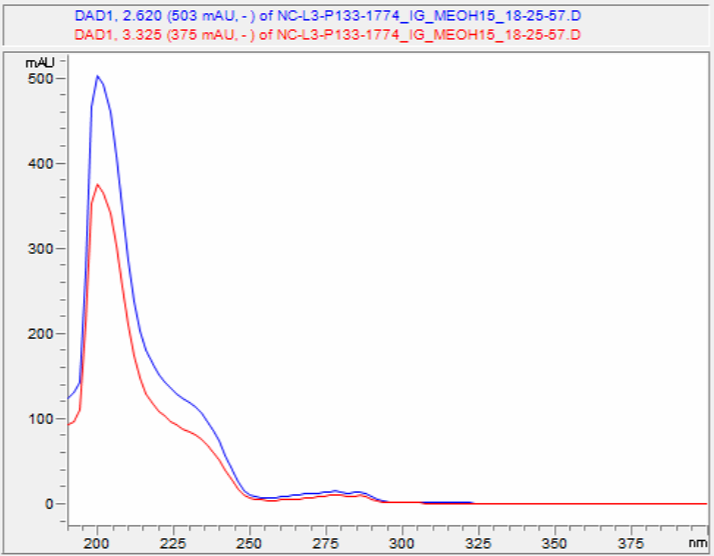
**

**
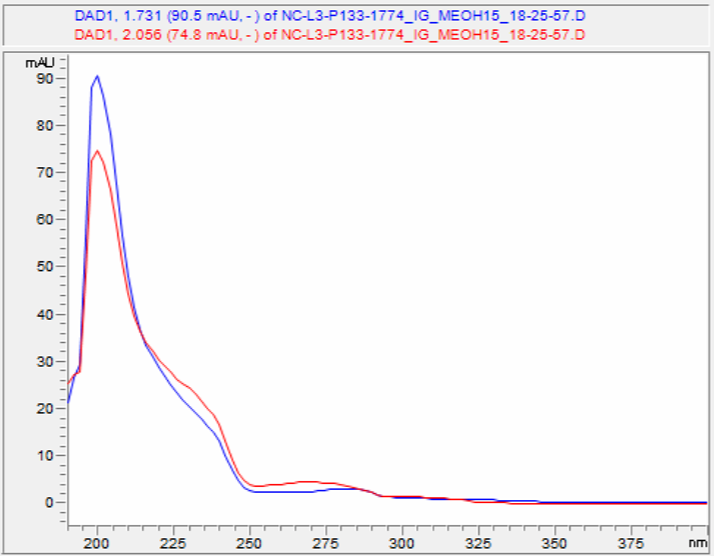
**

UV-vis chiral product

**
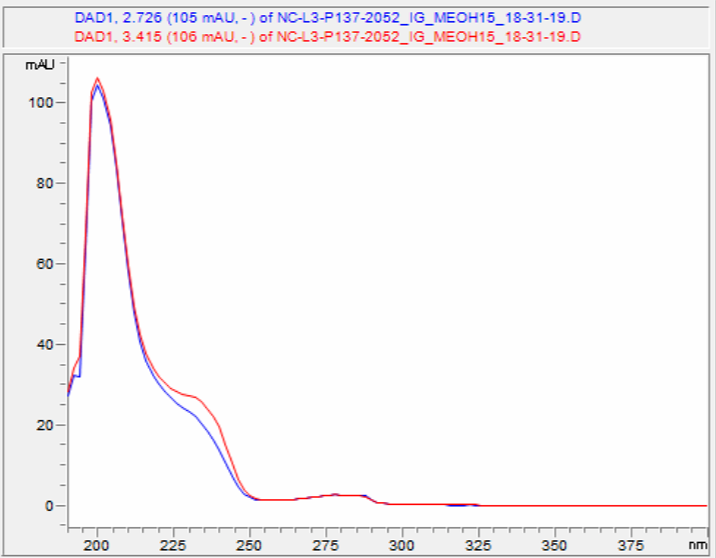
**

**(1α,2β,3α,4β)-1,4-diazido-6-ethyl-1,2,3,4-tetrahydronaphthalene-2,3-diol (2b*^anti^*)**

Racemic

Chiral product

UV-vis racemic sample

UV-vis chiral product

**(1α,2β,3α,4β)-1,4-diazido-1-ethyl-1,2,3,4-tetrahydronaphthalene-2,3-diol (2o*^anti^*)**

Racemic

Chiral product

UV-vis racemic sample

UV-vis chiral product

**(1α,2β,3α,4β)-1,4-diazido-1-methyl-1,2,3,4-tetrahydronaphthalene-2,3-diol (2q*^anti^*)**

Racemic

Chiral product

UV-vis racemic sample

UV-vis chiral product

**1,4-diazido-6-bromo-1,2,3,4-tetrahydronaphthalene-2,3-diol (2m)**

Racemic

Conditions A

Conditions B

Conditions A

Conditions B

UV-vis racemic sample

UV-vis chiral product

**1,4-diazido-5-bromo-1,2,3,4-tetrahydronaphthalene-2,3-diol (2r)**

Racemic

Conditions A

Conditions B

Conditions A

Conditions B

UV-vis racemic sample

UV-vis chiral product

**1,4-diazido-6-chloro-1,2,3,4-tetrahydronaphthalene-2,3-diol (2n)**

Racemic

Conditions A

Conditions B

Conditions A

Conditions B

UV-vis racemic sample

UV-vis chiral product

**1,4-diazido-6,7-dimethyl-1,2,3,4-tetrahydronaphthalene-2,3-diol (2c)**

Racemic

Chiral (*anti* isomer)

UV-vis racemic sample

UV-vis chiral product

**1,4-diazido-6-phenyl-1,2,3,4-tetrahydronaphthalene-2,3-diol (2d)**

Racemic

UV-vis racemic sample

UV-vis chiral product

**1,4-diazido-5-fluoro-1,2,3,4-tetrahydronaphthalene-2,3-diol (2s)**

Racemic

Conditions A

Conditions B

Conditions A

Conditions B

UV-vis racemic sample

UV-vis chiral product

**2-methyl-5,6,7,8-tetrahydronaphtho[5,6:7,8]bis(oxirene)-1-yl acetate (2t)**

Racemic

UV-vis racemic product

UV-vis chiral product

**2-methyl-4,5,6,7,-tetrahydronaphtho[4,5:6,7]bis(oxirene)-1-yl acetate (2u)**

Racemic

Chiral

UV-vis racemic product

UV-vis chiral product

**1,4-diazido-6-bromo-5-methyl-1,2,3,4-tetrahydronaphthalene-2,3-diol (2v)**

Racemic

Chiral

UV-vis racemic product

UV-vis chiral product

**1,2,3,4-tetrahydronaphthalene-2,3-diol (6a)**

Racemic

Chiral

UV-vis racemic product

UV-vis chiral product

**1,2,3,4-tetrahydronaphthalene-1,2,3,4-tetraol (6b)**

Racemic

Chiral

UV-vis racemic product

UV-vis chiral product

**3-methoxy-1,2,3,7-tetrahydronaphtho[1,2]oxiren-2-ol (6c)**

Racemic

Chiral

UV-vis racemic product

UV-vis chiral product

**1,4-bis(dimethylamino)-1,2,3,4-tetrahydronaphthalene-2,3-diol (6d)**

Racemic

Chiral

UV-vis racemic product

UV-vis chiral product

**1,4-dimethoxy-1,2,3,4-tetrahydronaphthalene-2,3-diol (6i)**

Racemic

Chiral

UV-vis racemic product

UV-vis chiral product

**1,4-bis(*p*-tolyloxy)-1,2,3,4-tetrahydronaphthalene-2,3-diol (6h)**

Racemic

Chiral

UV-vis racemic product

UV-vis chiral product

**1,4-dimethyl-1,2,3,4-tetrahydronaphthalene-2,3-diol (6g)**

Racemic

Chiral

UV-vis racemic product

UV-vis chiral product

**1,4-bis(butylthiol)-1,2,3,4-tetrahydronaphthalene-2,3-diol (6f)**

Racemic

Chiral

UV-vis racemic product

UV-vis chiral product

# Bibliography

(1) Choukairi Afailal, N., Borrell, M., Cianfanelli, M. and Costas, M. Dearomative syn-Dihydroxylation of Naphthalenes with a Biomimetic Iron Catalyst. *J. Am. Chem. Soc.* **2024**, *146*, 240-249.

(2) Chen, M. S. and White, M. C. A Predictably Selective Aliphatic C–H Oxidation Reaction for Complex Molecule Synthesis. *Science* **2007**, *318*, 783-787.

(3) Ottenbacher, R. V., Bryliakov, K. P. and Talsi, E. P. Non-Heme Manganese Complexes Catalyzed Asymmetric Epoxidation of Olefins by Peracetic Acid and Hydrogen Peroxide. *Adv. Synth. Catal.* **2011**, *353*, 885-889.

(4) Cussó, O., Garcia-Bosch, I., Font, D., Ribas, X., Lloret-Fillol, J. and Costas, M. Highly Stereoselective Epoxidation with H2O2 Catalyzed by Electron-Rich Aminopyridine Manganese Catalysts. *Organic Letters* **2013**, *15*, 6158-6161.

(5) Shen, D., Miao, C., Wang, S., Xia, C. and Sun, W. A Mononuclear Manganese Complex of a Tetradentate Nitrogen Ligand – Synthesis, Characterizations, and Application in the Asymmetric Epoxidation of Olefins. *European Journal of Inorganic Chemistry* **2014**, *2014*, 5777-5782.

(6) Cianfanelli, M., Olivo, G., Milan, M., Klein Gebbink, R. J. M., Ribas, X., Bietti, M. and Costas, M. Enantioselective C–H Lactonization of Unactivated Methylenes Directed by Carboxylic Acids. *J. Am. Chem. Soc.* **2020**, *142*, 1584-1593.

(7) Font, D., Canta, M., Milan, M., Cussó, O., Ribas, X., Klein Gebbink, R. J. M. and Costas, M. Readily Accessible Bulky Iron Catalysts exhibiting Site Selectivity in the Oxidation of Steroidal Substrates. *Angew. Chem. Int. Ed.* **2016**, *55*, 5776-5779.

(8) Cussó, O., Cianfanelli, M., Ribas, X., Klein Gebbink, R. J. M. and Costas, M. Iron Catalyzed Highly Enantioselective Epoxidation of Cyclic Aliphatic Enones with Aqueous H2O2. *J. Am. Chem. Soc.* **2016**, *138*, 2732-2738.

(9) Yan, G., Zhang, L. and Yu, J. Copper-Catalyzed Nitration of Arylboronic Acids with Nitrite Salts Under Mild Conditions: An Efficient Synthesis of Nitroaromatics. *Letters in Organic Chemistry* **2012**, *9*, 133-137.

(10) Barder, T. E., Walker, S. D., Martinelli, J. R. and Buchwald, S. L. Catalysts for Suzuki−Miyaura Coupling Processes:  Scope and Studies of the Effect of Ligand Structure. *J. Am. Chem. Soc.* **2005**, *127*, 4685-4696.

(11) Tsang, W. S., Griffin, G. W., Horning, M. G. and Stillwell, W. G. Chemistry of anti- and syn-1,2:3,4-naphthalene dioxides and their potential relevance as metabolic intermediates. *J. Org. Chem.* **1982**, *47*, 5339-5353.
